# Nuclear receptor coactivator 6 is a critical regulator of NLRP3 inflammasome activation and gouty arthritis

**DOI:** 10.1038/s41423-023-01121-x

**Published:** 2024-01-10

**Authors:** Kang-Gu Lee, Bong-Ki Hong, Saseong Lee, Naeun Lee, Seung-Whan Kim, Donghyun Kim, Wan-Uk Kim

**Affiliations:** 1 Center for Integrative Rheumatoid Transcriptomics and Dynamics, The Catholic University of Korea, Seoul, 06591 Republic of Korea; 2 Department of Biomedicine & Health Sciences, The Catholic University of Korea, Seoul, 06591 Republic of Korea; 3 Department of Pharmacology, Asan Medical Center, University of Ulsan College of Medicine, Seoul, 05505 Republic of Korea; 4 Bio-Medical Institute of Technology, University of Ulsan, Seoul, 05505 Republic of Korea; 5 Department of Microbiology and Immunology, Seoul National University College of Medicine, Seoul, 03080 Republic of Korea; 6 Institute of Infectious Diseases, Seoul National University College of Medicine, Seoul, 03080 Republic of Korea; 7 Division of Rheumatology, Department of Internal Medicine, The Catholic University of Korea, Seoul, 06591 Republic of Korea

**Keywords:** Nuclear receptor coactivator 6, Nuclear-to-cytoplasmic translocation, NLRP3 inflammasome, NACHT domain, Gouty arthritis, Inflammasome, Monocytes and macrophages, Inflammatory diseases

## Abstract

Transcriptional coactivators regulate the rate of gene expression in the nucleus. Nuclear receptor coactivator 6 (NCOA6), a coactivator, has been implicated in embryonic development, metabolism, and cancer pathogenesis, but its role in innate immunity and inflammatory diseases remains unclear. Here, we demonstrated that NCOA6 was expressed in monocytes and macrophages and that its level was increased under proinflammatory conditions. Unexpectedly, nuclear NCOA6 was found to translocate to the cytoplasm in activated monocytes and then become incorporated into the inflammasome with NLRP3 and ASC, forming cytoplasmic specks. Mechanistically, NCOA6 associated with the ATP hydrolysis motifs in the NACHT domain of NLRP3, promoting the oligomerization of NLRP3 and ASC and thereby instigating the production of IL-1β and active caspase-1. Of note, *Ncoa6* deficiency markedly inhibited NLRP3 hyperactivation caused by the *Nlrp3*^R258W^ gain-of-function mutation in macrophages. Genetic ablation of *Ncoa6* substantially attenuated the severity of two NLRP3-dependent diseases, folic-induced acute tubular necrosis and crystal-induced arthritis, in mice. Consistent with these findings, NCOA6 was highly expressed in macrophages derived from gout patients, and NCOA6-positive macrophages were significantly enriched in gout macrophages according to the transcriptome profiling results. Conclusively, NCOA6 is a critical regulator of NLRP3 inflammasome activation and is therefore a promising target for NLRP3-dependent diseases, including gout.

## Introduction

Transcriptional coactivators bind to transcription factors (TFs) in the nucleus to increase the rate of transcription. Although their activity is limited to protein-coding genes in the human genome, these coactivators participate in the efficient cellular mechanisms that control gene expression. Transcriptional coactivators function in two main ways: recruitment of transcription machinery to the promoter and activation of chromatin-remodeling enzymes [1]. They are usually localized in the nucleus and assembled in complex structural or functional modules [2]. Coactivator defects and dysfunction have been implicated in numerous human diseases, including metabolic disorders, type 2 diabetes, cardiovascular disease, and cancer, and thus, these transcriptional regulators are considered promising therapeutic targets [3].

Most coactivators enable rapid responses and adaptations to external stimuli, thereby protecting cells from death or damage [4], but their functional roles in immunity and disease are not fully understood. In our preliminary analysis using the STRING protein‒protein interaction database [5], we identified various coactivators that interact with TFs related to the immune response, which were identified using Gene Ontology biological process (GOBP) gene sets. These coactivators include the nuclear receptor coactivator (NCOA), mediator (MED) complex, coactivator-associated arginine methyltransferase (CARN1), and peroxisome proliferator-activated receptor-γ coactivator (PPARGC) (Fig. S1A). Of note, among the top 10 coactivators that highly interacted with immune response-related TFs, NCOA6 was unique in that it interacted with TFs that were involved in innate immunity but not adaptive immunity (Fig. S1A–C).

Nuclear receptor coactivator 6 (NCOA6), also known as ASC-2, NRC, TRBP, and PRIP [6], is a 250-kDa multifunctional coactivator protein involved in embryonic development, cell survival, metabolism, and homeostasis [7]. Like other coactivators, NCOA6 modulates transcriptional activity through interactions with TFs and nuclear receptors in the nucleus [8]. Specifically, NCOA6 contains an LxxLL motif, which interacts with a diverse array of nuclear receptors, including steroid receptors, retinoid receptors, thyroid hormone receptors, and vitamin D receptors [9]. Interestingly, there is recent evidence that NCOA6 also binds a variety of TFs involved in innate immunity and inflammation, including FOS, JUN, CREB, ATF-2, E2F-1, RB, TP53 and STAT2 [10, 11], and thereby can enhance their transcriptional activity. NCOA6 is highly expressed in the myeloid lineage, including macrophages and neutrophils [12, 13], suggesting its potential role in these cells. Moreover, NCOA6 has been implicated in the pathogenesis of diabetes mellitus, atherosclerosis, and some types of cancer [7], and innate immune cells, such as macrophages, are also involved in these diseases [14–16]. However, it remains unknown whether NCOA6 directly activates innate immune responses and promotes the development of inflammatory diseases.

Here, we propose the intriguing hypothesis that NCOA6 modulates innate immunity and/or inflammation. To address this hypothesis, we investigated the expression and function of NCOA6 in monocytes and macrophages, which play an important role in innate immunity and inflammation. Strikingly, we found that nuclear NCOA6 was translocated to the cytoplasm and then incorporated into the NLRP3 inflammasome complex, where its novel function of NLRP3 activation was detected. Mechanistically, cytoplasmic NCOA6 was associated with the NACHT domain of NLRP3, promoting NLRP3 inflammasome activity in vitro, and in vivo experiments using mouse models of NLRP3-dependent inflammatory diseases, including folic acid (FA)-induced acute tubular necrosis and monosodium urate (MSU) crystal-induced arthritis, confirmed the in vitro results. Moreover, NCOA6-regulated genes were significantly enriched in macrophages from patients with gouty arthritis. Overall, our data show that NCOA6 is a novel upstream regulator of NLRP3 activation and thus may be a promising target for NLRP3-dependent diseases.

## Results

### NCOA6 is expressed in monocytes and is upregulated by proinflammatory stimuli

Our preliminary results revealing that coactivators interact with TFs as well as previous studies showing high expression of NCOA6 in the myeloid lineage [13] and its disease association in mice [12] prompted us to examine the role of NCOA6 in innate immunity and inflammation. To investigation this, we first performed bioinformatics analysis using the protein–protein interaction database. Since NCOA6 is an intracellular coactivator that binds to other signaling regulators and TFs, we searched for the TFs that physically interact with NCOA6 using the STRING [5] database and identified 10 potential TFs. We then reconstructed a molecular network consisting of NCOA6 and the TFs, which showed that NCOA6 was located in the center of the network (Fig. S1D). The TFs were enriched in GOBPs that were primarily associated with the innate immune response, regulation of immune responses, and myeloid cell differentiation (Fig. S1E), suggesting a possible role of NCOA6 in these biological processes.

**Fig. 1:**
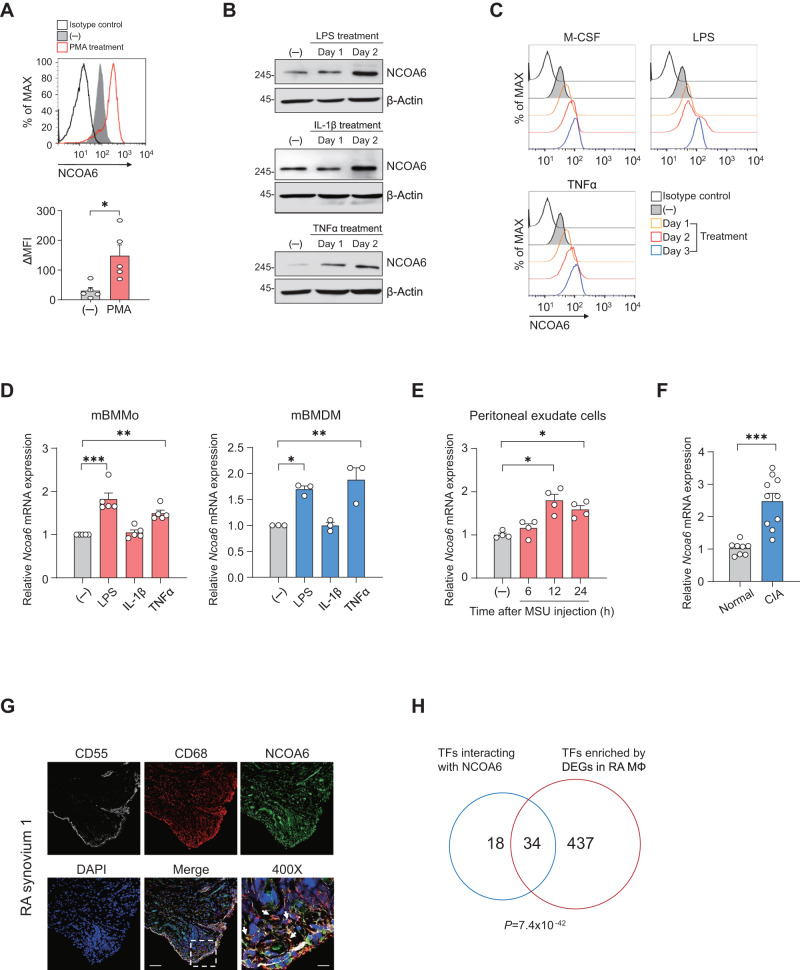
NCOA6 expression in monocytes and macrophages is upregulated by proinflammatory stimuli. **A** THP-1 cells were induced to differentiate into macrophages by treatment with PMA, and NCOA6 expression was determined using flow cytometry after 3 days. A representative plot is shown in the upper panel (*n* = 5). **B** THP-1 cells were left untreated or stimulated with LPS, IL-1β, or TNFα for the indicated duration, and cell lysates were immunoblotted with an anti-NCOA6 Ab. **C** Primary CD14^+^ monocytes isolated from the peripheral blood of healthy donors were stimulated with M-CSF, LPS, IL-1β or TNFα. NCOA6 was detected using flow cytometry. **D** Bone marrow-derived monocytes (BMMos) and bone marrow-derived macrophages (BMDMs) were left untreated or stimulated with LPS, IL-1β or TNFα, and *NCOA6* mRNA expression was analyzed using qPCR. Peritoneal exudate cells were obtained from C57BL/6 mice intraperitoneally injected with MSU crystals (**E**), and synovial tissues were isolated from DBA/1 mice with CIA (**F**). *Ncoa6* mRNA levels in peritoneal exudate cells and synovial tissues were analyzed using qPCR. **G** Representative immunofluorescence staining for NCOA6, CD68, and CD55 in the synovium of rheumatoid arthritis (RA) patients. The white arrows indicate CD68^+^NCOA6^+^ cells. The rectangular area in the merged image is magnified in the lower right panel. Scale bar=100 μm for the middle and 25 μm for the right. **H** Venn diagram showing the overlap between transcription factors (TFs) that interact with NCOA6 and TFs enriched in the differentially expressed genes (DEGs) in synovial macrophages from RA patients compared to healthy macrophages differentiated from peripheral monocytes (GSE49604). The immunoblotting and immunofluorescence results are representative of three independent experiments. β-Actin was detected as an internal control. Data in the bar graphs are shown as the mean ± SEM. Each dot represents an individual experiment (A) or mouse (**D**, **E**, **F**). *P < 0.05, **P < 0.01, and ***P < 0.001 compared to the controls by unpaired two-tailed t test (**A**, **F**) and one-way ANOVA (**D**, **E**)

Consistent with the bioinformatics analysis results, NCOA6 expression was upregulated upon the differentiation of THP-1 monocytes to macrophages, typical innate immune cells, in response to phorbol 12-myristate 13-acetate (PMA) stimulation (Fig. 1A). NCOA6 expression levels in THP-1 cells also time-dependently increased by treatment with the proinflammatory stimuli LPS, IL-1β, and TNFα (Fig. 1B). This induction of NCOA6 expression was reproduced in the primary monocytes obtained from healthy donors, in which M-CSF, LPS, IL-1β, and TNFα treatment substantially upregulated NCOA6 expression, as determined by flow cytometry and immunoblotting (Fig. 1C and Fig. S2A). Among the four alternatively spliced NCOA6 variants (α, β, γ, and δ) [17, 18], NCOA6α was the major isoform detected in CD14^+^ primary human monocytes (Fig. S3). Additionally, LPS and TNFα, but not IL-1β, also significantly upregulated *Ncoa6* mRNA expression in mouse monocytes and macrophages obtained from bone marrow (Fig. 1D and Fig. S2B). Together, these results demonstrate that NCOA6, specifically the NCOA6α isoform, is expressed in monocytes/macrophages and that its expression level is increased by proinflammatory stimuli in both humans and mice.

Next, we examined whether the observed upregulation in NCOA6 expression in response to proinflammatory stimuli also occurred in vivo under pathological conditions. To this end, we assessed *Ncoa6* expression in mouse models of acute inflammation (acute peritonitis) and chronic inflammation (collagen-induced arthritis). Significantly higher *Ncoa6* levels were noted in the peritoneal exudate cells from mice injected with MSU crystals compared with vehicle-treated control mice (Fig. 1E). In mice with collagen-induced arthritis, a chronic arthritis model that mimics rheumatoid arthritis (RA) [19], *Ncoa6* expression was much higher in the synovial tissue of arthritic mice than that in the synovial tissue vehicle-treated control mice (Fig. 1F). Moreover, CD68^+^ macrophages from RA synovial tissue frequently expressed the NCOA6 protein (*n* = 3, see both Fig. 1G and Fig. S2C). Moreover, in parallel with the functional enrichment analysis results of NCOA6-interacting TFs (Fig. S1E), there were numerous shared TFs between those interacting with NCOA6 and those enriched in the differentially expressed genes (DEGs) that we previously identified in the inflammatory macrophages of RA patients (Fig. 1H) [20].

In summary, the data show that NCOA6 significantly interacts with TFs involved in innate immunity, and its expression is induced in activated monocytes/macrophages and inflamed tissues, which suggests the proinflammatory role of NCOA6 in monocytes and macrophages.

### NCOA6 colocalizes with the NLRP3 inflammasome and triggers innate immune responses in macrophages

**Fig. 2:**
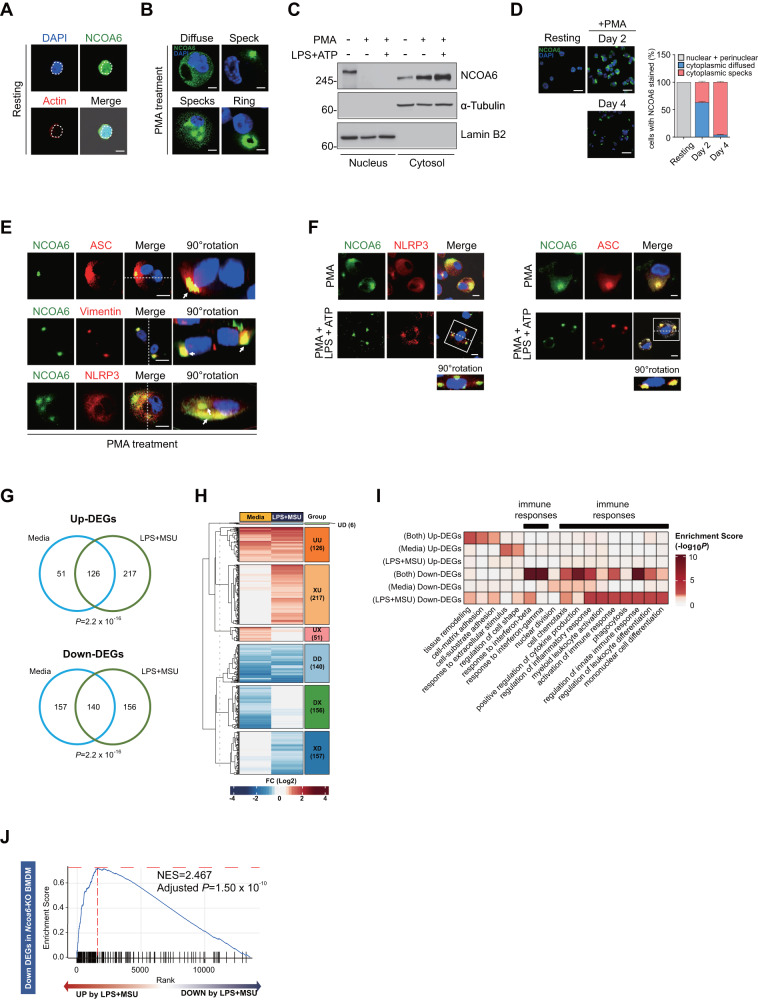
Nuclear-to cytoplasmic translocalization of NCOA6 in macrophages and its potential role in innate immunity and inflammation. **A**–**F** THP-1 cells were left untreated or treated with PMA. The PMA-treated THP-1 cells were left unstimulated or stimulated with LPS for 4 h and with ATP for an additional 30 min. Representative immunofluorescence staining for NCOA6, actin, and DAPI in resting cells (**A**) and PMA-treated THP-1 cells (**B**). The dotted circle indicates the nuclear margin. Scale bars, 20 μm. The cellular localization of NCOA6 was analyzed by immunoblotting of nuclear and cytoplasmic fractions (**C**) and by immunofluorescence staining (**D**, left panel). Scale bars, 50 μm. The localization and cytoplasmic form of NCOA6 were analyzed in at least 200 cells for each group (**D**, bar graphs in the right panel). Representative immunofluorescence staining showing the colocalization of NCOA6, ASC, vimentin, and NLRP3 (**E**, **F**). Cells with colocalized staining (merge) were rotated by 90° along the dashed line and imaged from the side view (bottom). Scale bars, 20 μm. **G** Venn diagram illustrating the upregulated DEGs (Up-DEGs) and downregulated DEGs (Down-DEGs) in *Ncoa6*-KO BMDMs (*n* = 2) compared to wild-type (WT) BMDMs (*n* = 2) stimulated without or with LPS + MSU. **H** Heatmap showing the six groups formed by the 847 DEGs in *Ncoa6*-KO BMDMs compared to their respective WT BMDMs. The letters U, D, and X in the right colored box denote upregulation, downregulation, and no expression change, respectively. The UX and DX groups indicate up- and downregulation only with medium alone, the XU and XD groups up- and downregulation only with LPS + MSU, and the UU and DD groups up- and downregulation both with media alone and with LPS + MSU, respectively. The UD group was excluded from gene ontology (GO) enrichment analysis because it only has 6 genes and lacks biological relevance. The number of DEGs is shown in parentheses in the colored box. **I** Heat plot visualizing the GOBP terms enriched by the genes in the UU, UX, XU, DD, DX, and XD groups; the UD group containing the 6 DEGs was excluded. **J** GSEA plot demonstrating significant enrichment between downregulated DEGs in *Ncoa6*-KO BMDMs and upregulated genes in WT BMDMs stimulated with LPS + MSU compared to media alone. The horizontal and vertical dotted red lines indicate the highest enrichment score and leading edge, respectively

Nuclear localization is required for coactivators to regulate gene expression [17, 21]. NCOA6α, but not NCOA6δ or NCOA6γ, is mainly located in the nucleus to interact with multiple TFs and nuclear receptors in HeLa cells and embryonic fibroblasts [18]. Considering that the STRING analysis showed that NCOA6 interacted with several TFs in the nucleus, we examined whether NCOA6 is primarily localized in the nucleus of monocytes, as would be expected for a transcriptional coactivator. Immunocytochemistry analysis using anti-NCOA6 Abs showed that, as expected, NCOA6 was localized in the nucleus of resting THP-1 monocytes (Fig. 2A). Surprisingly, after PMA stimulation, NCOA6 was rapidly redistributed from the nucleus to the cytoplasm, exhibiting diverse cytoplasmic localization patterns, including diffuse homogeneous staining, aggregated specks (single or multiple), and ring-shaped patterns (Fig. 2B and Fig. S4). The nuclear-to-cytoplasmic translocation of NCOA6 in response to PMA was confirmed by western blot analysis after the subcellular fractionation of PMA-stimulated THP-1 monocytes (Fig. 2C). Whereas a single band corresponding to NCOA6 was detected in the lysates from primary human monocytes (Fig. S2A**)**, two bands corresponding to NCOA6 with different molecular weights were detected in the lysates from THP-1 monocytes, and the molecular weight of nuclear NCOA6 was slightly greater than that of cytoplasmic NCOA6 (Fig. 2C). Interestingly, upon PMA stimulation, the predominant cytoplasmic pattern changed in a time-dependent manner from diffuse homogeneous localization to aggregated specks, and at 4 days after PMA stimulation, almost all THP-1 cells possessed only cytoplasmic specks (Fig. 2D).

To understand the function of cytoplasmic NCOA6 and its potential role in inflammation/innate immunity, we explored whether NCOA6 is localized in subcellular organelles using double immunofluorescence staining with anti-NCOA6 Abs and Abs specific for different subcellular organelles. The results showed that NCOA6 was not found in the Golgi, ER or lysosomes (Fig. S4). Because subcellular protein complexes in the cytoplasm, including inflammasomes, autophagosomes, and aggresomes, are frequently observed as aggregated specks, we further investigated whether NCOA6 is present in the complexes by examining the cells with aggregated specks in greater detail (Fig. 2B). This analysis clearly showed the colocalization of NCOA6 with NLRP3, ASC, and vimentin, the major components of the NLRP3 inflammasome (Fig. S5A, B, and Fig. 2E); 3-dimensional images are provided in Videos S1 and S2. The translocation of ASC from the nucleus to the cytoplasm in response to PMA was previously reported [22], and this event was coincident with the cytoplasmic translocation of NCOA6 (Fig. S5A, B, and Fig. 2E), suggesting that the translocation of NCOA6 and ASC might follow one another.

Moreover, the colocalization of NCOA6 with NLRP3 in PMA-differentiated THP-1 cells became more prominent upon additional stimulation of the cells with ATP or LPS plus ATP but not by LPS only (Fig. 2F, Fig. S5D). The further increase in the nuclear-to-cytoplasmic translocation of NCOA6 in response to LPS plus ATP was also confirmed by western blotting (Fig. 2C). However, there was no aggresome formation after treatment with an anti-NCOA6 Abs and the aggresome activator MG132 [23]. However, aggresome formation was evident with Abs specific for vimentin, an aggresome marker [24] (Fig. S5C), indicating that the colocalization of NCOA6 and NLRP3 seemed to be independent of aggresomes. In other words, MG132 disrupted speck formation and led to a diffuse homogeneous pattern in THP-1 cells stained with anti-NCOA6 or anti-ASC Abs (Fig. S5C); this finding is presumably related to the inhibition of inflammasome formation by MG132 [25]. Taken together, these data demonstrate that NCOA6 translocates from the nucleus to the cytoplasm during monocyte-to-macrophage differentiation and is then incorporated into the NLRP3 inflammasome, which suggests that NCOA6 is linked to the NLRP3 inflammasome complex.

Subcellular colocalization of NCOA6 and NLRP3 does not necessarily indicate that they are functionally correlated. Moreover, since the role of NCOA6 in monocytes and macrophages is largely unknown, it is still unclear whether NCOA6 plays a major or minor role in NLRP3-associated biological processes within these cells. To systematically understand the function of NCOA6 in monocytes/macrophages, we conducted global transcriptome profiling of bone marrow-derived macrophages (BMDMs) isolated from *Ncoa6*^*fl/fl*^*LysM*^*CRE*^ mice, which specifically do not express *Ncoa6* (Fig. S6) and *Ncoa6*^*fl/fl*^ mice that express *Ncoa6* (Fig. S6) and compared the resulting profiles. In total, 847 DEGs (394 upregulated and 453 downregulated DEGs) between *Ncoa6*-knockout (KO, −/−) and *Ncoa6*-sufficient (+/+) BMDMs in the absence or presence of LPS plus MSU crystals (LPS + MSU) were identified by RNA sequencing (Fig. 2G, H and Table S1). The DEGs were divided into 6 groups depending on the pattern of up- and downregulation after stimulation without or with LPS + MSU: (1) up- or downregulation only with medium alone, (2) up- or downregulation only with LPS + MSU, and (3) up- or downregulation both with media alone and with LPS + MSU (Fig. 2H). Functional enrichment analysis of 847 DEGs demonstrated that a variety of cellular processes, including mononuclear cell differentiation, responses to interferon (IFN)-β or IFN-γ, chemotaxis, phagocytosis, positive regulation of cytokine production, and regulation of the innate immune response, were significantly enriched in the downregulated DEGs but not the upregulated DEGs and that the major function of NCOA6 in macrophages is to modulate cellular processes related to innate immunity and inflammation. (Fig. 2I and Table S2). Moreover, gene set enrichment analysis (GSEA) revealed that the downregulated DEGs (*see* Fig. 2H) in *Ncoa6*-KO BMDMs (with media alone and/or with LPS + MSU) were significantly upregulated in *Ncoa6*-sufficient BMDMs activated with LPS + MSU compared to BMDMs in media alone (Fig. 2J and Fig. S7), indicating that NCOA6-regulated genes are closely related to LPS + MSU (an inflammasome activator) stimulation.

Collectively, the transcriptome data, together with immunofluorescence images, suggest that after translocation to the NLRP3 inflammasome complex, cytoplasmic NCOA6 plays a major role in activating innate immune responses, presumably via NLRP3 activation.

### NCOA6 deficiency reduces NLRP3 inflammasome activity

**Fig. 3:**
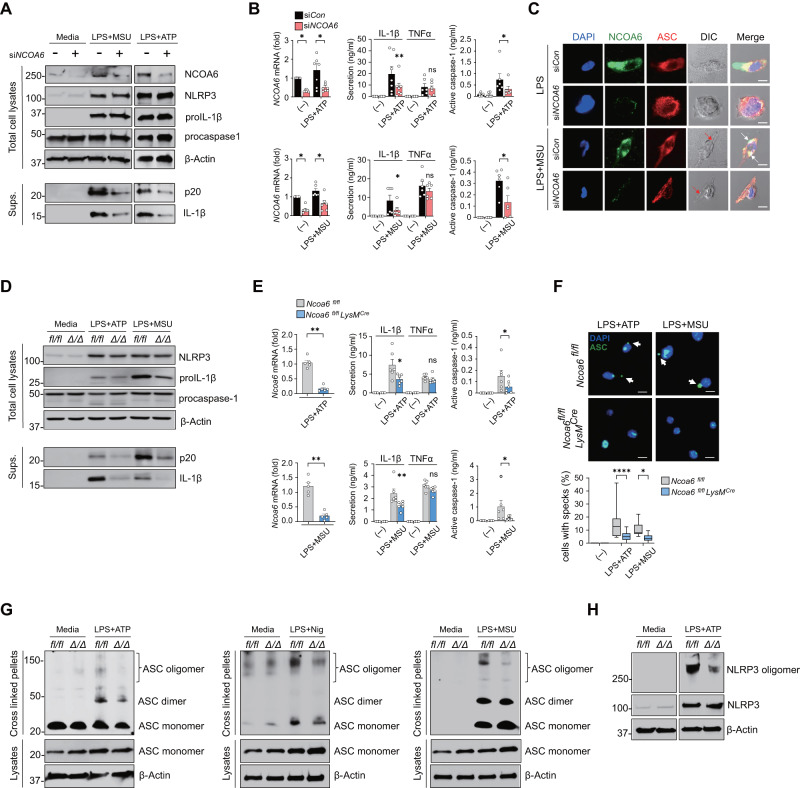
NCOA6 promotes NLRP3 inflammasome activity. **A**–**C** Human monocytes from healthy donors transfected with *NCOA6* siRNA or control siRNA were primed with LPS for 6 h, followed by stimulation with ATP for 30 min or MSU crystals for 2 h. Cell lysates and culture supernatants were immunoblotted with the indicated antibodies. Sups indicates supernatants (**A**). *NCOA6* mRNA levels were analyzed by real-time qRT–PCR (**B** left panel). The amounts of secreted IL-1β, TNFα, and active caspase-1 in the supernatant were measured using ELISA (**B** middle and right panel). Each dot represents an individual donor. Representative immunofluorescence staining for NCOA6, ASC, and DAPI (**C**). The white arrows (Merge) indicate ASC specks, and the red arrows (DIC) indicate phagocytosed MSU crystals. DIC, differential interference contrast. Scale bars, 20 μm. **D**–**H** BMDMs derived from *Ncoa6*^*fl/fl*^ (*fl/fl*) mice and *Ncoa6*^*fl/fl*^*LysM*^*CRE*^ (*Δ*/*Δ*) mice (*n* = 6 per group) were primed with LPS for 4 h, followed by stimulation with ATP for 30 min, MSU crystals for 2 h, or nigericin (Nig) for 2 h. Cell lysates and culture supernatants were immunoblotted with the indicated antibodies. Sups denotes supernatants. **D** *NCOA6* mRNA levels were analyzed using real-time qPCR (**E**, left panel). The levels of secreted IL-1β, TNFα, and active caspase-1 in the supernatant were determined using ELISA (**E** middle and right panels). Each dot indicates an individual mouse. Representative immunofluorescence images for ASC and DAPI (**F** upper panel). The white arrows indicate ASC specks. Scale bars, 20 μm. The ratio of cells with ASC specks was calculated by randomly counting at least 20 cells per field, and the results are presented as a bar graph (**F** lower panel). To detect NLRP3 oligomerization or ASC oligomerization, cells were crosslinked using disuccinimidyl suberate. Cell lysates as input controls were immunoblotted with antibodies against NLRP3, ASC and β-actin (**G**, **H**). The immunoblotting and immunocytochemistry results are representative of at least three independent experiments with similar results. *P < 0.05, **P < 0.01, and ****P < 0.0001 by paired two-tailed *t* test

We next sought to confirm that NCOA6 actually triggers NLRP3 inflammasome activation in monocytes and macrophages. The NLRP3 inflammasome requires two steps for its full activation [26]. Signal 1, the first step, is inflammasome priming in response to PAMPs and DAMPs, such as LPS, to reach the threshold level of NLRP3 and pro-IL-1β mRNA transcription [27]. Signal 2, the second step, is essential for NLRP3 inflammasome assembly and can be activated by damage-associated molecular patterns (DAMPs); this step leads to ASC speck formation and ultimately to the caspase-1-dependent maturation of IL-1β [28]. To confirm that NCOA6 regulates the NLRP3 inflammasome, human peripheral monocytes from healthy individuals were transfected with siRNA targeting *NCOA6* (si*NCOA6*) and then subjected to the two-step process of NLRP3 inflammasome activation. The results showed that si*NCOA6* transfection had no significant effect on the mRNA expression of the inflammasome-related genes *NLRP3, PYCARD, CASP1*, and *IL1B* in response to treatment with LPS + ATP or LPS + MSU, the well-known NLRP3 stimulators [29, 30]. (Fig. S8A, B), which indicates that NCOA6 only minorly affects the mechanism of inflammasome priming (Signal 1) in human monocytes. In accordance with this finding, si*NCOA6* transfection did not alter pro-IL-1β protein or NLRP3 protein expression (Fig. 3A). However, compared to those of control cells, the lysates and/or culture supernatants of si*NCOA6*-transfected monocytes had markedly lower levels of mature IL-1β and active caspase-1 (Fig. 3A, B), as determined by western blot analysis and ELISA. These findings demonstrate that NCOA6 is needed for the caspase-1-dependent maturation of IL-1β in human monocytes (i.e., Signal 2 for nontranscriptional activation of the NLRP3 inflammasome). In support of this finding, ASC speck formation, an immunocytological hallmark of inflammasome activation [31], was markedly diminished in *NCOA6*-knockdown monocytes compared to control siRNA-transfected monocytes when stimulated with LPS only or LPS + MSU (Fig. 3C). Moreover, the production of TNFα and IL-6, inflammasome-independent cytokines, was not significantly affected by si*NCOA6* (Fig. 3B and Fig. S9A). However, *Ncoa6* deficiency did not affect caspase-1 release by poly (dA:dT), which triggers the AIM2 inflammasome, or by *Salmonella* flagellin, an NLRC4 inflammasome activator (Fig. S10), suggesting that NCOA6 selectively modulates the activity of the NLRP3 inflammasome but not that of the AIM2 inflammasome or the NLRC4 inflammasome.

Consistent with the human data, there was no difference in the mRNA expression of *Nlrp3, Pycard*, and *Casp1* between *Ncoa6-*KO BMDMs from *Ncoa6*^*fl/fl*^ *LysM*^*CRE*^ mice and *Ncoa6-*sufficient BMDMs from *Ncoa6*^*fl/fl*^ mice after stimulation with LPS + ATP or LPS + MSU; *Il1b* mRNA levels, however, were modestly but significantly reduced in *Ncoa6-*KO BMDMs (Fig. S8C, D). These data suggest that murine NCOA6 can regulate IL-1β transcription but not caspase-1 transcription as a coactivator. Moreover, procaspase-1 and pro-IL-1β protein levels were unchanged and somewhat reduced by *Ncoa6* deficiency, respectively (Fig. 3D), which supports the notion that NCOA6 is involved in murine inflammasome priming to a minor extent. However, similar to *NCOA6*-deficient human monocytes, *Ncoa6-*KO BMDMs stimulated with LPS + ATP or LPS + MSU secreted much lower levels of IL-1β and active caspase-1 but not TNFα or IL-6 than control BMDMs (Fig. 3E and Fig. S9B), confirming the requirement of NCOA6 for the caspase-1-dependent maturation of IL-1β. ASC speck formation was also markedly decreased by *Ncoa6-*KO (Fig. 3F). Concurrently, the formation of ASC oligomers that constitute ASC specks [31] was almost completely blocked in *Ncoa6-*KO BMDMs in the presence of NLRP3 inflammasome activators, including LPS + ATP, LPS+nigericin, and LPS + MSU (Fig. 3G).

Colocalization of NCOA6 with NLRP3 (Fig. 2F) and NCOA6’s regulation of ASC oligomerization (Fig. 3G) suggests that NCOA6 affects events upstream of ASC oligomer formation, such as NLRP3 oligomerization. To address this, we conducted an NLRP3 oligomerization assay and found a dramatic reduction in NLRP3 oligomer formation in *Ncoa6-*KO BMDMs (Fig. 3H), which signifies that NCOA6 induces ASC oligomerization by facilitating NLRP3 oligomerization in macrophages. However, the decrease in NCOA6 levels did not influence NLRP3 expression in mouse macrophages (Fig. 3D and Fig. S8), which is consistent with the findings in human macrophages (Fig. 3A). Together, the NCOA6-directed regulation of inflammasome activity was reproduced in murine macrophages with some minor differences.

Overall, these data demonstrate that NCOA6 enhances NLRP3 inflammasome activity by modulating Signal 2 (predominant effect) and Signal 1 (partial effect noted only in the mouse system), thereby promoting the production of mature IL-1β and active caspase-1.

### NCOA6 is associated with the NACHT domain of NLRP3 in the cytoplasm

**Fig. 4:**
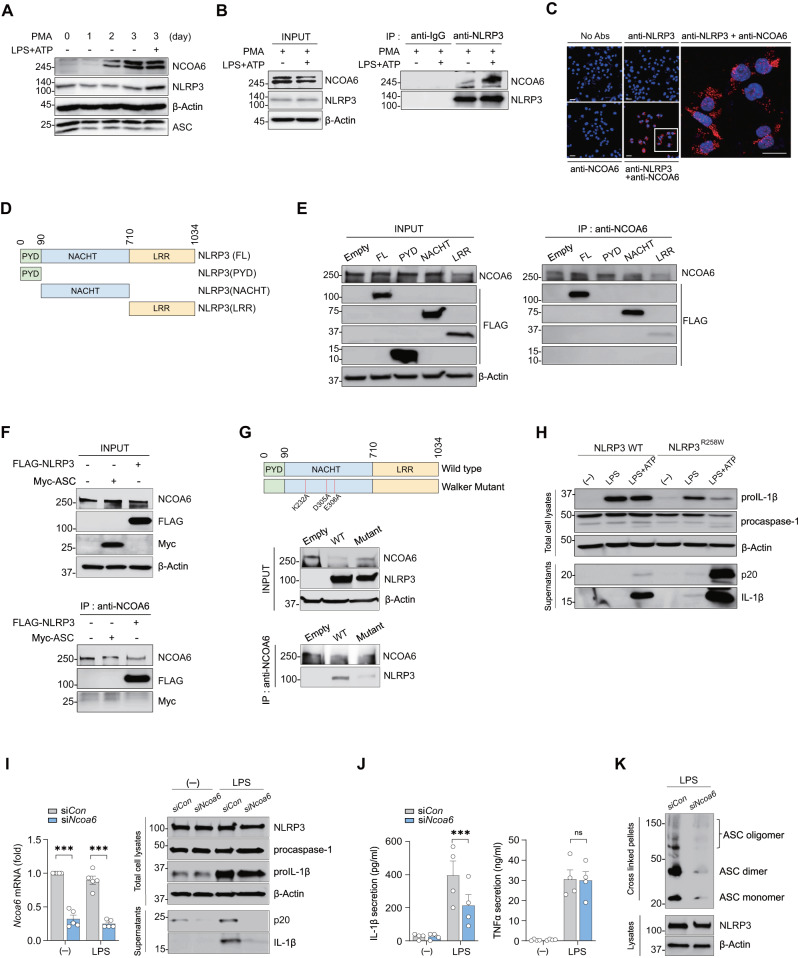
Association of NCOA6 with the NACHT domain of NLRP3. **A**, **B** THP−1 cells were stimulated with PMA for the indicated duration, primed with LPS, and treated with ATP. Cell lysates were immunoblotted with the indicated antibodies (**A**). Lysates were analyzed by NLRP3 pulldown and immunoblotting with antibodies against NCOA6 and NLRP3 (**B**). **C** Proximity ligation assay (PLA) in PMA-stimulated THP-1 cells primed with LPS (100 ng ml^−1^) for 4 h, followed by ATP (5 mM) treatment for 30 min. Red fluorescent dots indicate sites of interactions between NCOA6 and NLRP3 proteins. The rectangular area in the middle panel is magnified to the right panel. Scale bars, 20 μm. **D** Schematic of full-length NLRP3 and the truncated domain constructs. The number refers to the human NLRP3 sequence. **E**, **F** Wild-type or mutant NLRP3 (PYD, NACHT or LRR domain) and/or Myc-tagged ASC were overexpressed in HEK293T cells. Cell lysates were immunoprecipitated with the NCOA6 antibody and then immunoblotted with the indicated antibodies: FL=full length. **G** Decrease in the NCOA6-NLRP3 interaction by Walker A and B motif point mutations in the NACHT domain. HEK293T cells were transfected with the DNA constructs for wild-type NLRP3 (WT) and mutant NLRP3 (Mutant) and then subjected to immunoprecipitation with anti-NCOA6 Ab, followed by immunoblotting with the indicated antibodies. A schematic of the NLRP3 Walker mutant is shown in the top panel. **H** Immortalized BMDMs (iBMDMs) expressing wild-type NLRP3 (NLRP3 WT) or NLRP3^R258W^ were primed with LPS followed by ATP treatment. Cell lysates and culture supernatants were immunoblotted with the indicated antibodies. **I**, **J** Upstream regulatory effect of NCOA6 on NLRP3 inflammasome activity. NLRP3^R258W^ iBMDMs were transfected with *Ncoa6* siRNA or control siRNA for 24 h and then stimulated with LPS for 6 h. *Ncoa6* mRNA expression was analyzed by real-time qPCR (**I** left panel). Cell lysates and culture supernatants were immunoblotted with the indicated antibodies (**I** right panel). Immunoprecipitation and immunoblotting results are representative of at least three independent experiments. ELISA for IL-1β and TNF-α in culture supernatants (**J**). **K** ASC oligomerization in LPS-stimulated NLRP3^R258W^ iBMDMs transfected with *Ncoa6* siRNA or control siRNA. Lysates or cross-linked pellets were immunoblotted with the indicated antibodies. The results are representative of three independent experiments. ***P < 0.01 by unpaired two-tailed t test (H) and two-way ANOVA corrected for multiple comparisons

We next investigated how NCOA6 mechanistically regulates NLRP3 activity. First, we analyzed NCOA6 and NLRP3 expression over time in THP-1 monocytes sequentially activated by priming with PMA followed by stimulation with LPS + ATP. NCOA6 expression was upregulated even in the cells that were only primed, whereas NLRP3 expression was upregulated only after the additional stimulation of LPS + ATP (Fig. 4A), suggesting earlier involvement of NCOA6 in the inflammasome activation process of THP-1 cells. Moreover, an immunoprecipitation assay using anti-NLRP3 Abs in ATP- or LPS + ATP-stimulated THP-1 cells clearly demonstrated that endogenous NLRP3 coimmunoprecipitated with NCOA6 (Fig. 4B, Fig. S11A), indicating that NLRP3 and NCOA6 are likely to interact. To support this, we conducted a proximity ligation assay (PLA) using anti-NCOA6 and anti-NLRP3 antibodies. PLA is a powerful experimental tool that allows in situ detection of protein interactions with high specificity and sensitivity [32]. The results showed that robust red fluorescence was detected in the cytoplasm of the LPS + ATP-stimulated THP-1 cells only when these two antibodies were simultaneously used (Fig. 4C); such robust red fluorescence was not observed after a single treatment with either anti-NCOA6 or anti-NLRP3 Ab (Fig. 4C). Moreover, the number of cytoplasmic spots was markedly increased by the inflammasome activator LPS + ATP (Fig. S12). Together, these findings demonstrate that there may be a molecular interaction between NCOA6 and NLRP3 in macrophages.

Potassium efflux is a common upstream signaling event triggered by most NLRP3 stimuli, such as ATP, and it specifically promotes the interaction of NEK7 with NLRP3 [33, 34]. To test whether potassium efflux affects the association of NCOA6 with NLRP3, we carried out an immunoprecipitation assay of NLRP3 in THP-1 cells stimulated with ATP under the condition of elevated extracellular potassium, which inhibits potassium efflux. In line with an earlier study [34], the association of NEK7 with NLRP3 was reduced upon the addition of extracellular potassium, specifically KCl (Fig. S11B). Moreover, the association of NCOA6 with NLRP3 in the same cells was not significantly repressed (Fig. S11B). Furthermore, *Ncoa6* deficiency failed to downregulate *Nek7* expression in BMDMs and did not mitigate the molecular interaction of NEK7 with NLRP3 (Fig. S11C, D). Together, these findings support the notion that NCOA6-mediated NLRP3 inflammasome activation may be independent of potassium efflux and NEK7.

We next wondered which domain of NLRP3 specifically interacts with NCOA6. The structure of NLRP3 consists of a pyrin domain (PYD), a central nucleotide-binding and oligomerization (NACHT) domain, and a C-terminal leucine-rich repeat (LRR) domain (Fig. 4D); the N-terminal PYD is necessary for interaction with the adapter molecule ASC [35]. Further mapping studies using three truncated NLRP3 constructs, each consisting of only a single domain, and anti-NCOA6 Abs showed that NCOA6 coprecipitated strongly with the NACHT domain and modestly with the LRR domain but showed no interaction with the PYD (Fig. 4E, Fig. S13), indicating that NCOA6 is associated with the NACHT domain of NLRP3. We also questioned whether NCOA6 interacts with ASC. Immunoprecipitation assays using anti-NCOA6 Abs showed that NCOA6 did not coprecipitate with Myc-ASC but did strongly coprecipitate with FLAG-NLRP3, suggesting no interaction between NCOA6 and ASC (Fig. 4F, Fig. S13). Moreover, in BMDMs, *Ncoa6* deficiency did not reduce the interaction between NLRP3 and ASC, as determined by an immunoprecipitation assay (Fig. S11D). Based on the immunoprecipitation data using both anti-NLRP3 Abs and anti-NCOA6 Abs, we concluded that NCOA6 may interact with the NACHT domain of NLRP3 without associating with ASC to activate the NLRP3 inflammasome.

The NACHT domain has nucleotide-binding sites of Walker A and B motifs, which have ATPase activity critical to NLRP3 oligomerization [36]. Based on the findings that NCOA6 is specifically related to the NACHT domain, we wondered whether Walker A and B motifs in the NACHT domain are involved in NCOA6-induced NLRP3 oligomerization. To this end, we first reconstructed an NLRP3 mutant at the Walker A and B motifs (K232A, D305A, and E306A) that lacks ATP hydrolysis (Fig. 4G, upper panel). Subsequently, we overexpressed wild-type NLRP3 versus mutant NLRP3 in HEK293T cells to investigate whether such mutations repress the NLRP3 interaction with NCOA6. The results demonstrated the interaction of NCOA6 with wild-type NLRP3, which was not observed with mutant NLRP3 (Fig. 4G, the middle and lower panel). These data, together with an earlier report [36], suggest that Walker A and B motifs with ATPase activity serve as a critical site for the interaction between NLRP3 and NCOA6 and that ATP hydrolysis of the NACHT domain might be needed for NCOA6-dependent NLRP3 oligomerization.

We finally sought to clarify how important NCOA6 is for NLRP3 inflammasome activation. To this end, we depleted *Ncoa6* using siRNA in immortalized BMDMs carrying a gain-of-function mutation in the *Nlrp3* gene (*Nlrp3*^R258W^) [37]. As previously reported [34], the culture supernatants of *Nlrp3*^R258W^ BMDMs contained greater amounts of mature IL-1β and active caspase-1 (p20) than those of control BMDMs upon stimulation with LPS and/or LPS + ATP, confirming the mutation-induced hyperactivation of the NLRP3 inflammasome (Fig. 4H, I). Most strikingly, *Ncoa6* siRNA markedly suppressed the secretion of IL-1β and active caspase-1 (p20) in *Nlrp3*^R258W^ BMDMs in the presence of LPS alone (Fig. 4I, J). Moreover, the increased ASC oligomerization observed in LPS-stimulated *Nlrp3*^R258W^ BMDMs was markedly impaired by *Ncoa6* siRNA compared to control siRNA (Fig. 4K). This reversal of NLRP3 hyperactivation by the knockdown of *Ncoa6* further indicates that NCOA6 is an essential regulator of NLRP3 inflammasome activation in macrophages.

In summary, NCOA6 has robust effects on NLRP3 inflammasome activity through interaction with the Walker A and B motifs of the NLRP3 NACHT domain following cytoplasmic translocation in activated monocytes/macrophages.

### NCOA6 deficiency alleviates NLRP3-dependent inflammatory diseases in mice

**Fig. 5:**
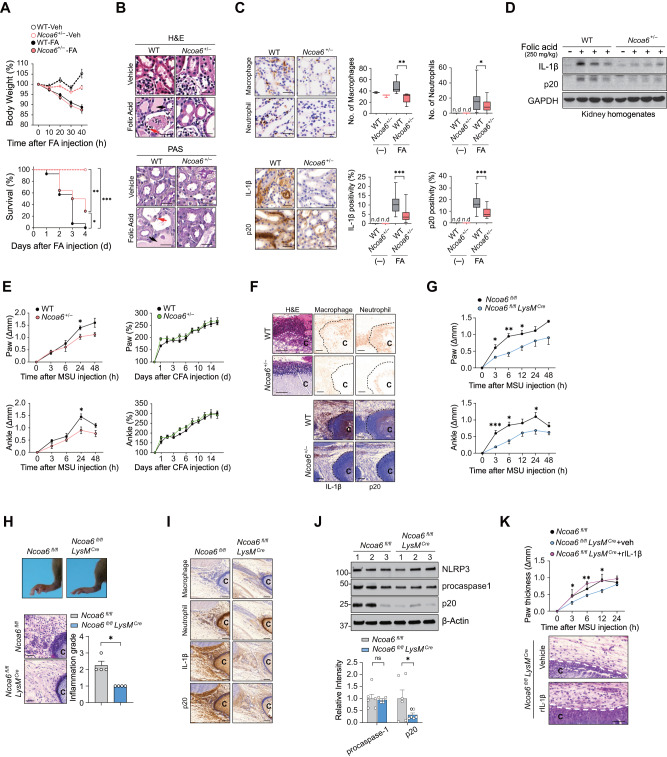
Amelioration of acute tubular necrosis and MSU crystal-induced arthritis in mice by *Ncoa6* deficiency. **A**–**D** Folic acid (FA) or vehicle (Veh) was injected into the peritoneal cavity of WT and *Ncoa6*^*+*/–^ mice. Kidney samples were obtained 36 h after FA injection. The body weight (**A** upper panel) and survival (**A** lower panel) of WT mice (n = 6 for Veh, *n* = 16 for FA) and *Ncoa6*^*+/−*^ mice (*n* = 5 for Veh, n = 14 for FA) were monitored for the indicated duration. Representative H&E (**B** upper panel) and periodic acid-Schiff images (**B** lower panel) of the kidney; the black arrows indicate cast formation, and the red arrows indicate tubular necrosis. Immunohistochemistry of kidney tissues (**C**) using anti-F4/80 Abs (for macrophages), anti-NIMP-14 Abs (for neutrophils), anti-mature IL-1β (p17) Abs, and anti-active caspase-1 (p20) Abs. The number of infiltrated cells in the corticomedullary junction was counted in randomly selected images (40x magnification) on a slide scanner (**C** bar graphs in the upper panel). IL-1β and active caspase-1 levels were assessed based on the ratio of the stained area per field using ImageJ software (**C** bar graphs in the lower panel). For (**C**), at least five fields were counted per slide. n.d.=not detected. Kidney homogenates were immunoblotted with Abs for mature IL-1β, active caspase-1, and GAPDH (**D**). MSU crystals (**E** left panel) or CFA (**E** right panel) were injected into the footpads of WT and *Ncoa6*^*+*/–^ mice. Paw and ankle edema was monitored for the indicated duration (**E**). Representative H&E and immunohistochemical staining of MSU-injected joint tissues using Abs for F4/80, NIMP-14, mature IL-1β, and active caspase-1 (**F**). **G–J** *Ncoa6*^*fl/fl*^ (*n* = 8) and *Ncoa6*^*fl/fl*^*LysM*^*CRE*^ (*n* = 8) mice were subcutaneously treated with MSU crystals. Paw and ankle edema was monitored for 2 days (**G**). Representative macroscopic images of hind limbs (**H** upper panel) and H&E-stained images of footpads (**H** lower panel). The bar graph in the lower panel of **H** shows the inflammation grade of the affected joints. Representative immunohistochemistry images (**I**). Representative immunoblotting images (**J** upper panel); the bar graph shows the mean intensity of caspase-1 (**J** lower panel), which was summed from two blot images. Each dot represents an individual mouse. **K** IL-1β-induced loss of the decrease in arthritis severity in *Ncoa6*-KO mice. Recombinant IL-1β or PBS was subcutaneously injected around crystals 1 h and 6 h after administering MSU to *Ncoa6*^*fl/fl*^ (*n* = 8) and *Ncoa6*^*fl/fl*^*LysM*^*CRE*^ mice (*n* = 8). Paw and ankle edema was monitored for 24 h (**K** top panel): representative images of H&E staining (**K** bottom panel). Each “C” in panels (**F**, **H**, **I**, **K**) indicates an MSU crystal. Scale bars = 50 μm for (**F**, **I**); 25 μm for (**B**, **C**, **H**); and 20 μm for (**K**). *P < 0.05, **P < 0.01, and ***P < 0.001 by log rank *t* test (**A**), unpaired two-tailed *t* test (**C**), and two-way ANOVA corrected for multiple comparisons (**E**, **G**, **J**, **K**)

Our in vitro findings strongly suggest that NCOA6 is required for the progression of NLRP3-dependent inflammatory diseases, although little is known regarding the role of NCOA6 in these diseases. Therefore, we investigated the in vivo role of NCOA6 in FA-induced acute tubular necrosis (ATN), a mouse model of NLRP3-dependent inflammatory disease in which IL-1β is released in response to dying cells, subsequently causing tubular damage in the kidney, such as tubular necrosis, cast formation, and tubular dilatation [38]. The results showed that no difference in body weight loss was detected between *Ncoa6*-haploinsufficient (*Ncoa6*^*+/−*^) mice and wild-type (WT) littermates (*Ncoa6*^*+/+*^) (Fig. 5A); *Ncoa6*^*+/−*^ mice were used because *Ncoa6*^*−/−*^ is embryonic lethal in mice [7]. However, *Ncoa6*^*+/−*^ mice exhibited an increased survival rate compared to that of WT mice after FA injection (Fig. 5A, bottom panel). Histopathological severity, as assessed by the extent of tubular necrosis, cast formation, and tubular dilatation, was also substantially reduced in *Ncoa6*^*+/−*^ mice compared to WT mice (Fig. 5B). In addition, the interstitial infiltration of macrophages and neutrophils was significantly decreased in *Ncoa6*^*+/−*^ mice with FA-induced ATN (Fig. 5C). Moreover, the production of IL-1β and active caspase-1 (p20), representative of NLRP3 inflammasome activity, was markedly hampered by *Ncoa6* haploinsufficiency (Fig. 5C, D). These observations suggest that *Ncoa6* deficiency prevented the development of FA-induced ATN, possibly by inhibiting IL-1β and caspase-1 production.

To further probe the pathological relevance of NCOA6, we established an in vivo model of gouty arthritis in mice (MSU crystal-induced arthritis), which is another representative model of NLRP3-dependent inflammatory disease [39]. As in FA-induced ATN, the severity of MSU crystal-induced arthritis was significantly diminished in *Ncoa6*^*+/−*^ mice 24 h after MSU injection, as assessed by the Δ thickness of the paw and ankle, which was decreased in the *Ncoa6*^*+/−*^ mice (Fig. 5E). In sharp contrast, mice with and without complete Freund’s adjuvant (CFA)-induced arthritis, which is not NLRP3 inflammasome-specific [40], showed no such difference in the thickness of the paw and ankle (Fig. 5E), indicating that NCOA6 may specifically contribute to gouty arthritis in vivo. Consistently, the infiltration of immune cells in the vicinity of MSU crystals was profoundly repressed by *Ncoa6* deficiency, as determined by immunostaining of the joints, which revealed a marked reduction in the number of anti-NIMP-14^+^ (for neutrophils) and F4/80^+^ cells (for macrophages) in the joints of *Ncoa6*^*+/−*^ mice (Fig. 5F). Moreover, IL-1β and active caspase-1 (p20) expression was less prominent in infiltrated cells in the joints of *Ncoa6*^*+/−*^ mice than in those of WT mice (Fig. 5F), which supports the idea that NCOA6 controls NLRP3 inflammasome activity.

To assess whether myeloid cells in particular were involved in the observed role of NCOA6 in NLRP3-dependent inflammatory diseases, we generated conditional KO mice lacking *Ncoa6* specifically in myeloid cells using the *Cre/loxP* system (Fig. S6A, B). As expected, in *Ncoa6*^*fl/fl*^*LysM*^*CRE*^ mice, *Ncoa6* mRNA levels were markedly reduced in BMDMs only, not in other cell types, such as T and B lymphocytes, confirming the specific depletion of *Ncoa6* in myeloid cells (Fig. S6C). Unfortunately, specific Abs for mouse NCOA6 are not commercially available, and thus, mouse NCOA6 protein levels could not be determined. When *Ncoa6*^*fl/fl*^*LysM*^*CRE*^ mice were subjected to MSU crystal-induced arthritis, they exhibited substantial attenuation of joint swelling compared to that of *Ncoa6*^*fl/fl*^ mice as early as 3 h after MSU injection (Fig. 5G), and this response was more pronounced than that in Ncoa6-haploinsufficient mice (Fig. 5E, G). The difference in inflammatory cell infiltration was in concordance with this phenotype; fewer NIMP-R14^+^ and F4/80^+^ cells were near the MSU crystals in *Ncoa6*^*fl/fl*^*LysM*^*CRE*^ mice than in *Ncoa6*^*fl/fl*^ mice (Fig. 5H, I). In parallel, IL-1β and active caspase-1 expression in joint tissues was dramatically downregulated in *Ncoa6*^*fl/fl*^*LysM*^*CRE*^ mice compared with *Ncoa6*^*fl/fl*^ mice (Fig. 5I, J). These decreases in arthritic severity and inflammatory cell infiltration were almost completely reversed by the intra-articular injection of recombinant IL-1β into the affected joints (Fig. 5K and Fig. S14), demonstrating the decisive role of the myeloid NCOA6–IL-1β axis in NLRP3-dependent gouty arthritis.

Consistent with the in vitro data, these in vivo data suggest that NCOA6 is necessary for NLRP3-dependent inflammatory diseases, including FA-induced ATN and MSU crystal-induced arthritis.

### NCOA6 is upregulated in NLRP3-dependent inflammatory diseases in humans

**Fig. 6:**
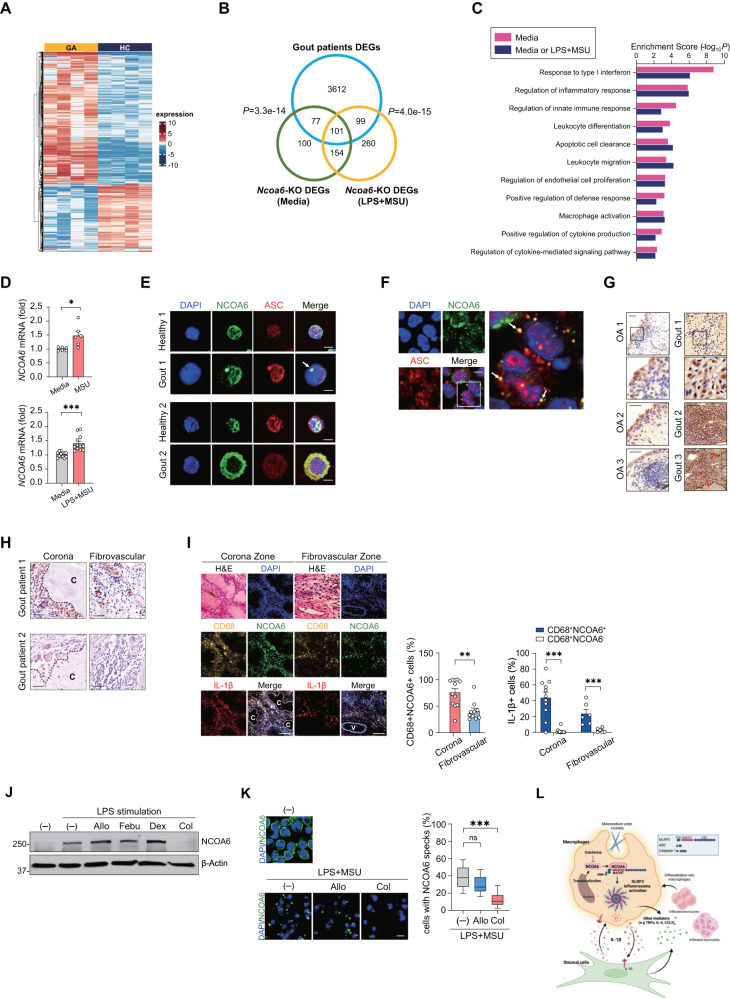
Pathological and clinical importance of NCOA6 in patients with gout arthritis. **A** Heatmap of 3894 DEGs (|log_2_ fold change| > 1) in synovial macrophages from patients with gouty arthritis (GA, *n* = 4) compared to CD14^+^ peripheral blood monocytes of healthy controls (HC, *n* = 4). **B** Venn diagram showing the overlap of the three sets of DEGs generated by the comparisons of gout macrophages versus healthy macrophages (blue), *Ncoa6*-KO BMDMs versus unstimulated WT BMDMs (green), and *Ncoa6*-KO BMDMs versus WT BMDMs stimulated with LPS plus MSU (yellow). *P* values were computed by chi-squared test. **C** GOBP terms related to inflammasome activation that were enriched in the overlapping genes (178 genes for medium alone and 277 genes for medium or LPS + MSU) between DEGs in *Ncoa6*-KO BMDMs and DEGs in gout macrophages. **D** qRT–PCR analysis of *NCOA6* mRNA levels in MSU- or LPS + MSU-stimulated human CD14^+^ monocytes^.^ **E**, **F** Representative immunofluorescence images of NCOA6 and ASC in CD14^+^ monocytes^,^ which were isolated from the peripheral blood of healthy controls and joint fluid of patients with gouty arthritis (**E**) or CPPD (**F**). Nuclei were stained with DAPI. **G** Immunohistochemistry analysis of NCOA6 in synovial tissues from osteoarthritis patients or gout arthritis patients; the red arrows indicate crystal deposits. **H** Images of immunohistochemical staining of NCOA6 in the corona and fibrovascular zones of the synovium of gout arthritis patients. C = MSU crystal. **I** Triple immunofluorescence staining for NCOA6, CD68 (macrophages), and IL-1β in the gouty arthritis synovium. Nuclei were stained with DAPI. The percentage of CD68^+^NCOA6^+^ cells (middle panel) and the ratios of IL-1β^+^ cells among CD68^+^NCOA6^+^ cells or CD68^+^NCOA6^–^ cells (right panel) were calculated from at least three fields per slide. Each dot represents one field. C = MSU crystal, V = blood vessel. **J** THP-1 cells were stimulated with LPS (10 ng ml^−1^) for 48 h in the presence of the indicated drugs. Cell lysates were immunoblotted with antibodies against NCOA6 and β-actin. **K** Differentiated THP-1 cells were primed with LPS (100 ng ml^−1^) for 4 h, followed by exposure to MSU crystals in the presence of allopurinol and colchicine. Representative immunofluorescence images are shown in the left panel. The ratio of cells with NCOA6 specks was calculated by randomly counting at least 50 cells per field (5 fields/group) (right panel). **L** Hypothetical model of the NCOA6-induced activation of the NLRP3 inflammasome in gout arthritis. Following the deposition of MSU crystals, monocytes and macrophages migrate to these deposits and attempt to phagocytize them. In macrophages stimulated by MSU and/or other proinflammatory stimuli, NCOA6 can translocate from the nucleus into the cytoplasm. Cytoplasmic NCOA6 incorporates into the NLRP3 inflammasome by binding the NACHT domain of NLRP3 and then activates the NLRP3 inflammasome and induces ASC oligomerization. As a result, activated macrophages secrete the mature form of IL-1β, which in turn may trigger the production of other inflammatory mediators, including TNFα, IL-6, CCL2, and prostaglandins, by stromal cells; these events lead to the recruitment of more monocytes and neutrophils to the inflamed sites embedded with MSU crystals, thus generating the vicious cycle of gouty inflammation. Treatment with colchicine might block this self-perpetuating cycle by repressing NCOA6 expression. Scale bars = 20 μm for (**E**, **F**); 50 μm for (**G**, **H**, **K**); and 100 μm for (**I**). *P < 0.05, **P < 0.01, and ***P < 0.001 by paired two-tailed *t* test (**D**), unpaired two-tailed t test (**I**) and one-way ANOVA corrected for multiple comparisons (**K**)

Most studies on the association of NCOA6 with disease have been conducted in *Ncoa6*-deficient mice, with few studies in human systems. Therefore, it is unclear whether NCOA6 is implicated in the development of disease in humans. To ascertain the clinical relevance of NCOA6 as a critical regulator of the NLRP3 inflammasome, we profiled global gene expression in synovial fluid macrophages freshly isolated from gouty arthritis patients (n = 4) in comparison with peripheral blood monocytes from healthy donors (*n* = 4). We identified 3889 DEGs (2601 upregulated and 1288 downregulated) in gout macrophages (Fig. 6A and Table S3) and found numerous shared genes between these DEGs and those in *Ncoa6-*KO macrophages (from *Ncoa6*^*fl/fl*^ *LysM*^*CRE*^ mice) cultured with medium alone (no. of shared DEGs=178) or stimulated with LPS + MSU (no. of shared DEGs=200) (Fig. 6B). Functional enrichment analysis of the 277 shared DEGs demonstrated that the response to type I IFN, regulation of the inflammatory response, innate immune response, leukocyte differentiation, and leukocyte migration were significantly enriched (Fig. 6C and Table S4), which supports the hypothesis that NCOA6 controls innate and inflammatory responses, including macrophage activation, migration, and differentiation, in gouty arthritis, an NLRP3-dependent inflammatory disorder in humans [41]. Moreover, the 33 DEGs that were downregulated in both medium- and LPS + MSU-stimulated *Ncoa6*-KO BMDMs was significantly upregulated in gout macrophages, as determined by GSEA (Fig. S15A). The GOBP terms enriched in the 33 DEGs were mostly immune-related cellular processes (Fig. S15B, C, and Table S5). Overall, the transcriptome data suggest that NCOA6 is a key regulator dictating the pathological activation of macrophages in gouty arthritis.

Consistent with RNA sequencing data in gout macrophages, *NCOA6* expression in peripheral human monocytes of healthy donors was upregulated in response to the administration of MSU crystals (Fig. 6D), which are frequently detected inside gout macrophages [42]. Moreover, in synovial fluid macrophages from patients with gout and calcium pyrophosphate deposition (CPPD), both of which are NLRP3 inflammasome-dependent diseases [30, 43], NCOA6 was detected in cytoplasmic specks (single and multiple) that colocalized with ASC, as determined by immunofluorescence staining (Fig. 6E, F), which is quite similar to the findings observed in PMA-primed THP-1 cells (Fig. 2E, F). In contrast, in peripheral monocytes from healthy controls, NCOA6 localization was entirely limited to the nucleus (Fig. 6E). Interestingly, the cytoplasmic colocalization of NCOA6 with ASC was also noted in neutrophils in the synovial fluid of gout patients, another type of innate immune cell critically involved in gouty arthritis [44], suggesting that this colocalization is not unique to macrophages (Fig. S16).

Moreover, immunohistochemical staining revealed that NCOA6 expression was upregulated in the synovial tissues of gout patients compared to those of osteoarthritis (OA) patients, who served as a control (Fig. 6G). The gout synovium consists of three zones, including the central crystalline core, corona zone, and fibrovascular zone [45]. CD68^+^ macrophages are infiltrated mainly in the corona zone adjacent to the crystalline core but are rarely in the fibrovascular zone [46]. In particular, IL-1β-expressing macrophages, which are reported to be more pathogenic [47], are more frequently found in the corona zone. In this study, we found that there was a spatial difference in NCOA6 expression in the synovium, with a higher number and frequency of NCOA6^+^ macrophages in the corona zone neighboring the tophus than in the fibrovascular zone far from the tophus (Fig. 6H, I). Triple immunofluorescence staining revealed that IL-1β positivity was strikingly higher in NCOA6^+^CD68^+^ macrophages than in NCOA6^−^CD68^+^ macrophages (Fig. 6I). These observations demonstrate that macrophages with NCOA6 expression are concentrated in the inflammatory area of the gout synovium, supporting the notion that NCOA6 upregulates IL-1β expression as an NLRP3 inflammasome activator.

Taken together, the in vitro, in vivo, human pathology, and transcriptome profiling results indicate that NCOA6 may be an attractive target for NLRP3-dependent inflammatory diseases. Therefore, we finally wondered whether NCOA6 is involved in the mechanism of approved anti-gout drugs, including allopurinol, febuxostat, dexamethasone, and colchicine, all of which have been commonly prescribed for gout patients [48]. We observed that allopurinol, febuxostat, and dexamethasone failed to significantly suppress NCOA6 expression in THP-1 cells primed with LPS (Fig. 6J), whereas colchicine treatment completely suppressed NCOA6 levels under the same conditions (Fig. 6J). Furthermore, NCOA6 speck formation in THP-1 cells stimulated with LPS + MSU was markedly decreased by colchicine (Fig. 6K), indicating that the anti-gout activity of colchicine might occur via the blockade of NCOA6 expression and/or speck formation.

Figure 6L illustrates a hypothetical model of how NCOA6 in macrophages activates the NLRP3 inflammasome complex and contributes to the progression of gouty arthritis.

## Discussion

Our preliminary global interactome analysis between coactivators and transcription factors revealed nuclear receptor coactivator 6 (NCOA6) as a key regulator involved in the innate immune response. Hence, the primary goal of our study was to address simple questions of whether NCOA6, a coactivator, plays a role in innate immunity and inflammation. Unexpectedly, the results of this study substantially advanced our understanding of immunology and cell biology. First, it was unclear whether nuclear coactivators escape from the nucleus, playing additional functional roles. Our data clearly demonstrate that nuclear NCOA6, beyond its canonical function of nuclear coactivation, translocates to the cytoplasm and then acquires a new function of NLRP3 activation. Second, we determined that NCOA6 is a novel regulator of the NLRP3 inflammasome complex. Mechanistically, cytoplasmic NCOA6 interacts with the NACHT domain of NLRP3 at Walker A and B motifs and then regulates its activity. Finally, with bioinformatics, in vitro, and in vivo data on the direct role of NCOA6 in the innate immune response and diseases, we suggest NCOA6 as a new therapeutic target for NLRP3-dependent diseases, including gouty arthritis and acute tubular necrosis.

In eukaryotic cells, molecules have continuously acquired more than one function. One way of achieving multifunctionality for a given molecule is to ensure topological diversity within the cell [49]. In such cases, the molecule can acquire a new function by changing its subcellular localization. For example, FOS is a TF in the nucleus but a phospholipid synthesis activator at the endoplasmic reticulum (ER) membrane [50]. Nuclear receptors also contribute to the nongenomic regulation of cellular functions, including apoptosis, autophagy, and inflammasomes, after leaving the nucleus [51, 52]. As a coactivator, NCOA6 is mainly located in the nucleus. Although nuclear NCOA6 has been intensively studied, the cytoplasmic and subcellular localization of NCOA6, which is potentially important for its function, remains poorly understood. One of the striking findings of this study is that NCOA6 undergoes nucleocytoplasmic redistribution to perform new functions in innate immunity and NLRP3 activation, representing a new example of the multifunctionality of nuclear coactivators. However, it is unclear at this point how NCOA6 translocates from the nucleus to the cytoplasm. In a previous study, a nuclear localization signal (NLS) in *NCOA6* was mapped to the C-terminal sequence coded by the last four exons [18]. We suspect that the putative NLS in the NCOA6 gene plays a role in determining its cellular distribution, similar to the role of the NLS in ASC, another inflammasome component, in macrophages [22].

NCOA6 interacts with a variety of TFs and nuclear receptors [8]. Surprisingly, we found that NCOA6 acts as a signal regulator in the cytoplasm. Indeed, as early as 24 h after stimulation with PMA, most NCOA6-positive THP-1 cells were localized in the nucleus and was rarely detected in the cytoplasm (data not shown), suggesting that cytoplasmic NCOA6 plays a predominant role in activated monocytes/macrophages but not in resting cells, presumably losing its coactivator function. Our data also show that cytoplasmic NCOA6 was expressed prior to NLRP3 and was associated with NLRP3 with the Walker A and B motifs of the NLRP3 NACHT domain, which is responsible for ATPase-dependent ASC oligomerization [53], without directly interacting with ASC. Moreover, *Ncoa6* deficiency suppressed the two-step mechanism of inflammasome activation, particularly Signal 2 (nontranscriptional NLRP3 inflammasome activation), and even reversed the NLRP3 hyperactivation caused by the *Nlrp3*^R258W^ gain-of-function mutation, indicating that NCOA6 is indispensable for inflammasome activity as a strong upstream regulator of the NLRP3 complex. In this context, targeting the NLRP3 ATP hydrolysis motifs (Walker A and B) may hold promise in alleviating NCOA6-induced NLRP3 hyperactivation and could be a novel treatment strategy for inflammatory diseases dependent on NLRP3, including gouty arthritis.

Infiltrated monocytes and resident macrophages play pivotal roles in NLRP3 inflammasome-dependent diseases [54]. These cells are activated by DAMPs at the initial stage of a disease, produce proinflammatory cytokines, including IL-1β, and then recruit neutrophils to inflamed sites to amplify IL-1β production [55]. Our work underscores the importance of NCOA6 in the pathogenesis of macrophage-dependent inflammatory diseases. Using loss-of-function mouse models, we clearly showed that NCOA6, particularly myeloid NCOA6, has critical functions in promoting NLRP3 inflammasome-mediated inflammatory diseases, including FA-induced ATN and MSU crystal-induced arthritis. Given the molecular interaction of NCOA6 with TFs (Fig. S1), such proinflammatory responses induced by cytoplasmic NCOA6 (for Signal 2) could be further enhanced together with the coactivation of inflammasome-related TFs mediated by nuclear NCOA6 (for Signal 1). Interestingly, our integrated transcriptome analysis suggests that macrophage NCOA6, in addition to innate immunity, could be involved in diverse cellular processes, such as cell differentiation and cytokine responses. Further investigation is warranted to verify the role of macrophage NCOA6 in these processes.

Gouty arthritis is one of the most common rheumatic diseases worldwide where MSU crystals elicit robust inflammation of the affected joints by activating the innate immune system, specifically by inducing secretion of a large amount of IL-1β from macrophages and neutrophils via NLRP3 inflammasome activation [56]. To our knowledge, global transcriptomics analysis of gouty macrophages has not been performed. In the present study, the transcriptome, qRT‒PCR, immunofluorescence, and immunohistochemistry data using macrophages and synovia of gout patients suggest that NCOA6 is a key regulator of gouty arthritis, confirming our major findings in the human system. Notably, among approved anti-gout drugs, colchicine completely abrogated NCOA6 expression and speck formation in activated macrophages, suggesting that colchicine inhibits NLRP3 inflammasome activity by targeting NCOA6. In fact, a number of proinflammatory genes, including GM-CSF, TNF, and IL6, can be transcriptionally downregulated by colchicine [57]. Our data corroborate earlier reports [57, 58] and reveal a new molecular mechanism by which colchicine exerts its anti-gout and anti-inflammasome activities [30].

In summary, we showed that NCOA6 was expressed in monocytes and macrophages and that its level was increased under proinflammatory conditions in both human and mouse systems. Unexpectedly, nuclear NCOA6 was found to translocate to the cytoplasm in activated monocytes and then become incorporated into the inflammasome with NLRP3 and ASC, forming cytoplasmic specks. NCOA6 was associated with ATP hydrolysis motifs in the NACHT domain of NLRP3, promoting the oligomerization of NLRP3 and ASC and thereby enhancing the production of IL-1β and active caspase-1. *Ncoa6* deficiency blocked the hyperactivation of NLRP3 and ASC oligomerization in *Nlrp3*^R258W^ BMDMs. Genetic ablation of *Ncoa6* substantially attenuated the severity of FA-induced ATN and MSU crystal-induced arthritis in mice and decreased IL-1β and active caspase-1 levels. The suppression of MSU crystal-induced arthritis was reproduced by the myeloid-specific depletion of *Ncoa6*. Consistent with these findings, the NCOA6 signature was significantly enriched in gouty macrophages. Moreover, NCOA6 expression was elevated in the synovial macrophages of patients with gouty arthritis, correlating with IL-1β expression.

Conclusively, NCOA6, in addition to its classical role in transcriptional coactivation, plays a newly discovered role in innate immunity upon redistribution to the cytoplasm, highlighting the functional diversity of the nuclear coactivators that potentially undergo changes in subcellular localization. Moreover, our findings provide novel insight into the NCOA6-directed molecular mechanism(s) of NLRP3 inflammasome activation, emphasizing the importance of the NACHT domain in this process. We anticipate that NCOA6-suppressing agents, such as colchicine, will alleviate the feed-forward loop of NLRP3 activation (Fig. 6L), making them attractive candidates for the treatment of various NLRP3 inflammasome-dependent disorders.

## Materials and methods

### Mice

*Ncoa6*^+/−^ mice on a C57BL/6 genetic background were generated as previously described [59]. Age- and sex-matched wild-type (WT) littermates (C57BL/6) were used as controls. Mice with conditional *Ncoa6* knockout (KO) specifically in myeloid cells (*Ncoa6*^*fl/fl*^ × *LysM*^*Cre*^) were created using the *loxP/Cre* system. *Ncoa6*^*fl/fl*^ mice served as control mice. The animal genotype was confirmed by PCR analysis using the following primers: *Flox* forward, 5′-GGCTCATTTTCTAGCCCATGA-3′ *Flox* reverse, 5′-AGGACCAGCTCCTTGACCACC-3′; *Cre* forward, 5′-CCCAGAAATGCCAGATTACG-3′; and *Cre* reverse, 5′-CTTGGGCTGCCAGAATTTCTC-3′. All mice were maintained under specific pathogen-free conditions. The experimental procedures were approved by the Institutional Animal Care and Use Committee of the Catholic University of Korea (IACUC, approval number: CUMC-2020-0332-01).

### Reagents

Phorbol 12-myristate 13-acetate (PMA, P8139), lipopolysaccharides from *E. coli* O111:B4 (L2630), adenosine triphosphate (ATP, A2383), potassium chloride (KCl, P5405) MG132 (C2211), folic acid (F876), allopurinol (A8003), and dexamethasone (D2915) were purchased from Sigma‒Aldrich (St. Louis, MO, USA). Recombinant human TNFα (210-TA), human M-CSF (216-MC), mouse IL-1β (401-ML), and mouse TNFα (410-MT) were purchased from R&D Systems (Minneapolis, MN, USA). Monosodium urate crystals (MSU crystals, tlrl-msu), *Salmonella* flagellin (FLS-ST, tlrl-stfla), and poly (dA:dT) (tlrl-patn) were obtained from InvivoGen (San Diego, CA, USA). Complete Freund’s adjuvant (CFA, 7001) and incomplete Freund’s adjuvant (IFA, 7002) were obtained from Chondrex (Redmond, WA, USA). Colchicine (AG-CN2-0048) was purchased from AdipoGen (San Diego, CA, USA). Recombinant mouse M-CSF (315-02) was purchased from PeproTech (Rocky Hill, NJ, USA). Recombinant human IL-1β (RIL1B1) and disuccinimidyl suberate (DSS, 21655) were purchased from Thermo Fisher Scientific (Cleveland, OH, USA). Human *NCOA6* siRNA (sc-61401), control siRNA (sc-37007), and febuxostat (144060-53-7) were purchased from Santa Cruz Biotechnology (Dallas, TX, USA). ON-TARGETplus mouse *Ncoa6* siRNA (L-041129-00-0005) and ON-TARGETplus nontargeting control siRNA (D-001810-10-05) were purchased from Dharmacon (Lafayette, CO, USA).

### Reconstruction of the molecular network of immune response-related TFs and coactivators

The gene lists of immune response-related TFs and nuclear receptor coactivators were obtained from the Gene Ontology database. Briefly, we first searched for the shared genes between the “immune response” (GO:0006955) and “DNA-binding transcription factor activity, RNA polymerase II-specific” (GO:0000981) biological process gene sets defined by Gene Ontology (GO) and considered the genes ‘immune response-related TFs’. We next selected the genes having “nuclear receptor coactivator activity” defined by the Gene Ontology molecular function (GOMF, GO:0030374) dataset. After obtaining the protein‒protein interaction (PPI) data of the selected TFs and nuclear receptor coactivators using the STRING database (5), we visualized the PPI results as a network using Cytoscape (ver. 3.8.0). The interaction degrees of each protein were analyzed by the Network Analyzer application in Cytoscape.

### Cell culture

THP-1 cells (a human myelomonocytic cell line) were maintained in RPMI 1640 medium containing 10% fetal bovine serum (FBS), 1% antibiotics/antimycotics, and 0.05 mM 2-mercaptoethanol. THP-1 cells were differentiated into macrophage-like cells by treatment with 10 ng mL^−1^ PMA for 3 days. HEK293T cells were maintained in DMEM containing 10% FBS and 1% antibiotics/antimycotics. Human monocytes were freshly isolated from the peripheral blood of healthy individuals using a monocyte isolation kit II (Miltenyi Biotech, Surrey, UK). Human monocyte-derived macrophages were generated by incubating the isolated CD14^+^ monocytes in complete RPMI 1640 medium supplemented with 20 ng mL^−1^ human M-CSF for 3 days, as previously described [60]. To generate BMDMs, bone marrow cells were harvested from the tibia and femur of C57BL/6, *Ncoa6*^+/−^, *Ncoa6*^*fl/fl*^, and *Ncoa6*^*fl/fl*^ × *LysM*^*Cre*^ mice and then differentiated by culture in RPMI 1640 medium containing murine M-CSF (20 ng mL^−1^) for 6 days, as previously described [60]. HEK293T cells were cultured in DMEM supplemented with 10% FBS (Welgene, Korea, LM001-09) and 1% antibiotics/antimycotics. Immortalized *Nlrp3*^R258W^ BMDMs, kindly provided by Professor Yuan He at Wayne State University School of Medicine, were cultured in IMDM supplemented with 10% FBS (Gibco, Grand Island, NY, USA), 1× nonessential amino acids, 1× sodium pyruvate, and 1% antibiotics/antimycotics.

### Flow cytometry

Peripheral blood mononuclear cells from healthy individuals were stained with PE-labeled CD14 and APC-labeled CD16 Abs for 30 min at 4 °C in the dark. For intracellular staining, the cells were permeabilized (eBioscience, San Diego, CA, USA) and stained with Alexa 488-conjugated anti-NCOA6 Abs for 30 min at room temperature in the dark. The Alexa 488-conjugated IgG isotype Ab (Santa Cruz Biotechnology) was used as a control. The stained cells were analyzed using a FACS Canto II system (BD Biosciences).

### Immunoblotting

For immunoblotting, cells were lysed in RIPA buffer (50 mM Tris-Cl [pH 8.0], 150 mM NaCl, 0.1% sodium lauryl sulfate [SDS], 0.5% sodium deoxycholate, 1% NP40, and a protease inhibitor cocktail) or 2X SDS loading buffer. The cell lysates were then centrifuged at 13,000 rpm for 30 min. The supernatant was collected, and the protein concentration was determined by a Bradford protein assay (Bio-Rad). The polypeptides were separated on 4–20% SDS–PAGE gradient gels and then transferred to a polyvinylidene difluoride (PVDF) membrane (GE Healthcare, Munich, Germany). Primary Abs against the following proteins were used: human/mouse NLRP3 (1:1000, AP-20B-0014, AdipoGen), human NCOA6 (1:1000, A300-411A, Bethyl Laboratories), human/mouse ASC (1:1000, sc-514414, Santa Cruz Biotechnology; AG-25B-0006, AdipoGen), IL-1β (1:500, AF-401-NA, R&D Systems), mouse cleaved caspase-1 (1:1000, AG-20B-0042, AdipoGen), FLAG (1:1000, sc-166355, Santa Cruz Biotechnology), Myc (1:1000, ab13836, Abcam), human/mouse NEK7 (1:1000, ab133514, Abcam), GAPDH (1:1000, sc-25778, Santa Cruz Biotechnology), and β-actin (1:1000, sc-47778, Santa Cruz Biotechnology). The membranes were incubated with primary Abs, followed by incubation with HRP-conjugated anti-rabbit IgG (1:100000, 31460, Thermo Fisher Scientific) or HRP-conjugated anti-mouse IgG (1:5000, 31430, Thermo Fisher Scientific). The signals were detected with SuperSignal Chemiluminescence Reagent (Thermo Fisher Scientific) and ImmunoCruz ECL (Santa Cruz Biotechnology).

### Immunohistochemistry

For immunohistochemistry, mouse kidney tissues, mouse paw tissues, and human synovial tissues were fixed in 4% paraformaldehyde, embedded in paraffin, and sectioned. Following deparaffinization, antigen retrieval was performed with sodium citrate buffer. Endogenous peroxidase activity was quenched with hydrogen peroxide. After blocking with 10% normal donkey serum, the sections were stained with Abs against neutrophils (sc59338, Santa Cruz Biotechnology), macrophages (sc101447, Santa Cruz Biotechnology), IL-1β (12242, Cell Signaling Technology, Beverly, MA, USA), caspase-1 (AG-20B-0042, AdipoGen), and NCOA6 (A300-411A, Bethyl Laboratories). Images were taken with a Pannoramic MIDI slide scanner.

### Immunocytochemistry

Human monocyte-derived macrophages or differentiated THP-1 cells were cultured on coverslips and stimulated with PMA, LPS, ATP, or LPS plus ATP. Cells were fixed in 4% paraformaldehyde and processed for indirect immunofluorescence staining using the following Abs: mouse anti-human NLRP3 (1:100, ab16097, Abcam, Cambridge, MA, UK), mouse anti-human ASC (1:50, sc-514414, Santa Cruz Biotechnology), rabbit anti-human NCOA6 (1:500, A300-411A, Bethyl Laboratories, Montgomery, TX, USA), mouse anti-vimentin (1:100, ab8987, Abcam), and appropriate secondary Abs, such as donkey anti-mouse IgG Cy3 (1:200, 715-166-151, Jackson laboratory, Bar Harbor, MA, USA) and donkey anti-rabbit IgG Alexa 488 (1:1000, A21206, Invitrogen). The Golgi, ER, and lysosomes were stained using CellLight^TM^ Reagents BacMam 2.0 (Thermo Fisher Scientific) according to the manufacturer’s instructions. The anti-NCOA6 Abs used in this study recognized the C-terminal end (amino acids 2000 to 2063) of human NCOA6 and thus are specific for only NCOA6α and NCOA6β out of four NCOA6 isoforms (α, β, γ, and δ), as these are the only isoforms that possess the recognized sequence [18]. Images were taken with a Zeiss LSM 810 microscope and contrast-enhanced using Zeiss ZEN microscope software.

### Nuclear and cytoplasmic fractionation

Cells were harvested and lysed with cytoplasmic lysis buffer (0.25% NP40, 10 mM Tris-HCl [pH 7.4], 10 mM NaCl, 3 mM MgCl_2_, 1 mM Na_3_VO_4_, 10 mM NaF, and protease inhibitor) for 4 min on ice. After centrifugation, the supernatant was collected to obtain the cytoplasmic fraction. The residual pellet was lysed with RIPA buffer and centrifuged for 30 min at 13,000 rpm; the supernatant served as the nuclear fraction. Each fractionated lysate was subjected to immunoblotting using Abs against NCOA6, Lamin B2 (1:200, sc-56147, Santa Cruz Biotechnology), and α-tubulin (1:1000, T5168, Sigma‒Aldrich).

### RNA sequencing

Mouse BMDMs from different *Ncoa6*^*fl/fl*^ or *Ncoa6*^*fl/fl*^*LysM*^*CRE*^ mice (*n* = 2 per group) were stimulated with medium only or primed with LPS (100 ng ml^−1^) for 4 h, followed by MSU (200 μg ml^−1^) stimulation for 2 h. Total RNA was extracted with TRIzol reagent according to the manufacturer’s instructions (Invitrogen). The RNA concentration was calculated by Quant-IT RiboGreen (Invitrogen, R11490). To assess the integrity of the total RNA, samples were run on a TapeStation RNA system (Agilent Technologies, Santa Clara, CA, USA, 5067–5576). Only high-quality RNA preparations with an RNA integrity number (RIN) greater than 7.0 were used for RNA library construction. RNA-seq libraries were prepared according to the TruSeq Stranded mRNA Sample Preparation Guide, Part #15031047 Rev. E (Illumina) and were then submitted for paired-end (2 × 100 bp) sequencing on an Illumina NovaSeq system (Illumina, Inc., San Diego, CA, USA) by Macrogen Incorporated (https://www.macrogen.com/).

CD14^+^ cells were isolated from the peripheral blood of healthy donors or synovial fluid of age- and sex-matched patients with gout arthritis who had needle-shaped MSU crystals with negative birefringence in leukocytes by polarized microscopy analysis (*n* = 4 per group). A total of 100 ng of total RNA was subjected to sequencing library construction using an Agilent SureSelect RNA Direct kit according to the manufacturer’s protocol. Indexed libraries were then submitted for paired-end (2 × 100 bp) sequencing on an Illumina NovaSeq system (Illumina, Inc., San Diego, CA, USA) by Macrogen Incorporated.

### Data preprocessing and normalization

The quality of the sequencing data was verified using MultiQC software [61]. Sequence alignment and quantification were performed using STAR (ver. 2.7.3a)–RSEM (ver. 1.3.3) pipeline [62, 63]. Reads overlapping exons annotated in the Genome Reference Consortium Mouse Build 38 (GRCm38, release number 101) and Genome Reference Consortium Human Build 38 (GRCh38, release number 105) were identified and excluded from further downstream analysis if they had fewer than 10 raw read counts across all the libraries. IGV and RSeQC were used to visualize read coverage of the reference gene sequence [64, 65].

### Identification of DEGs and functional enrichment analysis

The expected counts were imported by using the tximport package [66]. The DESeq2 R package was used to calculate normalized count data from RNA sequencing data by regularized log transformation and to conduct differential gene expression analyses [67]. For the SureSelect dataset, the edgeR package was used for preprocessing and TMM normalization [68]. DEGs in the macrophage datasets were analyzed using GEO2R and selected based on a false discovery rate (FDR) < 0.05 and |log2-fold change| > 1. Functional enrichment analysis of the DEGs was performed to identify significantly enriched GOBP terms using the clusterProfiler R package [69]. The enrichment score of each enriched pathway was calculated by converting its adjusted *P* value to -log_10_*P*. The fgsea R package was used to conduct GSEA [70].

### RNA extraction and quantitative real-time PCR

Total RNA was extracted from THP-1 monocytes, HEK293T cells, HELA cells, mouse BMDMs, human breast cancer cells (MDA-MB-231), and human CD14+ monocytes using TRIzol reagent according to the manufacturer’s instructions (Invitrogen, Carlsbad, CA, USA). cDNA was synthesized using reverse transcriptase (Takara, Tokyo, Japan). Quantitative real-time PCR (qRT–PCR) was performed in a CFX96 real-time thermal cycler (Bio-Rad, Richmond, CA, USA) using SYBR Green Supermix (Bio-Rad). Transcript levels were calculated relative to the GAPDH gene as an internal control using the –ΔΔCt method. The following primers were used for amplification: *Homo sapiens*: *NCOA6* (5′-AAAACGTGCCCAATTTGTTACAC-3′ and 5′-GAGAATCCCTAAATCCCGAAGC-3’), *IL1B* (5′-ATGATGGCTTATTACAGTGGCAA-3′ and 5′-GTCGGAGATTCGTAGCTGGA-3′), *NLRP3* (5′-ATCAGTATTGAGCACCAGCCATT-3′ and 5′-GAGTGTTGCCTCGCAGG-TAAAG-3′), *CASP1* (5′-GCACAAGACCTCTGACAGCA-3′ and 5′-TTGGGCAGTTCTT-GGTATTC-3’), and *GAPDH* (5’-AAGGTGAAGGTCGGAGTCAA-3′ and 5’-AATGAAGG-GGTCATTGATGG-3′); *Mus musculus*: *Ncoa6* (5′-GAAGAAACCGCCTCGGAAGA-3′ and 5′-CCTCTAGACCAGTTGGACGATTATCT-3′), *Il1b* (5′-GTGGCTGTGGAGAAGCT-GTG-3′ and 5′-GAAGGTCCACGGGAAAGACAC-3′), *Nlrp3* (5′-CGAGACCTCTGGGA-AAAAGCT-3′ and 5′-GCATACCATAGAGGAATGTGATGTACA-3′), *Pycard* (5′-GTCAC-AGAAGTGGACGGAGTG-3′ and 5′-CTCATCTTGTCTTGGCTGGTG-3′), *Casp1* (5′-CGTGGAGAGAAACAAGGAGTG-3′ and 5′-AATGAAAAGTGAGCCCCTGAC-3′), *Nek7* (5′-CCGTTACTCAGTTCCAGCCA-3′ and 5′-CTACCGGCACTCCATCCAAG-3′), and *Gapdh* (5′-AGGTCGGTGTGAACGGATTTG-3′ and 5′-TGTGACCATGTAGTTGAGGTC-A-3′). The PCR primers used for detecting human *NCOA6* splice variants were as follows: E9 (5′-AGAGTGACATATCTGCAGGC-3′), E11a (5′-GCAATCATGGTGGCATAGC-3′), E11b (5’-TTACCACTCAGTGGTAGGC-3’), E12 (5′-TCTGGTCTGGCAACAGAG-3′), E15 (5′-GTCCTGCTTGTTTACTTGGATTTTC-3′), and GAPDH (forward 5′-CCACCCTG-TTGCTGTAGCCAAA-3′ and reverse 5′-GTCAGTGGTGGACCTGACCT-3′).

### Enzyme-linked immunosorbent assay

Human primary monocytes and mouse BMDMs were stimulated as previously indicated. Cytokine concentrations in the culture supernatants were determined by ELISA kits for human IL-1β (DY201, R&D Systems), human TNFα (DY210, R&D Systems), human IL-6 (DY206, R&D Systems), human active caspase-1 (DCA100, R&D Systems), mouse IL-1β (DY401, R&D Systems), mouse TNFα (DY410, R&D Systems), mouse IL-6 (DY406, R&D Systems), and mouse active caspase-1 (NBP2–75014, Novus Biologics, Littleton, CO, USA) according to the manufacturer’s instructions.

### ASC oligomerization assay

Cells were lysed in cold buffer A (20 mM HEPES-KOH [pH 7.5], 10 mM KCl, 1.5 mM MgCl_2_, 1 mM EDTA, and 320 mM sucrose) and then sheared 30 times with a 22-gauge needle. Soluble cell lysates were centrifuged and resuspended in one volume of CHAPS buffer (20 mM HEPES-KOH [pH 7.5], 5 mM MgCl_2_, 0.5 mM EGTA, 0.1 mM PMSF, and 0.1% CHAPS). Cell lysates were centrifuged again, and the pellets were crosslinked in CHAPS buffer containing 4 mM disuccinimidyl suberate (21655, Thermo Fisher Scientific) for 30 min at room temperature. After adding 2× SDS loading buffer without β-mercaptoethanol, the cross-linked pellets were boiled for 5 min at 95 °C.

### Immunoprecipitation

Cells were lysed with IP lysis buffer (20 mM Tris-Cl [pH 7.5], 150 mM NaCl, 1% NP40, DTT, Na_3_VO_4_, PMSF, and protease inhibitors). Whole-cell lysates were precleared with 15 μl of Protein G agarose beads (Thermo Fisher Scientific) for 30 min at 4 °C and then incubated with 1 μg of the indicated Ab overnight. The resultant lysates were incubated with Protein G agarose beads for an additional 4 h. Immune complexes were washed with IP wash buffer and eluted with SDS sample loading buffer. Samples were boiled for 10 min at 95 °C.

### In situ proximity ligation assay (PLA)

PMA-stimulated THP-1 cells were cultured on an eight-well chamber slide. In the presence or absence of LPS + ATP, the cells were subjected to PLA according to the manufacturer’s instructions (DUO92102, Sigma‒Aldrich). Briefly, cells were fixed with 4% PFA, incubated with Duolink blocking solution for 1 h at 37 °C in an cell culture chamber, and then incubated again with primary antibodies, including the anti-NCOA6 antibody (1:100, Bethyl Laboratories) and anti-NLRP3 antibody (1:100, Adipogen), overnight at 4 °C. After washing with PBS twice for 5 min at room temperature, the cells were treated with PLA probes for 1 h at 37 °C. The cells were washed with PBS twice for 5 min at room temperature, and ligase was added to the cells for 30 min at 37 °C. After washing twice with PBS, the cells were incubated with polymerase for 100 min at 37 °C. Images were taken with a Zeiss LSM 900 microscope and analyzed with ImageJ software. The number of red fluorescent dots for each Z-stack was divided by the total number of cells labeled with DAPI per image.

### Plasmid construction and transfection

The pcDNA3-FLAG-NLRP3 full-length and NLRP3 domain (PYD, NACHT, and LRR) expression plasmids were provided by Eun-Kyeong Jo (Chungnam National University School of Medicine, South Korea) [71], and pcDNA3-Myc-ASC was purchased from Addgene (Cambridge, WA, USA). NLRP3 mutants at the Walker A and Walker B motifs (K232A, D305A, and E306A) [36] were generated by Macrogen Incorporated (Seoul, Korea). For gene overexpression, plasmids expressing FLAG-tagged NLRP3, FLAG-tagged NLRP3 domains, FLAG-tagged NLRP3 mutants or Myc-tagged ASC were transfected into HEK293T cells with FuGENE HD transfection reagent (Promega, Madison, WA, USA, E2311) for 24 h. For gene knockdown, siRNAs were transfected into human CD14^+^ monocytes and immortalized *Nlrp3*^R258W^ BMDMs using the Neon^TM^ transfection system (Life Technologies, Gaithersburg, MD, USA).

### Folic acid-induced acute tubular necrosis

Mice were intraperitoneally injected with folic acid (250 mg kg^−1^, Sigma‒Aldrich) dissolved in sodium bicarbonate (150 mM). The mice were monitored for survival and weighed at 0, 10, 20, 30, and 40 h after folic acid injection. At 36 h after the injection, the mice were anesthetized with Avertin (Sigma‒Aldrich) and subjected to whole-body perfusion with PBS and fixation with 4% paraformaldehyde. The kidneys were harvested, fixed with 4% paraformaldehyde, paraffin-embedded, and sectioned at a thickness of 4 μm. The sections were processed for hematoxylin and eosin (H&E) staining, periodic acid-Schiff (PAS) staining, and immunostaining. Inflammasome activation and interstitial immune cell infiltration was evaluated in the 4-μm kidney sections by using Abs against IL-1β (12242, Cell Signaling Technology), p20 (AG-20B-0042, AdipoGen), a macrophage marker (F4/80, sc-101447, Santa Cruz Biotechnology), or a neutrophil marker (NIMP-14, sc-59338, Santa Cruz Biotechnology). In at least five fields per kidney, the amount of secreted IL-1β and active caspase-1 (p20) was calculated as the stained area per field using the freeware ImageJ. The number of interstitial cells in the corticomedullary junction was counted in a randomly selected slide at 40x magnification, and at least five fields were counted per kidney section.

### MSU crystal-induced arthritis

Briefly, MSU crystals were dissolved in PBS and then sonicated at 100% Amp three times for 10 min each. Sonicated MSU crystals (2.5 mg 100 μl^−1^ mice^−1^) were subcutaneously injected under the plantar surface of the paws of *Ncoa6*^+/−^ mice and WT mice. Paw and ankle swelling were measured with an electronic caliper by a researcher blinded to genotype at the indicated time points. Six or twenty-four hours after the injection of MSU crystals, tissues were collected from the affected joints and fixed with 4% paraformaldehyde for 24 h. The fixed tissue was decalcified with decalcifying solution (Sigma‒Aldrich) for 36 h, paraffin-embedded, and sectioned at 4 μm. For pathohistological analysis, the sectioned slides were processed for H&E staining and immunohistochemical staining using Abs against IL-1β, p20, a macrophage marker or a neutrophil marker. The same experiment was repeated with myelomonocytic cell-specific *Ncoa6*-deficient mice and *Ncoa6* ^*fl/fl*^ mice. The histological severity of gouty arthritis was assessed as previously described [60].

### Isolation of synovial fluid mononuclear cells and synovial tissues

Synovial fluids were freshly isolated from patients with gouty arthritis (n = 12) who fulfilled the 2015 ACR/EULAR gout classification criteria [72] during arthrocentesis. The mean age of the gout patients was 49.5 ± 22.3 years. All of the gout patients were male, and written informed consent was obtained from all of the patients included in this study. Mononuclear cells were isolated from the synovial fluids. In some experiments, CD14^+^ macrophages were purified from mononuclear cells by magnetic separation using anti-CD14 beads (Miltenyi Biotech, Surrey, UK) according to the manufacturer’s instructions. Synovial tissues were also obtained from patients with gout (n = 3), RA (n = 3, who fulfilled the ACR/EULAR 2010 classification criteria) [73], and osteoarthritis (n = 3) during synovectomy and/or total joint replacement surgery.

This study was performed with the approval of the institutional review board (KC19SESI0115).

### Statistical analysis

Statistical analysis was conducted using GraphPad Prism software. Data are shown as the mean ± standard error of the mean (SEM). A paired or unpaired *t* test was used for statistical evaluations as indicated in each group. Comparisons of two or more groups were performed using one- or two-way analysis of variance (ANOVA). *P* < 0.05 was considered to indicate statistical significance. Fisher’s exact test was used to compute the significant associations between the *Ncoa6*-KO DEGs and the DEGs related to immune disorders.

### Study approval

The study protocol was approved by the Institutional Review Board of Seoul St. Mary’s Hospital, The Catholic University of Korea (Approval number: KC19SESI0115). All study participants provided written informed consent. All animal research procedures were performed in accordance with the Laboratory Animals Welfare Act, the Guide for the Care and Use of Laboratory Animals, and the Guidelines and Policies for Rodent Experiments provided by the Institutional Animal Care and Use Committee (IACUC) in the School of Medicine, The Catholic University of Korea (Approval number: CUMC-2020-0332-01).

### Supplementary information

supplementary Fig 1-16 (non-highligted)

Unprocessed images of gesl and western blots (revised)

Supplementary video-1

Supplementary video-2

supplementary table 1-5

## Data Availability

The RNA-sequencing data are available in the GEO database under accession number GSE207875. All other data needed to evaluate the conclusions in the paper are present in the paper or the Supplementary Materials.

## References

[1] Naar AM, Lemon BD, Tjian R (2001). Transcriptional coactivator complexes. Annu Rev Biochem.

[2] Rosenfeld MG, Lunyak VV, Glass CK (2006). Sensors and signals: a coactivator/corepressor/epigenetic code for integrating signal-dependent programs of transcriptional response. Genes Dev.

[3] Lonard DM, O’Malley BW (2012). Nuclear receptor coregulators: modulators of pathology and therapeutic targets. Nat Rev Endocrinol.

[4] Lonard DM, O’Malley BW (2007). Nuclear receptor coregulators: judges, juries, and executioners of cellular regulation. Mol Cell.

[5] Szklarczyk D, Gable AL, Nastou KC, Lyon D, Kirsch R, Pyysalo S (2021). The STRING database in 2021: customizable protein-protein networks, and functional characterization of user-uploaded gene/measurement sets. Nucleic Acids Res.

[6] Lee SK, Anzick SL, Choi JE, Bubendorf L, Guan XY, Jung YK (1999). A nuclear factor, ASC-2, as a cancer-amplified transcriptional coactivator essential for ligand-dependent transactivation by nuclear receptors in vivo. J Biol Chem.

[7] Mahajan MA, Das S, Zhu H, Tomic-Canic M, Samuels HH (2004). The nuclear hormone receptor coactivator NRC is a pleiotropic modulator affecting growth, development, apoptosis, reproduction, and wound repair. Mol Cell Biol.

[8] Caira F, Antonson P, Pelto-Huikko M, Treuter E, Gustafsson JA (2000). Cloning and characterization of RAP250, a novel nuclear receptor coactivator. J Biol Chem.

[9] Kim SW, Park K, Kwak E, Choi E, Lee S, Ham J (2003). Activating signal cointegrator 2 required for liver lipid metabolism mediated by liver X receptors in mice. Mol Cell Biol.

[10] Mahajan MA, Samuels HH (2008). Nuclear receptor coactivator/coregulator NCoA6(NRC) is a pleiotropic coregulator involved in transcription, cell survival, growth and development. Nucl Recept Signal.

[11] Oh GS, Kim SR, Lee ES, Yoon J, Shin MK, Ryu HK (2022). Regulation of Hepatic Gluconeogenesis by Nuclear Receptor Coactivator 6. Mol Cells.

[12] Kim GH, Park K, Yeom SY, Lee KJ, Kim G, Ko J (2009). Characterization of ASC-2 as an antiatherogenic transcriptional coactivator of liver X receptors in macrophages. Mol Endocrinol.

[13] Hong S, Choi HM, Park MJ, Kim YH, Choi YH, Kim HH (2004). Activation and interaction of ATF2 with the coactivator ASC-2 are responsive for granulocytic differentiation by retinoic acid. J Biol Chem.

[14] Valtierra-Alvarado MA, Castaneda Delgado JE, Ramirez-Talavera SI, Lugo-Villarino G, Duenas-Arteaga F, Lugo-Sanchez A (2020). Type 2 diabetes mellitus metabolic control correlates with the phenotype of human monocytes and monocyte-derived macrophages. J Diabetes Complications.

[15] Shalhoub J, Falck-Hansen MA, Davies AH, Monaco C (2011). Innate immunity and monocyte-macrophage activation in atherosclerosis. J Inflamm.

[16] Conte E (2022). Targeting monocytes/macrophages in fibrosis and cancer diseases: therapeutic approaches. Pharm Ther.

[17] Yoneda Y (2000). Nucleocytoplasmic protein traffic and its significance to cell function. Genes Cells.

[18] Li Q, Xu J (2011). Identification and characterization of the alternatively spliced nuclear receptor coactivator-6 isoforms. Int J Biol Sci.

[19] Brand DD, Latham KA, Rosloniec EF (2007). Collagen-induced arthritis. Nat Protoc.

[20] You S, Yoo SA, Choi S, Kim JY, Park SJ, Ji JD (2014). Identification of key regulators for the migration and invasion of rheumatoid synoviocytes through a systems approach. Proc Natl Acad Sci USA.

[21] Mattaj IW, Englmeier L (1998). Nucleocytoplasmic transport: the soluble phase. Annu Rev Biochem.

[22] Bryan NB, Dorfleutner A, Rojanasakul Y, Stehlik C (2009). Activation of inflammasomes requires intracellular redistribution of the apoptotic speck-like protein containing a caspase recruitment domain. J Immunol.

[23] Burnett BG, Pittman RN (2005). The polyglutamine neurodegenerative protein ataxin 3 regulates aggresome formation. Proc Natl Acad Sci USA.

[24] Xiong R, Siegel D, Ross D (2013). The activation sequence of cellular protein handling systems after proteasomal inhibition in dopaminergic cells. Chem Biol Interact.

[25] Ghonime MG, Shamaa OR, Das S, Eldomany RA, Fernandes-Alnemri T, Alnemri ES (2014). Inflammasome priming by lipopolysaccharide is dependent upon ERK signaling and proteasome function. J Immunol.

[26] Broz P, Dixit VM (2016). Inflammasomes: mechanism of assembly, regulation and signalling. Nat Rev Immunol.

[27] Bauernfeind FG, Horvath G, Stutz A, Alnemri ES, MacDonald K, Speert D (2009). Cutting edge: NF-kappaB activating pattern recognition and cytokine receptors license NLRP3 inflammasome activation by regulating NLRP3 expression. J Immunol.

[28] Martinon F, Burns K, Tschopp J (2002). The inflammasome: a molecular platform triggering activation of inflammatory caspases and processing of proIL-beta. Mol Cell.

[29] Mariathasan S, Weiss DS, Newton K, McBride J, O’Rourke K, Roose-Girma M (2006). Cryopyrin activates the inflammasome in response to toxins and ATP. Nature..

[30] Martinon F, Petrilli V, Mayor A, Tardivel A, Tschopp J (2006). Gout-associated uric acid crystals activate the NALP3 inflammasome. Nature.

[31] Fernandes-Alnemri T, Wu J, Yu JW, Datta P, Miller B, Jankowski W (2007). The pyroptosome: a supramolecular assembly of ASC dimers mediating inflammatory cell death via caspase-1 activation. Cell Death Differ.

[32] Bagchi S, Fredriksson R, Wallen-Mackenzie A (2015). In situ proximity ligation assay (PLA). Methods Mol Biol.

[33] Piccini A, Carta S, Tassi S, Lasiglie D, Fossati G, Rubartelli A (2008). ATP is released by monocytes stimulated with pathogen-sensing receptor ligands and induces IL-1beta and IL-18 secretion in an autocrine way. Proc Natl Acad Sci USA.

[34] He Y, Zeng MY, Yang D, Motro B, Nunez G (2016). NEK7 is an essential mediator of NLRP3 activation downstream of potassium efflux. Nature..

[35] Sutterwala FS, Ogura Y, Flavell RA (2007). The inflammasome in pathogen recognition and inflammation. J Leukoc Biol.

[36] Brinkschulte R, Fussholler DM, Hoss F, Rodriguez-Alcazar JF, Lauterbach MA, Kolbe CC (2022). ATP-binding and hydrolysis of human NLRP3. Commun Biol..

[37] Neven B, Callebaut I, Prieur AM, Feldmann J, Bodemer C, Lepore L (2004). Molecular basis of the spectral expression of CIAS1 mutations associated with phagocytic cell-mediated autoinflammatory disorders CINCA/NOMID, MWS, and FCU. Blood..

[38] Subramanian N, Natarajan K, Clatworthy MR, Wang Z, Germain RN (2013). The adaptor MAVS promotes NLRP3 mitochondrial localization and inflammasome activation. Cell..

[39] Lee HE, Yang G, Kim ND, Jeong S, Jung Y, Choi JY (2016). Targeting ASC in NLRP3 inflammasome by caffeic acid phenethyl ester: a novel strategy to treat acute gout. Sci Rep.

[40] Eisenbarth SC, Colegio OR, O’Connor W, Sutterwala FS, Flavell RA (2008). Crucial role for the Nalp3 inflammasome in the immunostimulatory properties of aluminium adjuvants. Nature..

[41] Kingsbury SR, Conaghan PG, McDermott MF (2011). The role of the NLRP3 inflammasome in gout. J Inflamm Res.

[42] Jeong JH, Jung JH, Lee JS, Oh JS, Kim YG, Lee CK (2019). Prominent inflammatory features of monocytes/macrophages in acute calcium pyrophosphate crystal arthritis: a comparison with acute gouty arthritis. Immune Netw.

[43] Martinon F, Glimcher LH (2006). Gout: new insights into an old disease. J Clin Investig.

[44] Schauer C, Janko C, Munoz LE, Zhao Y, Kienhofer D, Frey B (2014). Aggregated neutrophil extracellular traps limit inflammation by degrading cytokines and chemokines. Nat Med.

[45] Palmer DG, Hogg N, Denholm I, Allen CA, Highton J, Hessian PA (1987). Comparison of phenotype expression by mononuclear phagocytes within subcutaneous gouty tophi and rheumatoid nodules. Rheumatol Int.

[46] Dalbeth N, Pool B, Gamble GD, Smith T, Callon KE, McQueen FM (2010). Cellular characterization of the gouty tophus: a quantitative analysis. Arthritis Rheum.

[47] Dalbeth N, Choi HK, Joosten LAB, Khanna PP, Matsuo H, Perez-Ruiz F (2019). Gout. Nat Rev Dis Prim.

[48] Burns CM, Wortmann RL (2011). Gout therapeutics: new drugs for an old disease. Lancet..

[49] Shaffer KL, Sharma A, Snapp EL, Hegde RS (2005). Regulation of protein compartmentalization expands the diversity of protein function. Dev Cell.

[50] Caputto BL, Cardozo Gizzi AM, Gil GA (2014). c-Fos: an AP-1 transcription factor with an additional cytoplasmic, non-genomic lipid synthesis activation capacity. Biochim Biophys Acta.

[51] Pawlak A, Strzadala L, Kalas W (2015). Non-genomic effects of the NR4A1/Nur77/TR3/NGFIB orphan nuclear receptor. Steroids..

[52] Alatshan A, Benko S (2021). Nuclear receptors as multiple regulators of NLRP3 inflammasome function. Front Immunol.

[53] Duncan JA, Bergstralh DT, Wang Y, Willingham SB, Ye Z, Zimmermann AG (2007). Cryopyrin/NALP3 binds ATP/dATP, is an ATPase, and requires ATP binding to mediate inflammatory signaling. Proc Natl Acad Sci USA.

[54] Shaw OM, Steiger S, Liu X, Hamilton JA, Harper JL (2014). Brief report: Granulocyte-macrophage colony-stimulating factor drives monosodium urate monohydrate crystal-induced inflammatory macrophage differentiation and NLRP3 inflammasome up-regulation in an in vivo mouse model. Arthritis Rheumatol.

[55] Said-Sadier N, Ojcius DM (2012). Alarmins, inflammasomes and immunity. Biomed J..

[56] So AK, Martinon F (2017). Inflammation in gout: mechanisms and therapeutic targets. Nat Rev Rheumatol.

[57] Rao P, Falk LA, Dougherty SF, Sawada T, Pluznik DH (1997). Colchicine down-regulates lipopolysaccharide-induced granulocyte-macrophage colony-stimulating factor production in murine macrophages. J Immunol.

[58] Misawa T, Takahama M, Kozaki T, Lee H, Zou J, Saitoh T (2013). Microtubule-driven spatial arrangement of mitochondria promotes activation of the NLRP3 inflammasome. Nat Immunol.

[59] Kuang SQ, Liao L, Zhang H, Pereira FA, Yuan Y, DeMayo FJ (2002). Deletion of the cancer-amplified coactivator AIB3 results in defective placentation and embryonic lethality. J Biol Chem.

[60] Choi S, You S, Kim D, Choi SY, Kwon HM, Kim HS (2017). Transcription factor NFAT5 promotes macrophage survival in rheumatoid arthritis. J Clin Investig.

[61] Ewels P, Magnusson M, Lundin S, Kaller M (2016). MultiQC: summarize analysis results for multiple tools and samples in a single report. Bioinformatics..

[62] Dobin A, Davis CA, Schlesinger F, Drenkow J, Zaleski C, Jha S (2013). STAR: ultrafast universal RNA-seq aligner. Bioinformatics..

[63] Li B, Dewey CN (2011). RSEM: accurate transcript quantification from RNA-Seq data with or without a reference genome. BMC Bioinform.

[64] Thorvaldsdottir H, Robinson JT, Mesirov JP (2013). Integrative Genomics Viewer (IGV): high-performance genomics data visualization and exploration. Brief Bioinform.

[65] Wang L, Wang S, Li W (2012). RSeQC: quality control of RNA-seq experiments. Bioinformatics..

[66] Soneson C, Love MI, Robinson MD (2015). Differential analyses for RNA-seq: transcript-level estimates improve gene-level inferences. F1000Res.

[67] Love MI, Huber W, Anders S (2014). Moderated estimation of fold change and dispersion for RNA-seq data with DESeq2. Genome Biol.

[68] Robinson MD, McCarthy DJ, Smyth GK (2010). edgeR: a Bioconductor package for differential expression analysis of digital gene expression data. Bioinformatics.

[69] Yu G, Wang LG, Han Y, He QY (2012). clusterProfiler: an R package for comparing biological themes among gene clusters. OMICS.

[70] Korotkevich G, Sukhov V, Budin N, Shpak B, Artyomov MN, Sergushichev A. Fast gene set enrichment analysis. BioRxiv. 2021; 10.1101/060012.

[71] Yang CS, Kim JJ, Kim TS, Lee PY, Kim SY, Lee HM (2015). Small heterodimer partner interacts with NLRP3 and negatively regulates activation of the NLRP3 inflammasome. Nat Commun.

[72] Neogi T, Jansen TL, Dalbeth N, Fransen J, Schumacher HR, Berendsen D (2015). 2015 Gout classification criteria: an American College of Rheumatology/European League Against Rheumatism collaborative initiative. Ann Rheum Dis.

[73] Kay J, Upchurch KS (2012). ACR/EULAR 2010 rheumatoid arthritis classification criteria. Rheumatology.

