# Supplementary material for: Nuclear receptor coactivator 6 is a critical regulator of NLRP3 inflammasome activation and gouty arthritis
Source: Cell Mol Immunol. 2024 Jan 10;21(3):227–44. doi: 10.1038/s41423-023-01121-x (PMC10902316; doi:10.1038/s41423-023-01121-x)
Supplement: Supplementary file 1 — supplementary Fig 1-16 (non-highligted) [file 41423_2023_1121_MOESM1_ESM.pdf]

Nuclear Receptor Coactivator 6 is a Critical Regulator of NLRP3 Inflammasome Activation and Gouty Arthritis

Kang-Gu Lee[1,2], Bong-Ki Hong[1], Saseong Lee[1], Naeun Lee[1], Seung-Whan Kim[5,6], Donghyun Kim[3,4], and *Wan-Uk Kim[1,2,7]

[1]Center for Integrative Rheumatoid Transcriptomics and Dynamics, The Catholic University of Korea, Seoul 06591, Republic of Korea.
[2]Department of Biomedicine & Health Sciences, The Catholic University of Korea, Seoul 06591, Republic of Korea.
[3]Department of Microbiology and Immunology, Seoul National University College of Medicine, Seoul 03080, Republic of Korea
[4]Institute of Infectious Diseases, Seoul National University College of Medicine, Seoul 03080, Republic of Korea.
[5]Department of Pharmacology, Asan Medical Center, University of Ulsan College of Medicine, Seoul 05505, Republic of Korea.
[6]Bio-Medical Institute of Technology, University of Ulsan, Seoul 05505, Korea.
[7]Division of Rheumatology, Department of Internal Medicine, The Catholic University of Korea, Seoul 06591, Republic of Korea.

*Correspondence and reprint requests:

Dr. Wan-Uk Kim, Division of Rheumatology, Department of Internal Medicine, Catholic University of Korea, School of Medicine, Seoul, Korea (E-mail: wan725@catholic.ac.kr).

This PDF file includes:

Supplementary Figure 1 to 16

Legends for Supplementary Table 1 to 5

Legends for Supplementary Video 1 and 2

Other Supplementary Material for this manuscript includes the following:

Supplementary Table 1 to 5

Supplementary Video 1 and 2

33 # Supplementary Figures

A

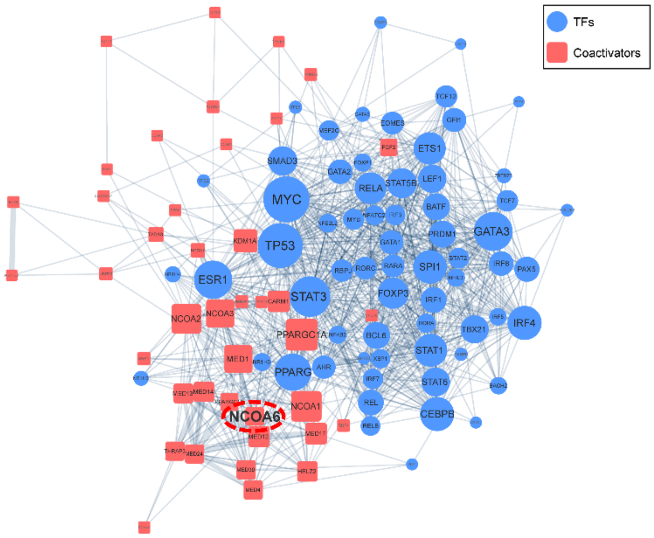

B

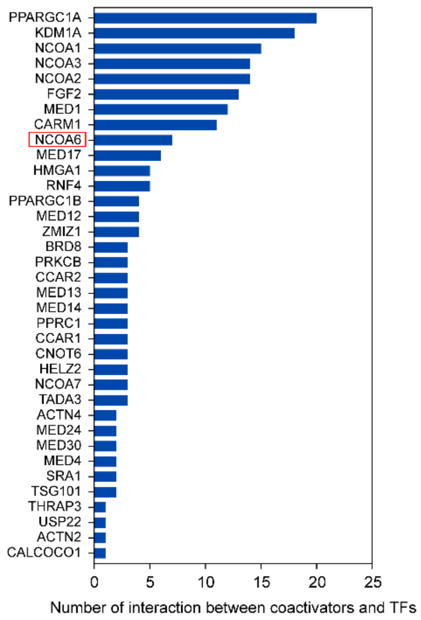

C

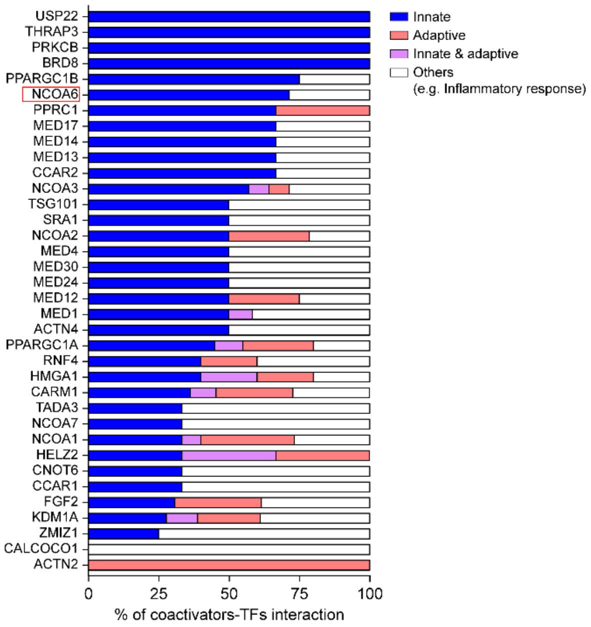

D

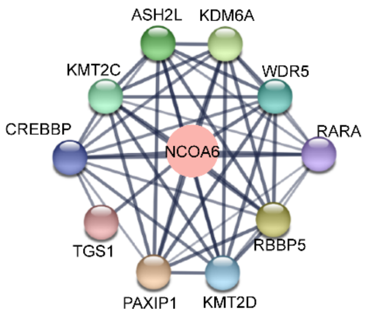

E

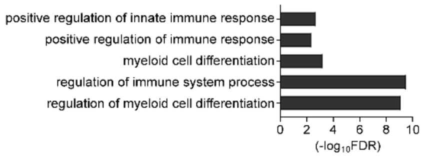

Biological process (GO)	Count in gene set	FDR
positive regulation of innate immune response	2	0.0453
positive regulation of immune response	3	0.0214
myeloid cell differentiation	3	0.0015
regulation of immune system process	8	3.13e-06
regulation of myeloid cell differentiation	7	1.21e-09

Supplementary Figure 1. Reconstruction of molecular network of transcription factors (TFs) related to "immune response" and nuclear coactivators. (A) The protein-protein interaction network of 65 immune response-related TFs and 41 coactivators as defined by the Gene Ontology database. The size of each node and label are determined by the degree of interactions. The blue circle and red rounded rectangle denote TF and coactivator, respectively. **(B and C)** Bar graph showing the number of interactions of each nuclear receptor coactivator with immune response-related TFs (b). Stacked bar graph showing the proportion of TFs involved in "innate response" versus "adaptive response" over "immune response"-related TFs for each coactivator interacting with the TFs (c). **(D and E)** NCOA6-bound proteins analyzed using the STRING database. Predicted functional proteins that interact with NCOA6 (D). Immune-related biological processes enriched by Gene Ontology (GO) analysis of NCOA6-associated proteins (E). The bar graph shows the enrichment score of each GO term, ranked by the false discovery rate (FDR).

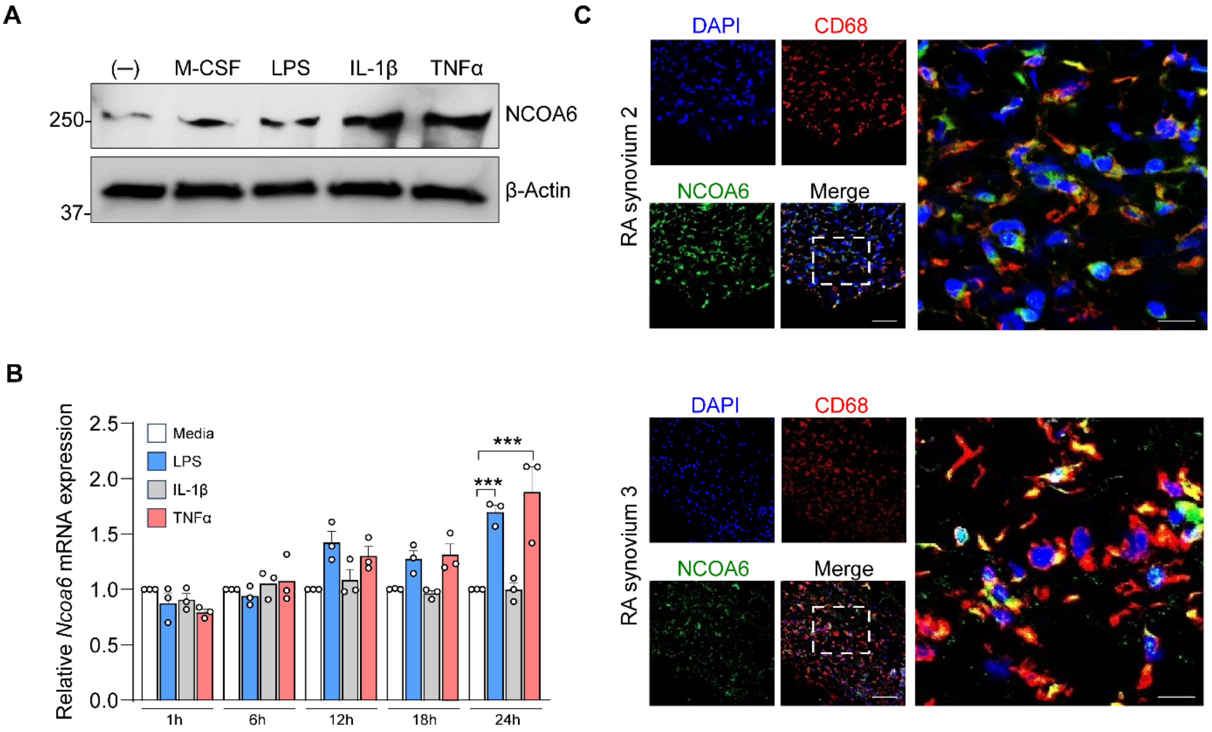

Supplementary Figure 2. NCOA6 expression in primary human monocytes and the rheumatoid synovium. (A) NCOA6 protein expression in human monocytes in response to proinflammatory stimuli. Human CD14^{+} cells isolated from the peripheral blood of healthy subjects were stimulated with M-CSF (20 ng/mL), LPS (10 ng/mL), IL-1β (10 ng/mL), and TNFα (10 ng/mL). NCOA6 expression levels were determined by Western blot analysis after 2 days. **(B)** Time-dependent expression of *Ncoa6* mRNA in murine macrophages stimulated with LPS, IL-1β, and TNFα. **(C)** Immunofluorescence images of CD68 and NCOA6 in the synovium of another two patients with rheumatoid arthritis (RA) (*See* **Figure 1G**). Nuclei were stained with DAPI. The rectangular areas in the middle panel are magnified in the right panel. Scale bars=40 μm for the middle and 20 μm for the right.

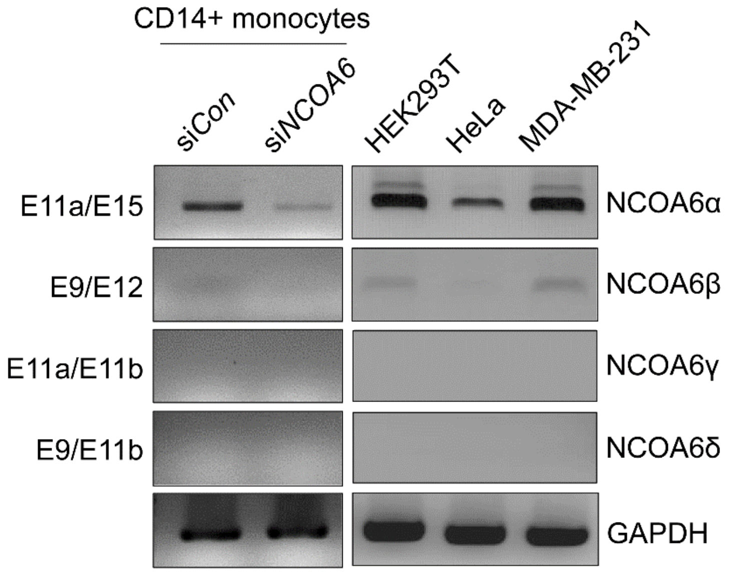

Supplementary Figure 3. Conventional RT–PCR products amplified from *NCOA6* mRNA using the indicated PCR primer pair in human CD14[+] cells transfected with *NCOA6* siRNA or control siRNA, HEK293T cells, HeLa cells and human breast cancer cells (MDA-MB-231).

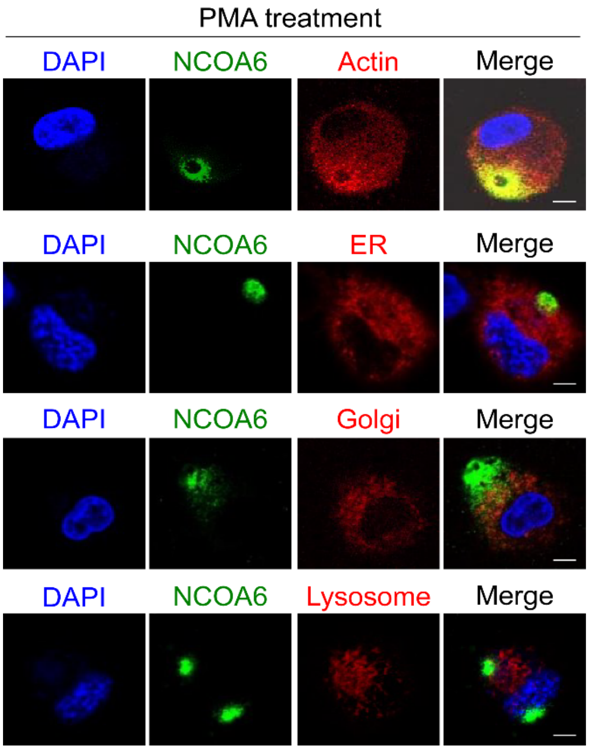

Supplementary Figure 4. Double immunofluorescence staining of differentiated THP-1 cells. The cells were stained with Abs for NCOA6 and markers of the ER, Golgi, and lysosome. Nuclei were stained with DAPI. Scale bars, 20 μm. The images are representative of at least three independent experiments.

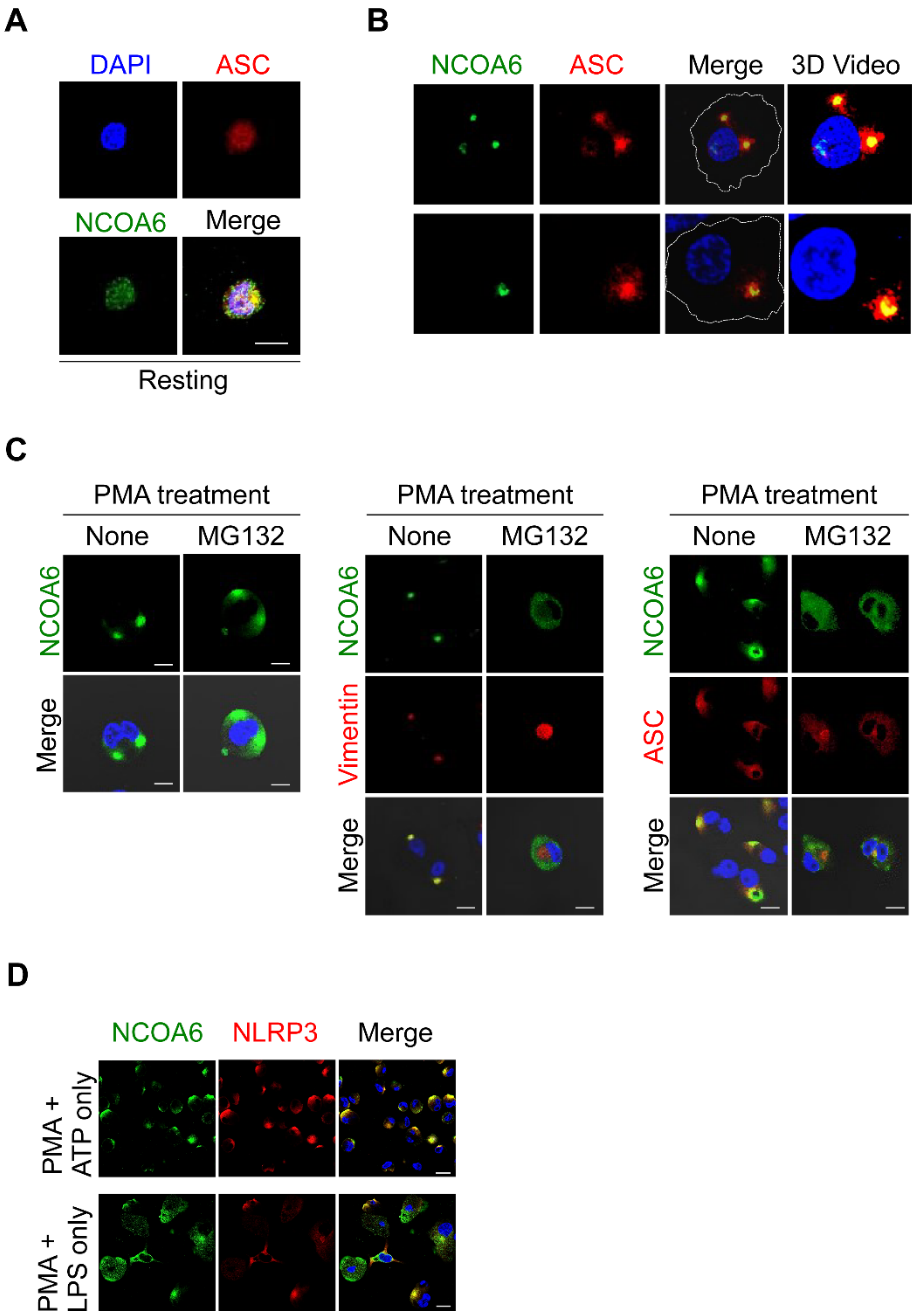

Supplementary Figure 5. Colocalization of NCOA6 with NLRP3 inflammasome components in the cytoplasm of THP-1 cells. (A and B) Immunocytochemistry images of resting (**A**) and PMA-stimulated THP-1 cells primed with LPS, followed by ATP treatment (**B**). **(C)** Immunofluorescence staining for NCOA6, ASC, and vimentin in THP-1 cells treated with MG132. The cells were treated with MG132 for 1 hour, followed by PMA for 72 hours. **(D)** Immunocytochemistry images of differentiated THP-1 cells stimulated with LPS only or ATP only. Scale bars, 10 μm.

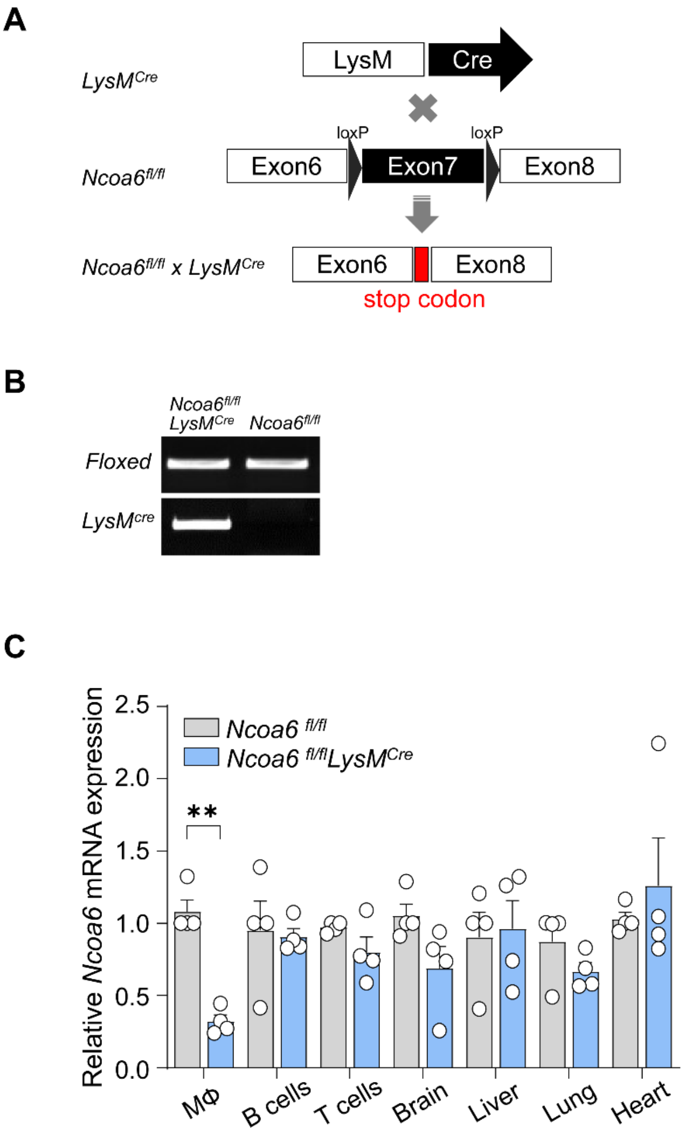

Supplementary Figure 6. Generation of myeloid-specific *Ncoa6*-KO mice. (A) Schematic drawing of the generation of *Ncoa6*$^{fl/fl}$*LysM*^{CRE} mice. *LysM*^{CRE} mice were mated with *Ncoa6*$^{fl/fl}$ mice, which have a loxP site flanking exon 7 of the *Ncoa6* gene. **(B)** Genotyping of genomic DNA extracted from tail tissues of *Ncoa6*$^{fl/fl}$ mice and *Ncoa6*$^{fl/fl}$*LysM*^{CRE} mice. **(C)** qRT–PCR analysis of *Ncoa6* mRNA expression in the myeloid cells, lymphoid cells, brain, liver, lung, and heart of mice.

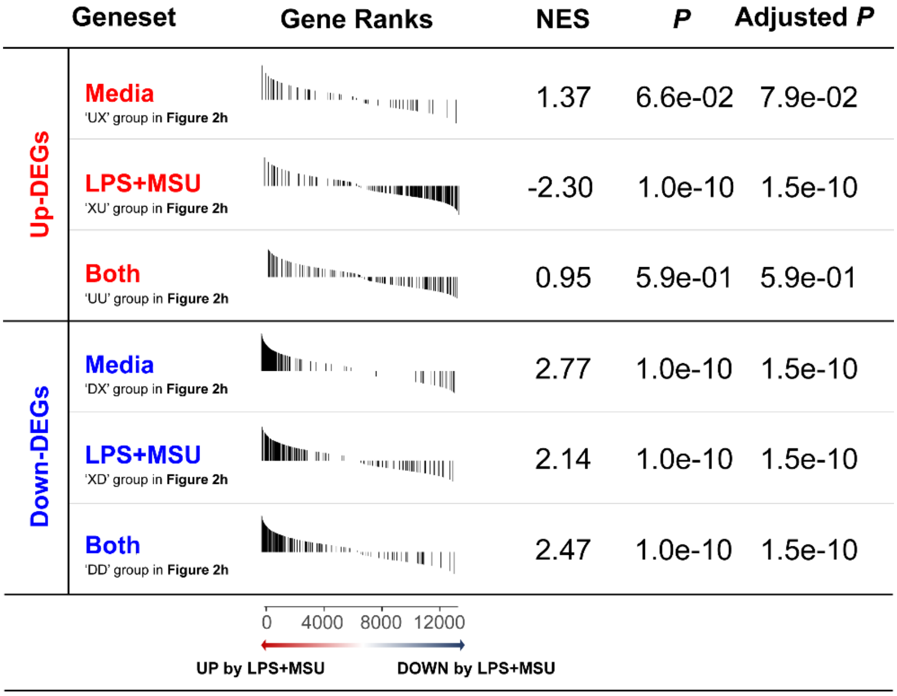

Supplementary Figure 7. Plot GSEA table showing the GSEA analysis results of DEGs in *Ncoa6*-KO macrophages. GSEA results of six groups of DEGs (*See* heatmap groups in Figure 2h) show that the three gene sets of downregulated DEGs (Down-DEGs) in *Ncoa6*-KO BMDMs are significantly upregulated in *Ncoa6*-sufficient BMDMs with LPS+MSU as compared to media alone. On the contrary, a gene set of upregulated DEGs (Up-DEGs) with LPS+MSU is significantly downregulated in *Ncoa6*-sufficient BMDMs with LPS+MSU; the other two sets of upregulated DEGs have no correlation. Black vertical tick marks in the "Gene Ranks" column indicate the location of individual DEGs in *Ncoa6*-KO BMDMs within the fold change-ranked gene list. The height of each black vertical tick marks indicates the fold change values of individual DEGs. NES denotes the normalized enrichment score.

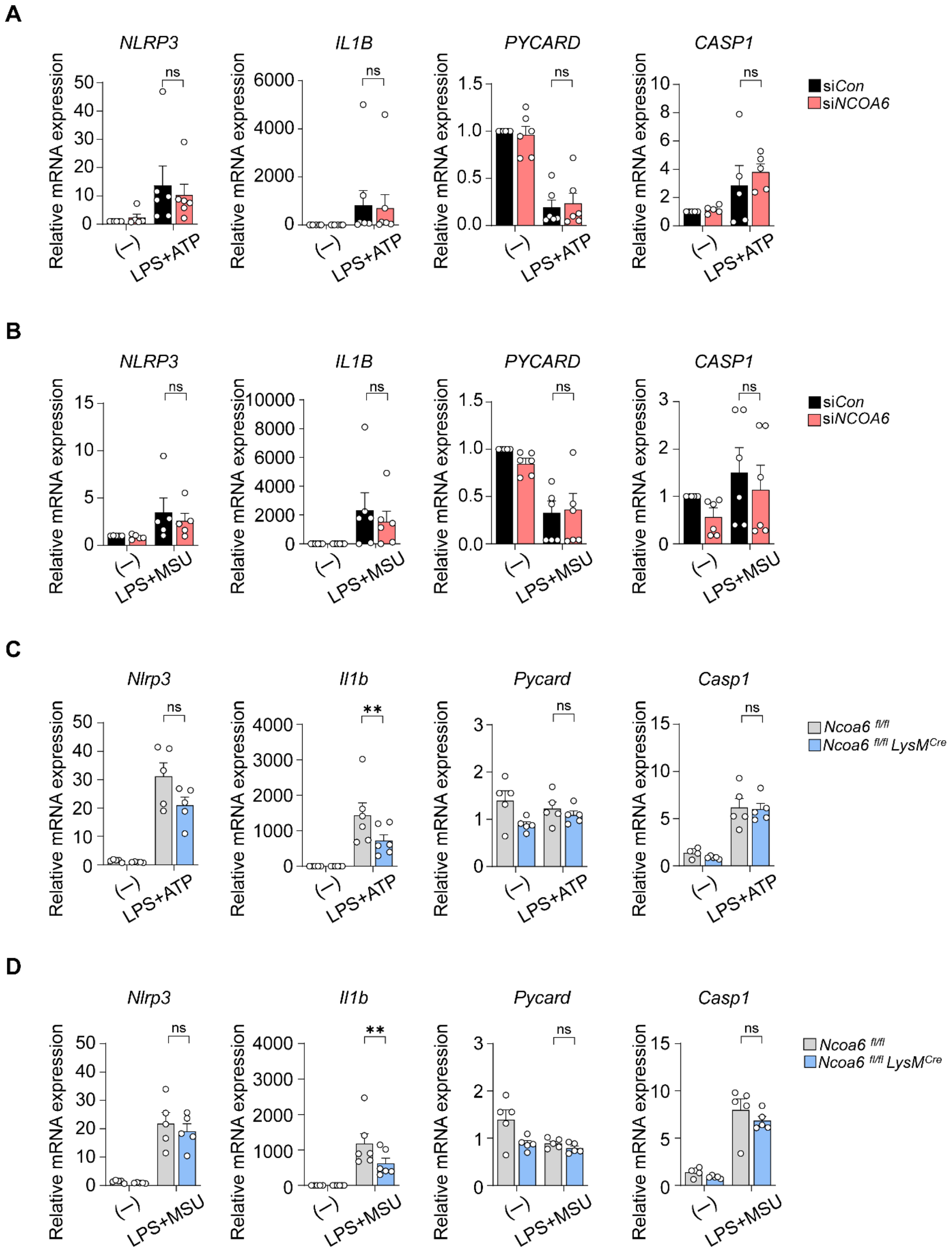

Supplementary Figure 8. Expression levels of inflammasome-related genes in *NCOA6*-deficient monocytes and macrophages. *NCOA6* siRNA-transfected human monocytes (**A and B**) and BMDMs from *Ncoa6*$^{fl/fl}$ and *Ncoa6*$^{fl/fl}$*LysM*^{CRE} mice (**C and D**) were primed with LPS, followed by ATP or MSU crystal treatment. The mRNA expression levels of inflammasome-related genes, including *NLRP3, IL1B, PYCARD*, and *CASP1,* were determined by qRT–PCR. Each dot represents an individual donor (A and B) or mouse (C and D). ***P* < 0.01 by paired two-tailed t test (A and B) or unpaired two-tailed t test (C and D).

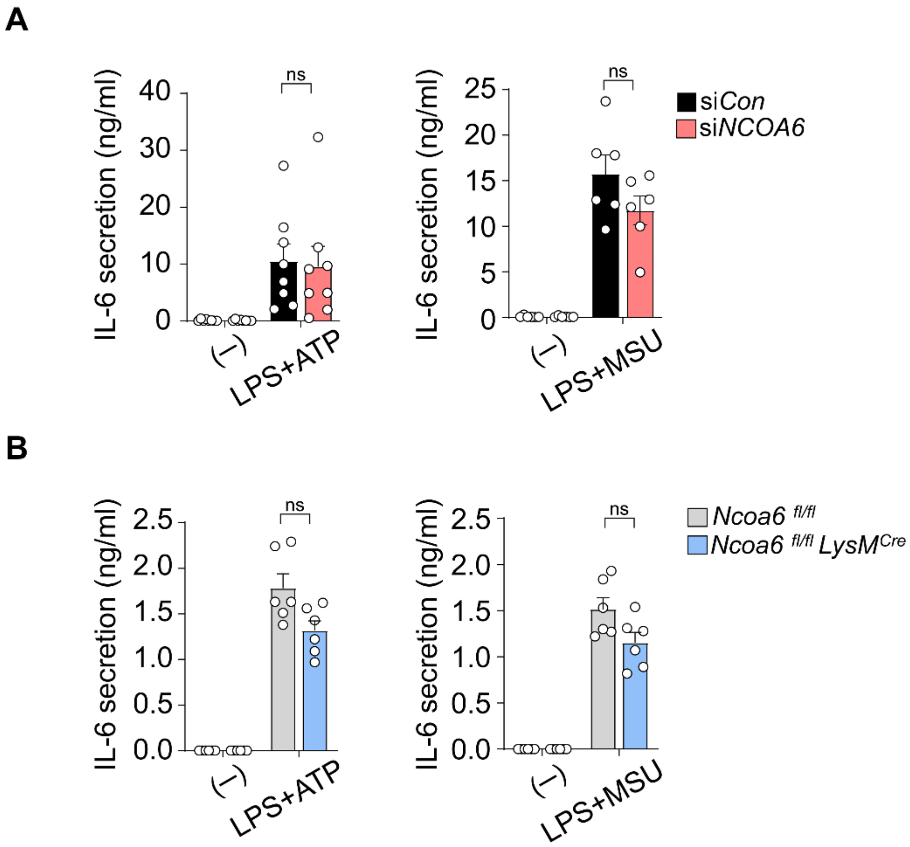

Supplementary Figure 9. No change in IL-6 secretion by *NCOA6* deficiency. Human CD14$^+$ cells transfected with siRNA (n=6, each group) (**A**) and BMDMs from *Ncoa6*$^{fl/fl}$ and *Ncoa6*$^{fl/fl}$*LysM*^{CRE} mice (n=6, each group) (**B**) were primed with LPS, followed by ATP or MSU crystal treatment. IL-6 secretion in the culture supernatant was assessed by ELISA. Each dot represents an individual donor or mouse. *$P < 0.05$ and **$P < 0.01$ by paired two-tailed t test (A) or unpaired two-tailed t test (B).

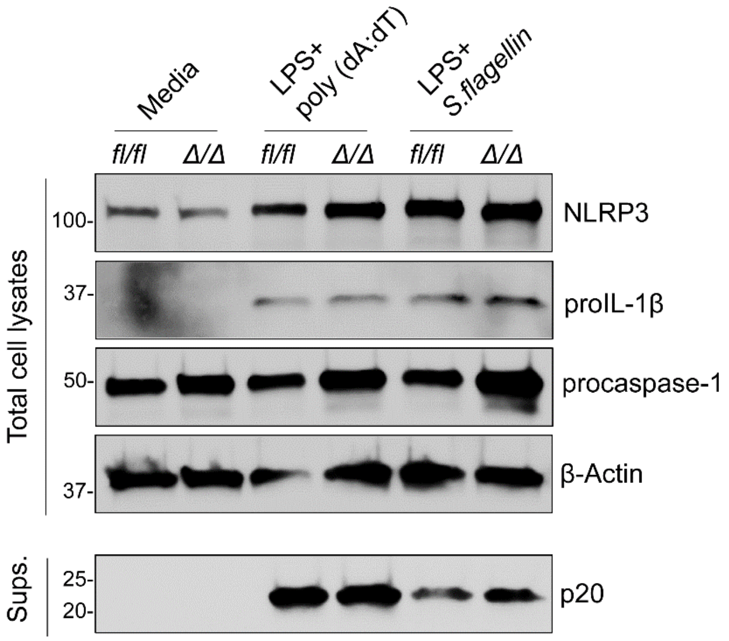

Supplementary Figure 10. **Expression of Active caspase-1 in *Ncoa6*-KO BMDMs activated by NLRC4 or AIM2 inflammasome.** BMDMs, isolated from *Ncoa6*$^{fl/fl}$ and *Ncoa6*$^{fl/fl}$*LysM*^{CRE} mice, were primed with LPS (100 ng/mL) for 4 hours and then transfected with S. Typhimurium flagellin or poly (dA:dT) using Lipofectamine 3000. After 4 hours, cell lysates and culture supernatants were harvested and immunoblotted with the indicated antibodies. Sups. denotes supernatants.

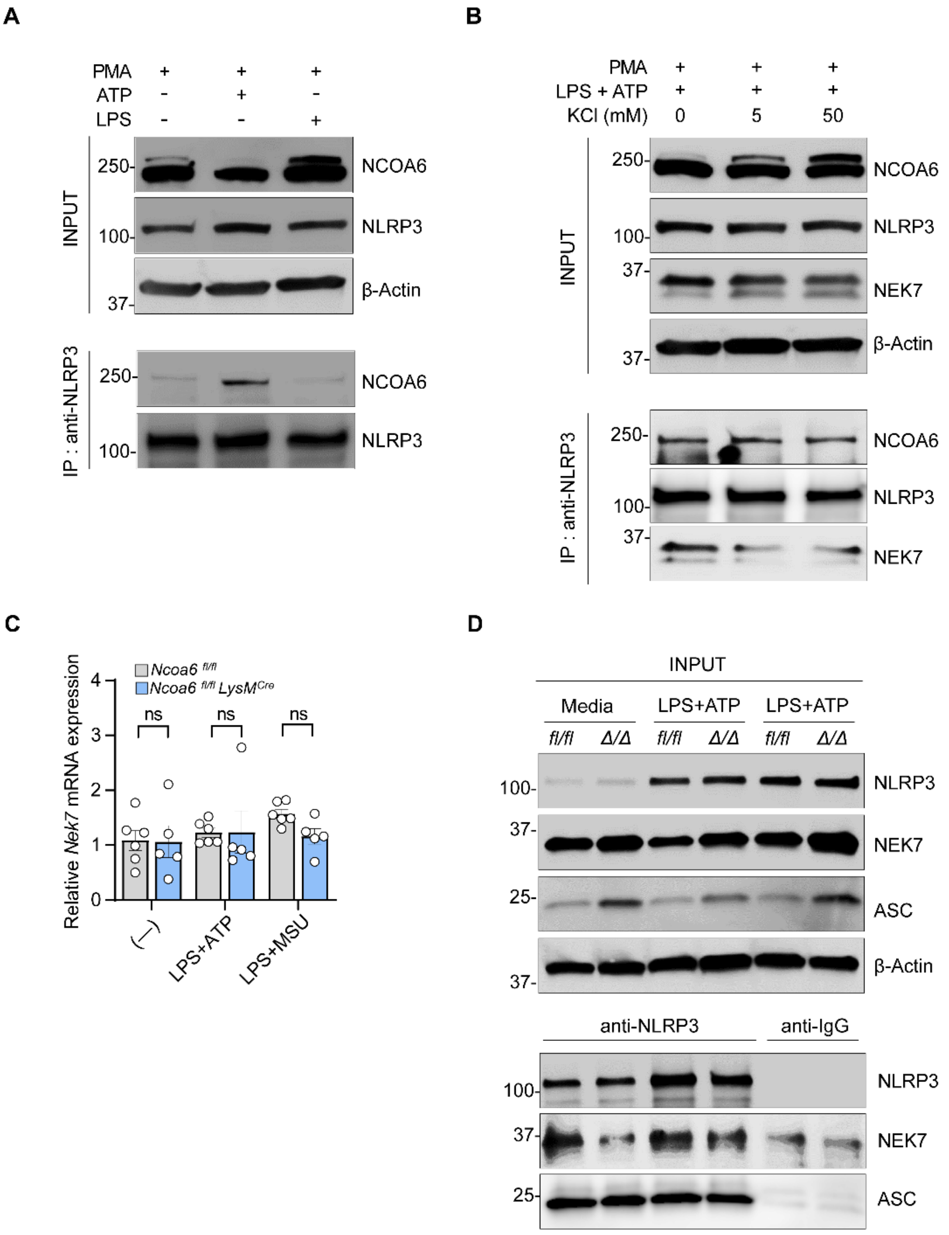

Supplementary Figure 11. No association of potassium efflux and NEK7 with NCOA6-mediated NLRP3 activation. A, B Cells were treated with LPS (100 ng/mL) for 4 hours or ATP (5 mM) for 30 minutes. Immunoprecipitation assay of NCOA6 and NEK7 using anti-NLRP3 Abs in the absence (A) or presence of extracellular KCl (B). **(C)** *Nek7* mRNA expression in *Ncoa6*$^{fl/fl}$ and *Ncoa6*$^{fl/fl}$*LysM*^{CRE} BMDMs activated by the indicated stimuli. Each dot represents an individual mouse. **(D)** BMDMs of *Ncoa6*$^{fl/fl}$ and *Ncoa6*$^{fl/fl}$*LysM*^{CRE} mice were primed with LPS (100 ng/mL) and then stimulated with ATP for 30 minutes. Cell lysates were immune-precipitated and immunoblotted with the indicated antibodies. Immunoblotting images are representative of at least three independent experiments.

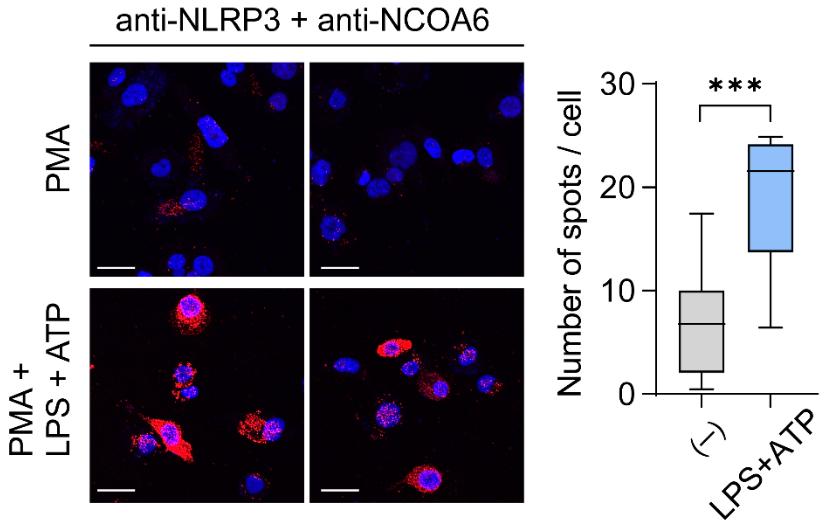

Supplementary Figure 12. **Proximity ligation assay (PLA) in PMA-stimulated THP-1 cells.** The cells were treated with LPS (100 ng ml^{-1}) for 4 hours and then were stimulated by ATP (5 mM) for 30 minutes. Red fluorescent dots indicate sites of interaction between NCOA6 and NLRP3 proteins. The images in the left panel are representative of at least three independent experiments. The number of red fluorescent dots for each Z-stack was divided by the total number of cells labeled with DAPI per image and is presented as a bar graph (right panel). At least 50 cells per field were randomly counted. ***P < 0.001 by unpaired two-tailed t test. Scale bars, 20 μm.

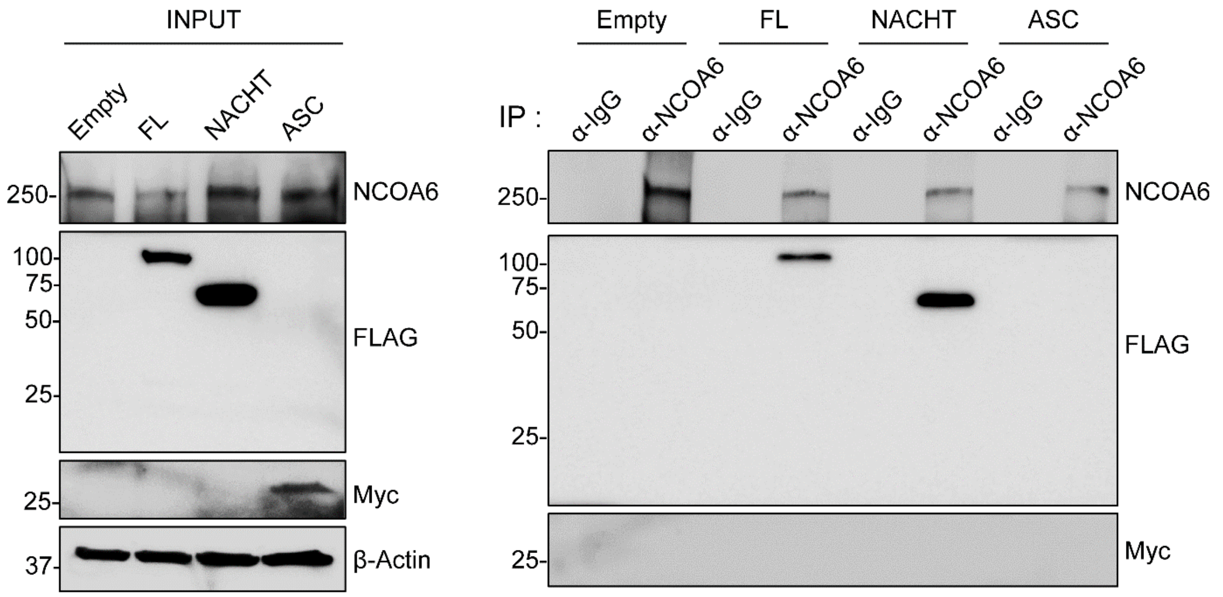

Supplementary Figure 13. Molecular interaction between NCOA6 and NACHT domain of NLRP3. Wild-type NLRP3, NACHT domain of NLRP3, and Myc-tagged ASC were overexpressed in HEK293T cells. Cell lysates were immunoprecipitated using anti-NCOA6 antibody (α-NCOA6) and then immunoblotted with the indicated antibodies: FL=full length. Isotype control IgG (α-NCOA6) was used as a control.

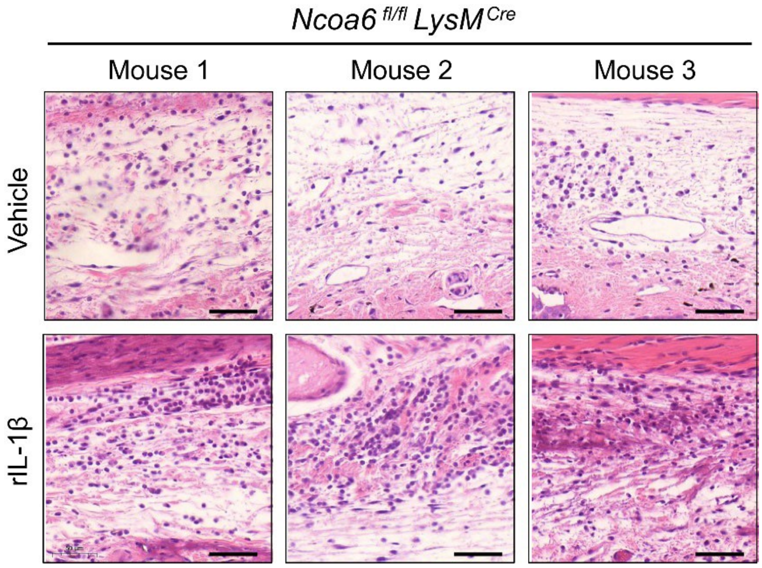

Supplementary Figure 14. Restoration of inflammatory cell infiltration in the affected joints of *Ncoa6*-KO mice after IL-1β injection. Images of H&E staining of footpads of *Ncoa6*$^{fl/fl}$*LysM*^{CRE} mice injected with vehicle or recombinant IL-1β near MSU crystals. Scale bars, 50 μm.

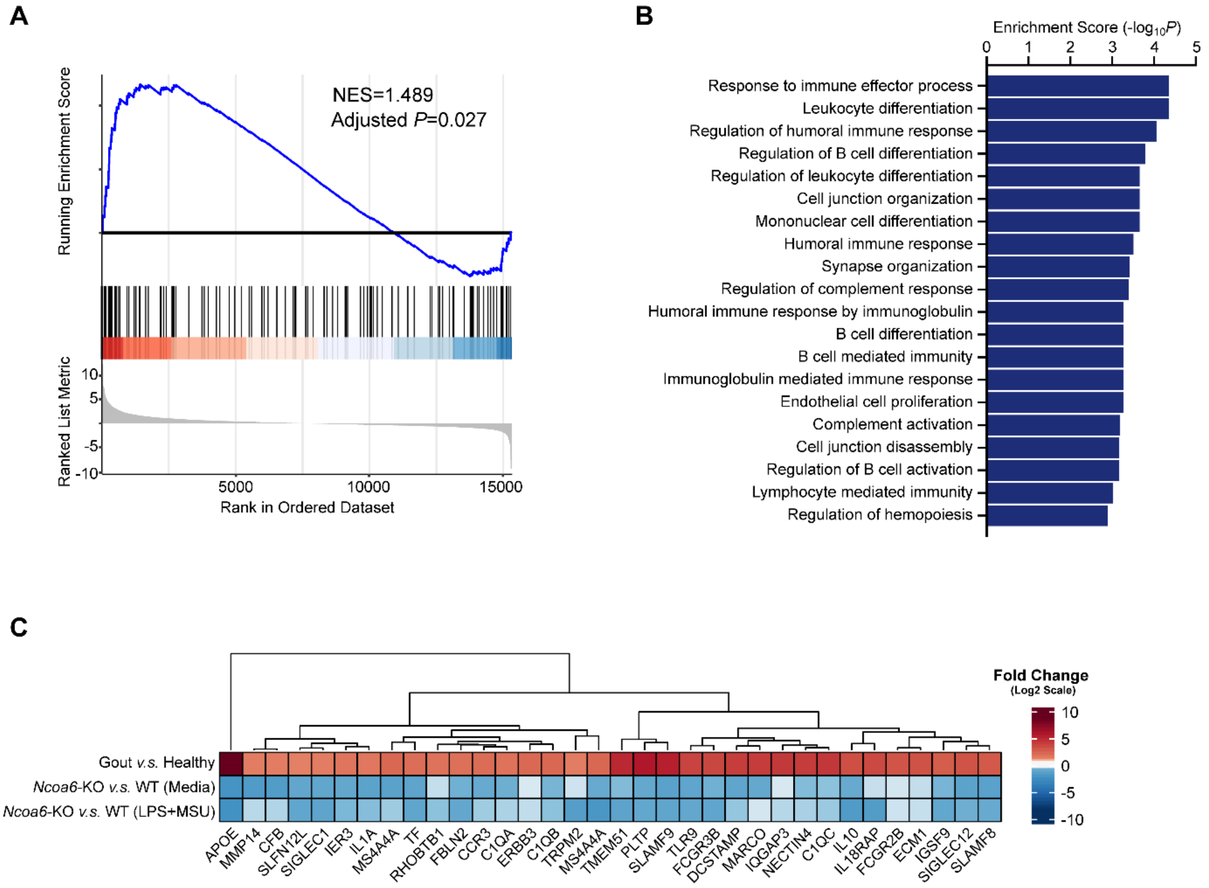

Supplementary Figure 15. Enrichment analysis of overlapping genes between DEGs in BMDMs of *Ncoa6*-KO mice and DEGs in macrophages of gouty arthritis patients. (A) GSEA plot showing that the set of downregulated DEGs in *Ncoa6*-KO macrophages was significantly enriched in macrophages of gout arthritis patients. NES=normalized enrichment score. **(B)** Top 20 GOBP terms enriched in the 33 overlapping genes between upregulated DEGs in the macrophages of gouty arthritis patients and downregulated DEGs in *Ncoa6*-KO macrophages stimulated with medium or LPS plus MSU. **(C)** Heatmap showing the fold changes in expression levels of the 33 overlapping genes between DEGs in the macrophages of *Ncoa6*-KO mice and DEGs in gouty arthritis patients.

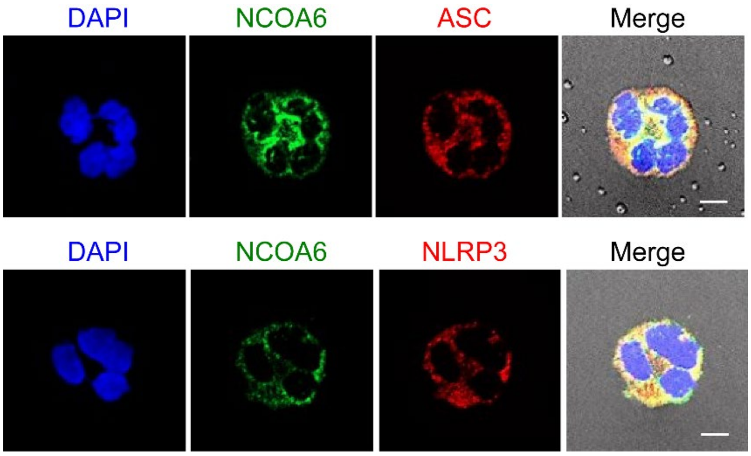

Supplementary Figure 16. Colocalization of NCOA6 with ASC and NLRP3 in polymorphonuclear leukocytes from a patient with gout. Polymorphonuclear cells were isolated from the synovial fluid of a patient with gouty arthritis. Representative immunocytochemistry images for NCOA6, ASC, and NLRP3 are shown. Scale bars, 10 μm.

Supplementary Table Legends

Supplementary Table 1. List of DEGs between *Ncoa6*-KO and *Ncoa6*-sufficient (WT) BMDMs in the absence (media alone) or presence of LPS plus MSU crystals (LPS+MSU). For each DEG, whether the DEG was up- (Up) or down-regulated (Down) under the two culture conditions of media alone and LPS+MSU stimulation is shown together with gene information (Entrez ID, gene symbol, and description) and statistics (Log_2 fold change value [Log_2FC], raw *P*-value, and adjusted *P*-value).

Supplementary Table 2. Gene Ontology Biological Processes (GOBPs) enriched by the six groups of DEGs in *Ncoa6*-KO BMDMs. The UX and DX group denotes up- and down-regulation only with media alone, XU and XD group up- and down-regulation only with LPS+MSU, and UU and DD group up- and down-regulation both with media alone and with LPS+MSU, respectively (*See* **Figure 2H**). For each group, GOBPs significantly (*P*-value < 0.05 and number of genes > 3) enriched by the DEGs are shown, together with the number of DEGs involved in the corresponding GOBPs (Count) and the enrichment *P*-values.

Supplementary Table 3. List of DEGs in the monocytes/macrophages of patients with gout (n=4) as compared to peripheral monocytes of age and sex-matched healthy controls (n=4). For each DEG, the ENSEMBL ID, Entrez ID, gene symbol, description, statistics, and log counts per million (CPM) values obtained from 'edgeR' are shown.

Supplementary Table 4. GOBP terms enriched by the 277 overlapping DEGs between DEGs in gout macrophages and those in *Ncoa6*-KO BMDMs (*See* **Figure 6B**). For the two culture conditions of media alone and stimulation with LPS+MSU; Gene Ontology

identification (ID); Gene Ontology Biological Process (GOBP) terms; gene symbol; the number of genes enriched (Count); and statistics regarding raw *P*-value, adjusted *P*-value (*P*.adjust), and enrichment score (-$\log_{10}$*P*.adjust) are presented.

Supplementary Table 5. GOBP terms enriched by the 33 DEGs downregulated in both medium- and LPS+MSU-stimulated *Ncoa6*-KO BMDMs. The 33 DEGs were significantly upregulated in gout macrophages, as determined by GSEA (*See* **Supplementary Figure 15**), and mostly represented innate immune-related cellular processes. Gene Ontology identification (ID); Gene Ontology Biological Process (GOBP) terms; gene symbol; the number of genes enriched (Count); and statistics regarding raw *P*-value, adjusted *P*-value (*P*.adjust), and enrichment score (-$\log_{10}$*P*.adjust) are shown.

Supplementary Video Legends

Supplementary Video 1. Z-stacking image of a 3D image rotated in the X-axis of PMA-stimulated THP-1 cells activated inflammasome. Representative captured image from this video is presented in Supplementary Figure 5B.

Supplementary Video 2. Z-stacking image of a 3D image rotated in the Y-axis of PMA-stimulated THP-1 cells activated inflammasome. Representative captured image from this video is presented in Supplementary Figure 5B.
