# Supplementary material for: Nuclear receptor coactivator 6 is a critical regulator of NLRP3 inflammasome activation and gouty arthritis
Source: Cell Mol Immunol. 2024 Jan 10;21(3):227–44. doi: 10.1038/s41423-023-01121-x (PMC10902316; doi:10.1038/s41423-023-01121-x)

Figure 1B

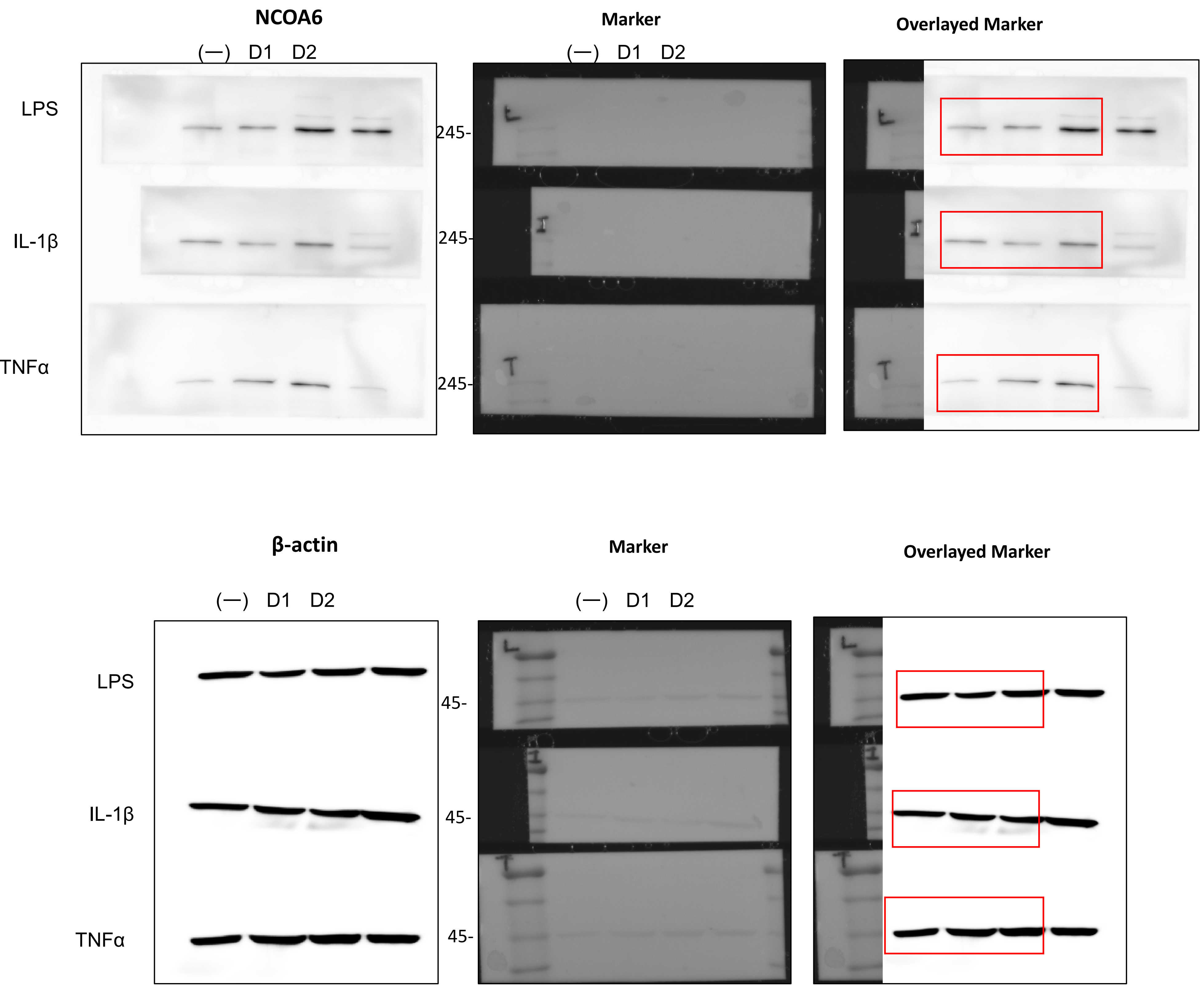

Figure 2C

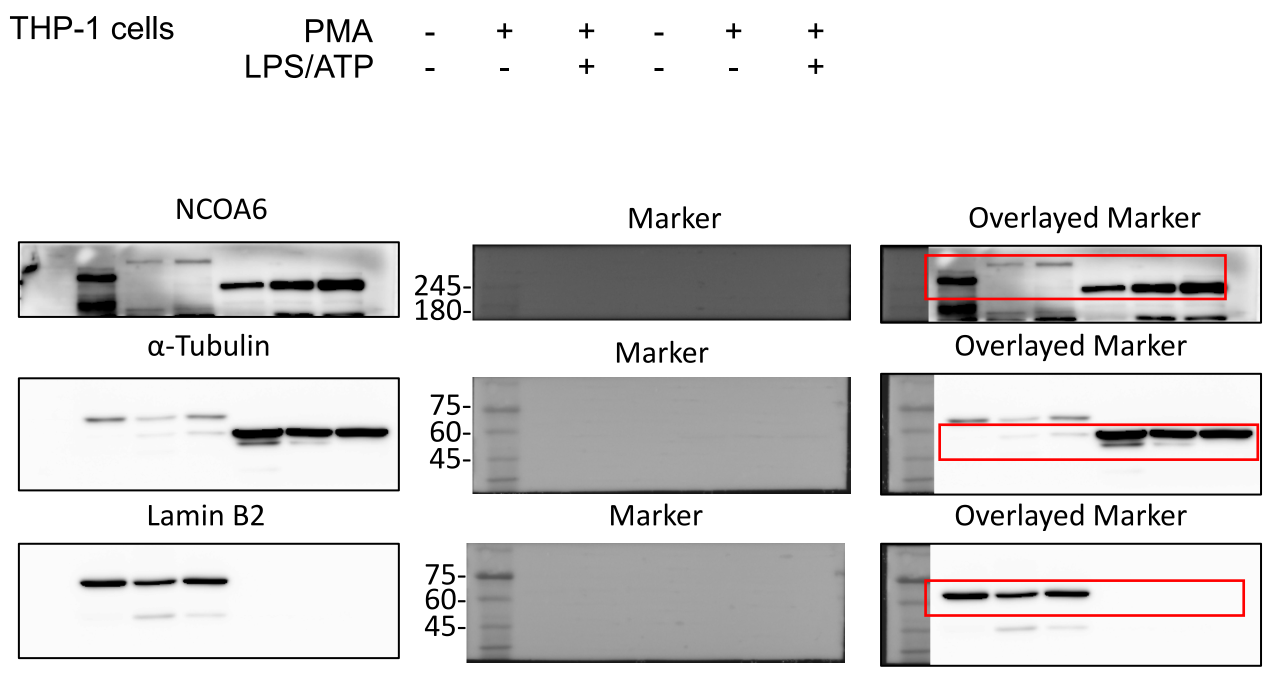

Figure 3A

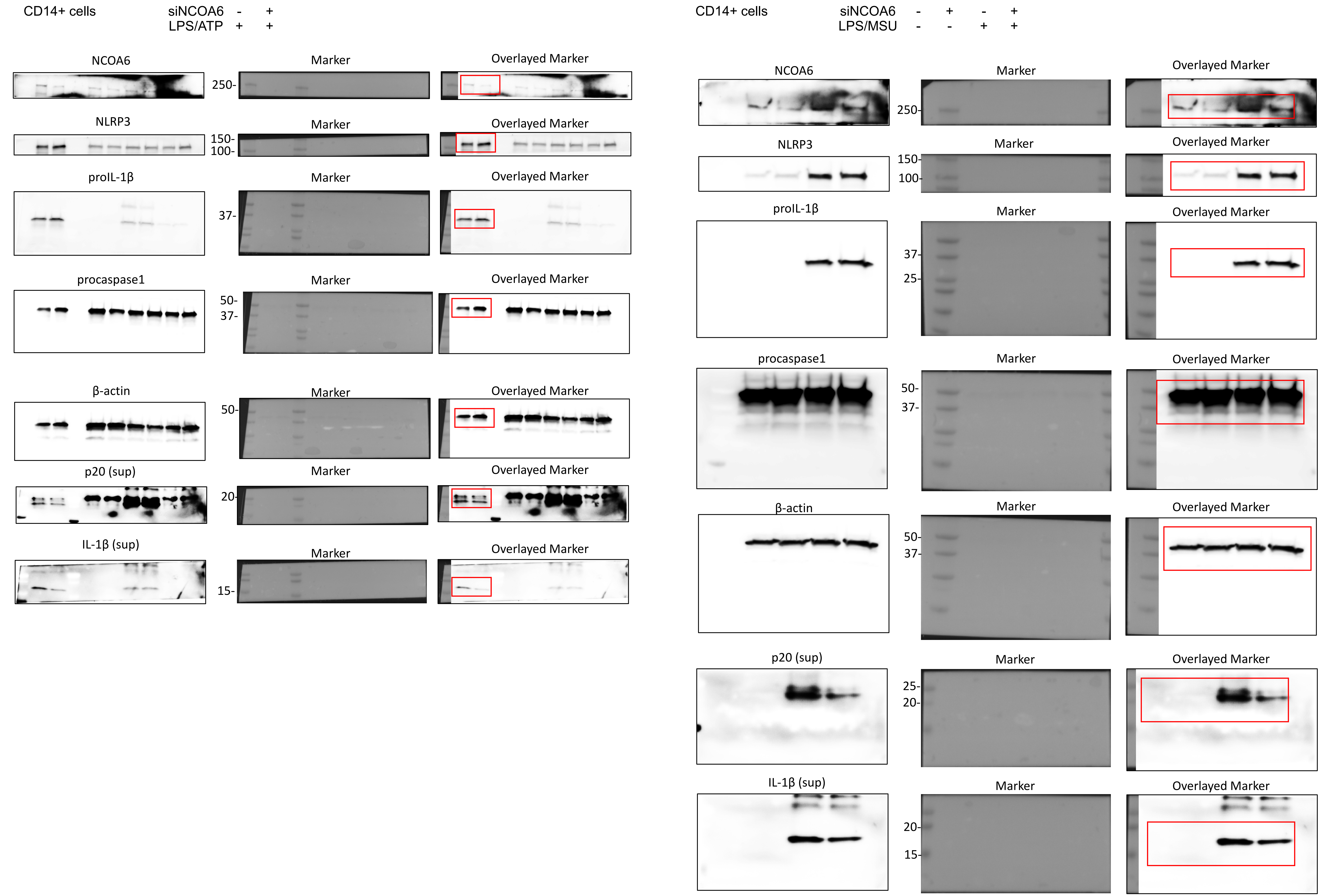
CD14+ cells
siNCOA6 - +
LPS/ATP + +
NCOA6
Marker
Overlayed Marker
250-
NLRP3
150-
100-
proIL-1β
37-
procaspase1
50-
37-
β-actin
50-
p20 (sup)
20-
IL-1β (sup)
15-
CD14+ cells
siNCOA6 - + - +
LPS/MSU - - + +
NCOA6
Marker
Overlayed Marker
250-
NLRP3
150-
100-
proIL-1β
37-
25-
procaspase1
50-
37-
β-actin
50-
37-
p20 (sup)
25-
20-
IL-1β (sup)
20-
15-

Figure 3D

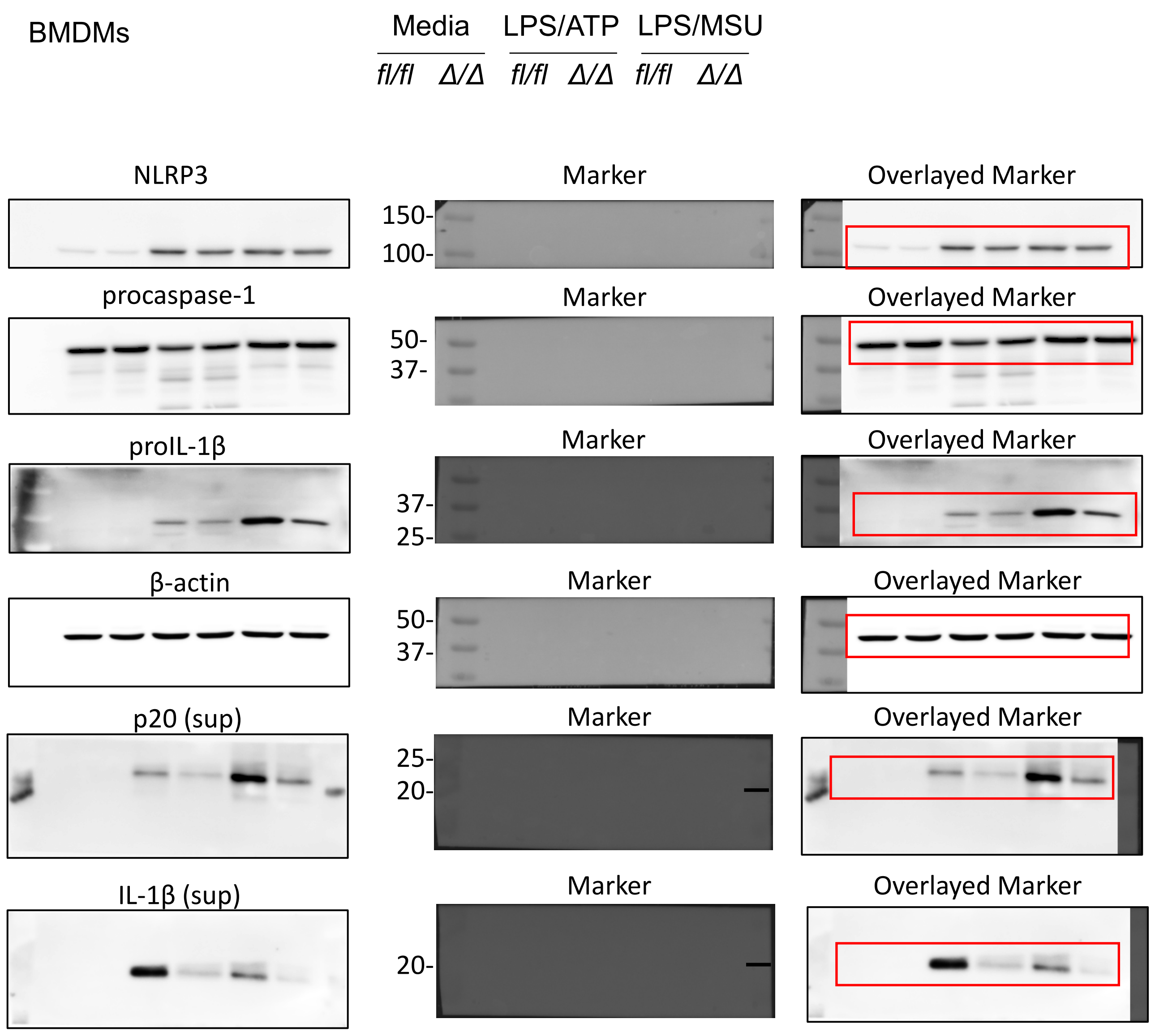
BMDMs
Media
LPS/ATP
LPS/MSU
fl/fl
Δ/Δ
fl/fl
Δ/Δ
fl/fl
Δ/Δ
NLRP3
Marker
Overlayed Marker
150-
100-
procaspase-1
Marker
Overlayed Marker
50-
37-
proIL-1β
Marker
Overlayed Marker
37-
25-
β-actin
Marker
Overlayed Marker
50-
37-
p20 (sup)
Marker
Overlayed Marker
25-
20-
IL-1β (sup)
Marker
Overlayed Marker
20-

Figure 3G

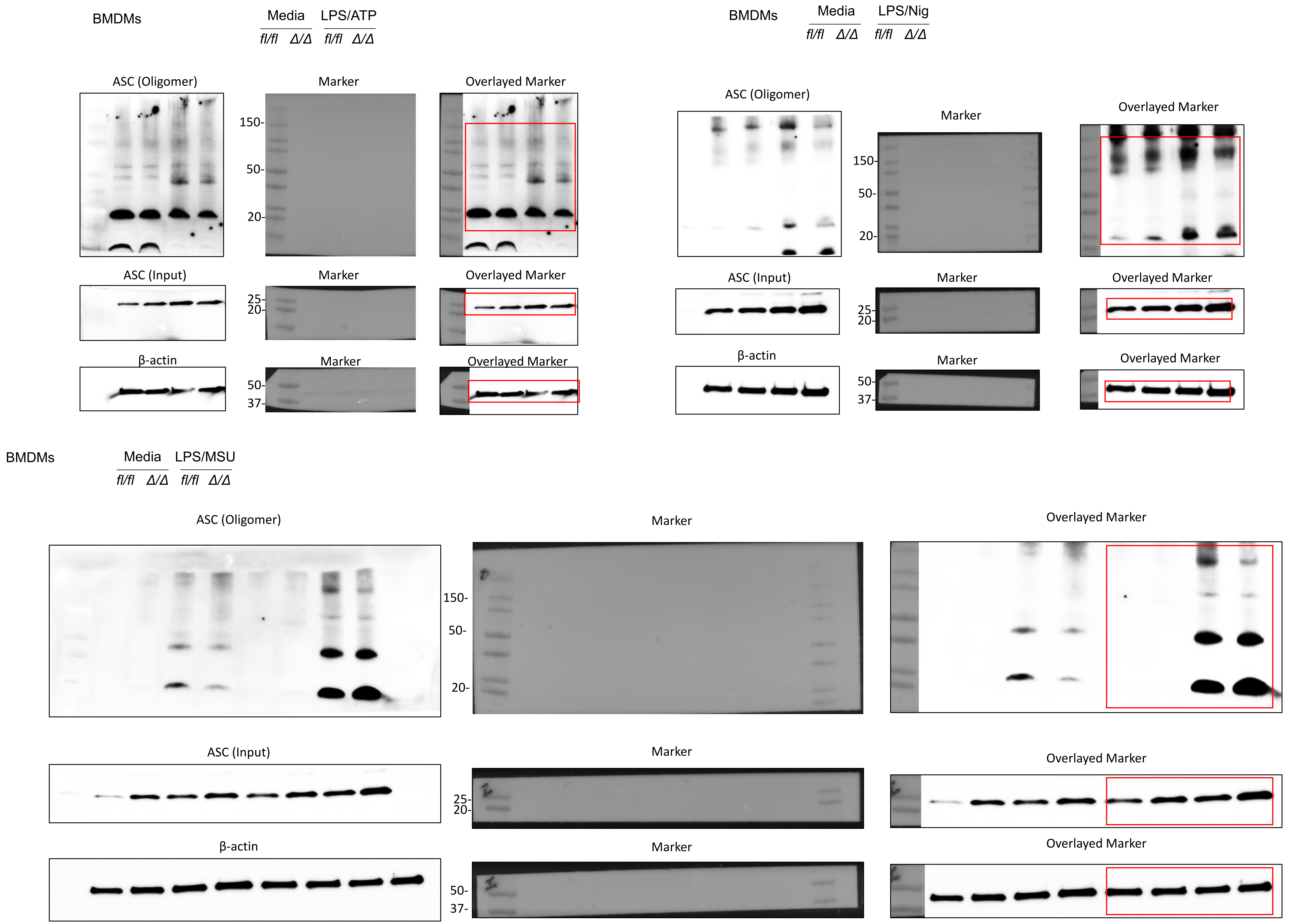

BMDMs
Media
LPS/ATP
fl/fl
Δ/Δ
fl/fl
Δ/Δ
ASC (Oligomer)
Marker
Overlayed Marker
150-
50-
20-
ASC (Input)
Marker
Overlayed Marker
25-
20-
β-actin
Marker
Overlayed Marker
50-
37-
BMDMs
Media
LPS/Nig
fl/fl
Δ/Δ
fl/fl
Δ/Δ
ASC (Oligomer)
Marker
Overlayed Marker
150-
50-
20-
ASC (Input)
Marker
Overlayed Marker
25-
20-
β-actin
Marker
Overlayed Marker
50-
37-
BMDMs
Media
LPS/MSU
fl/fl
Δ/Δ
fl/fl
Δ/Δ
ASC (Oligomer)
Marker
Overlayed Marker
150-
50-
20-
ASC (Input)
Marker
Overlayed Marker
25-
20-
β-actin
Marker
Overlayed Marker
50-
37-

Figure 3H

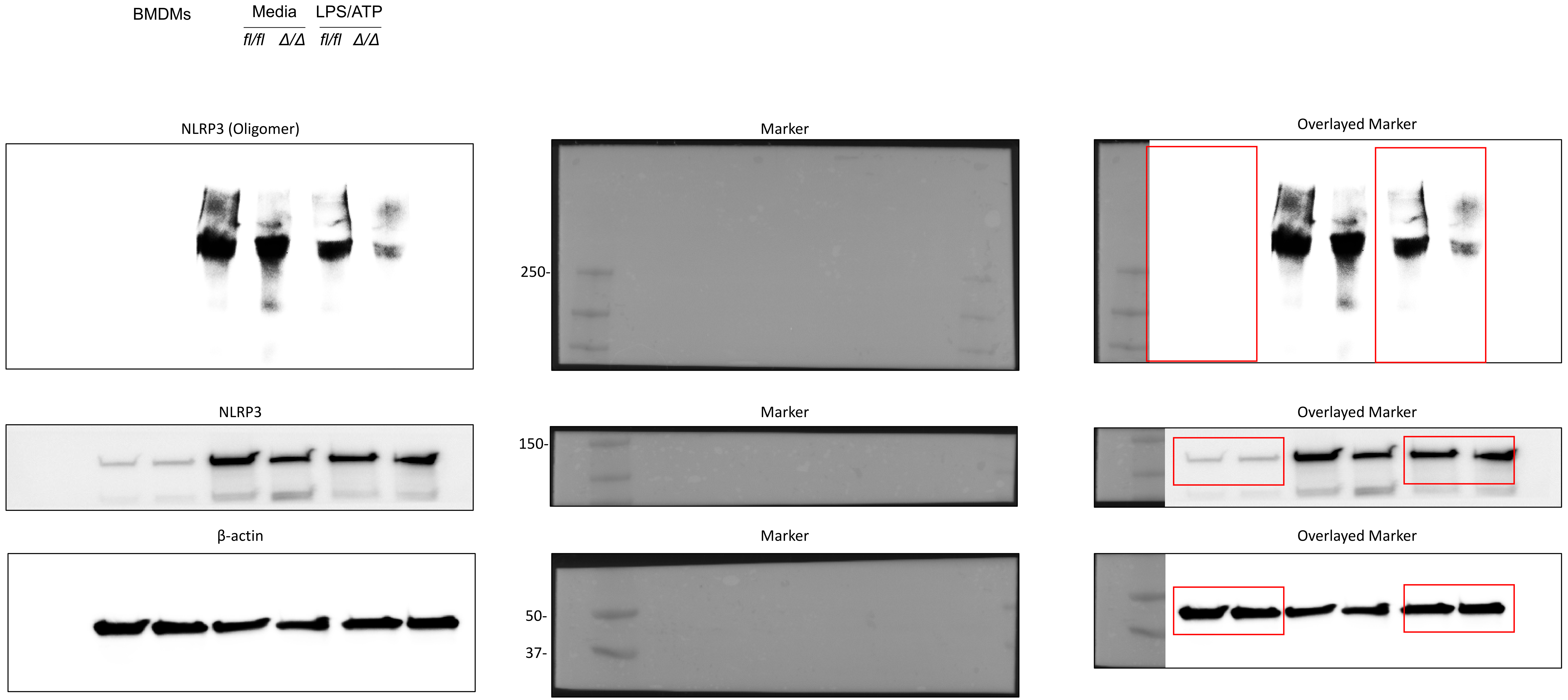
BMDMs
Media
LPS/ATP
fl/fl Δ/Δ fl/fl Δ/Δ
NLRP3 (Oligomer)
Marker
250-
Overlayed Marker
NLRP3
Marker
150-
Overlayed Marker
β-actin
Marker
50-
37-
Overlayed Marker

Figure 4A

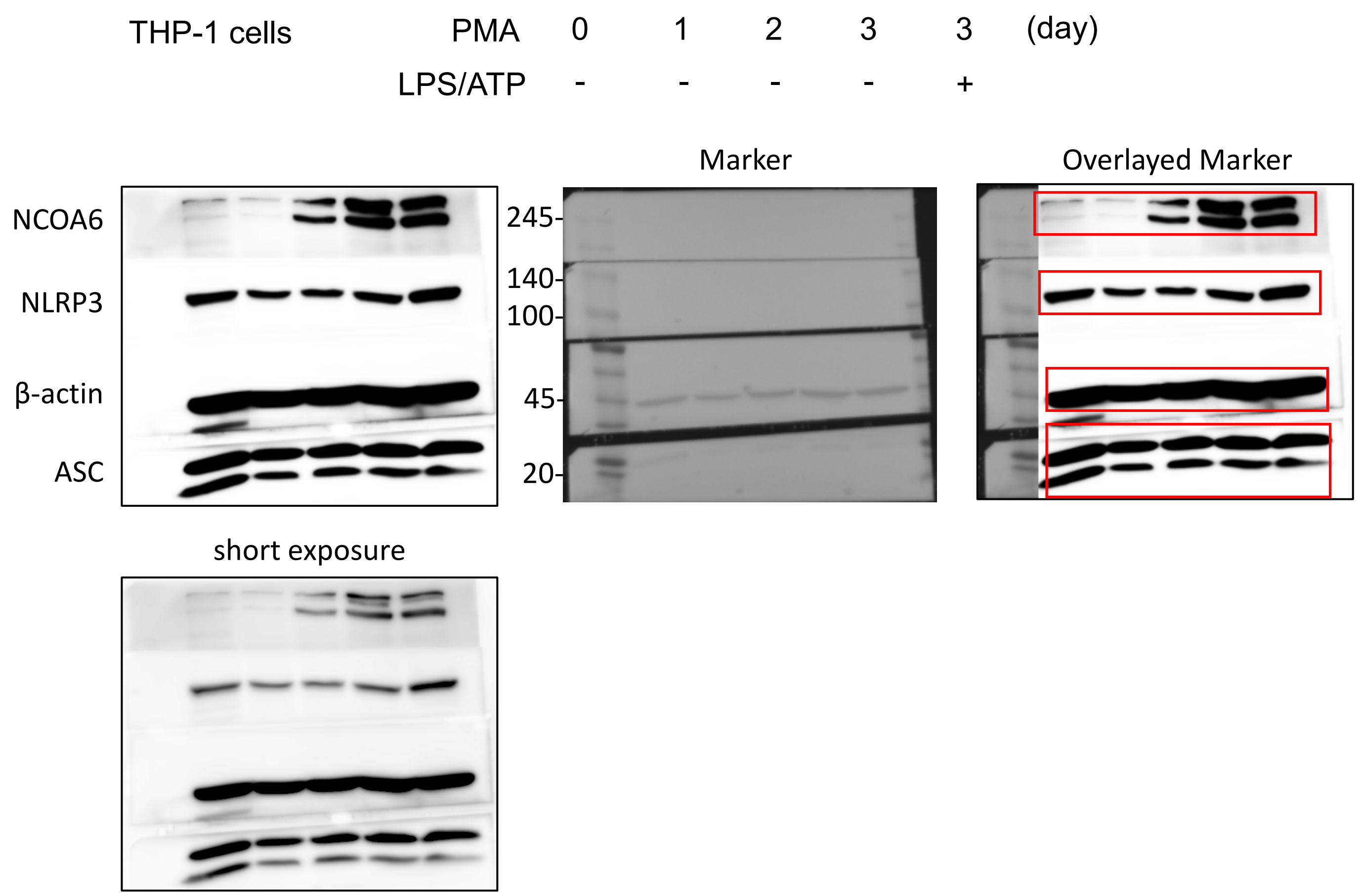

Figure 4B

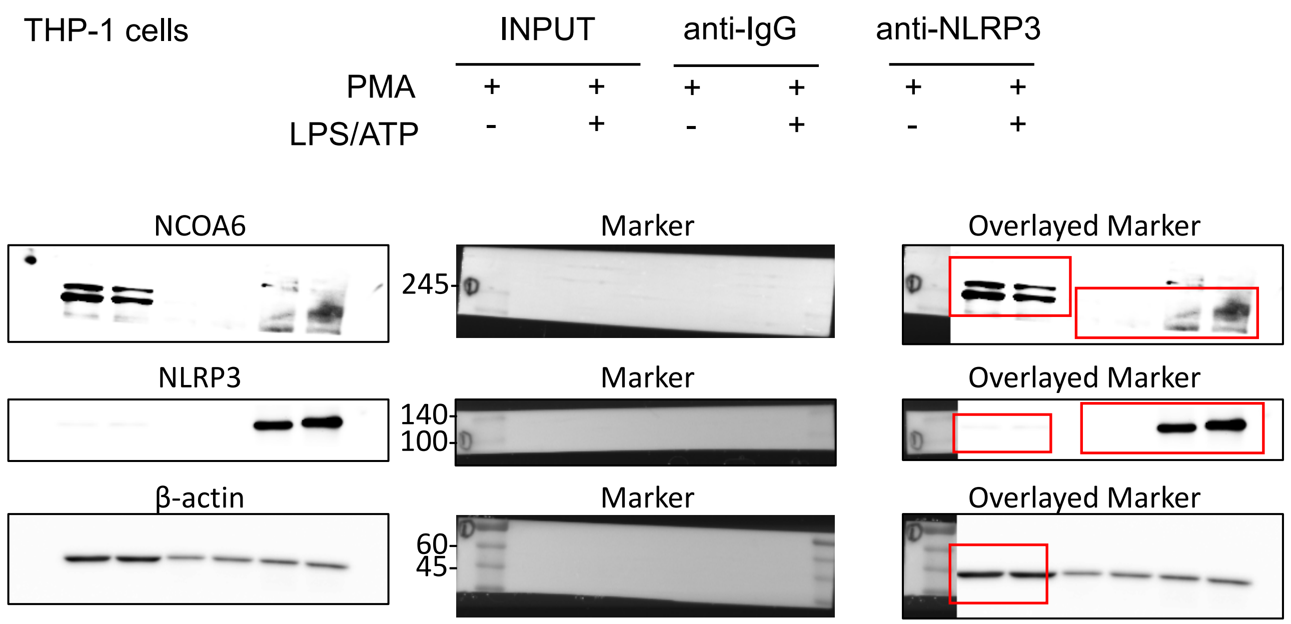

Figure 4E

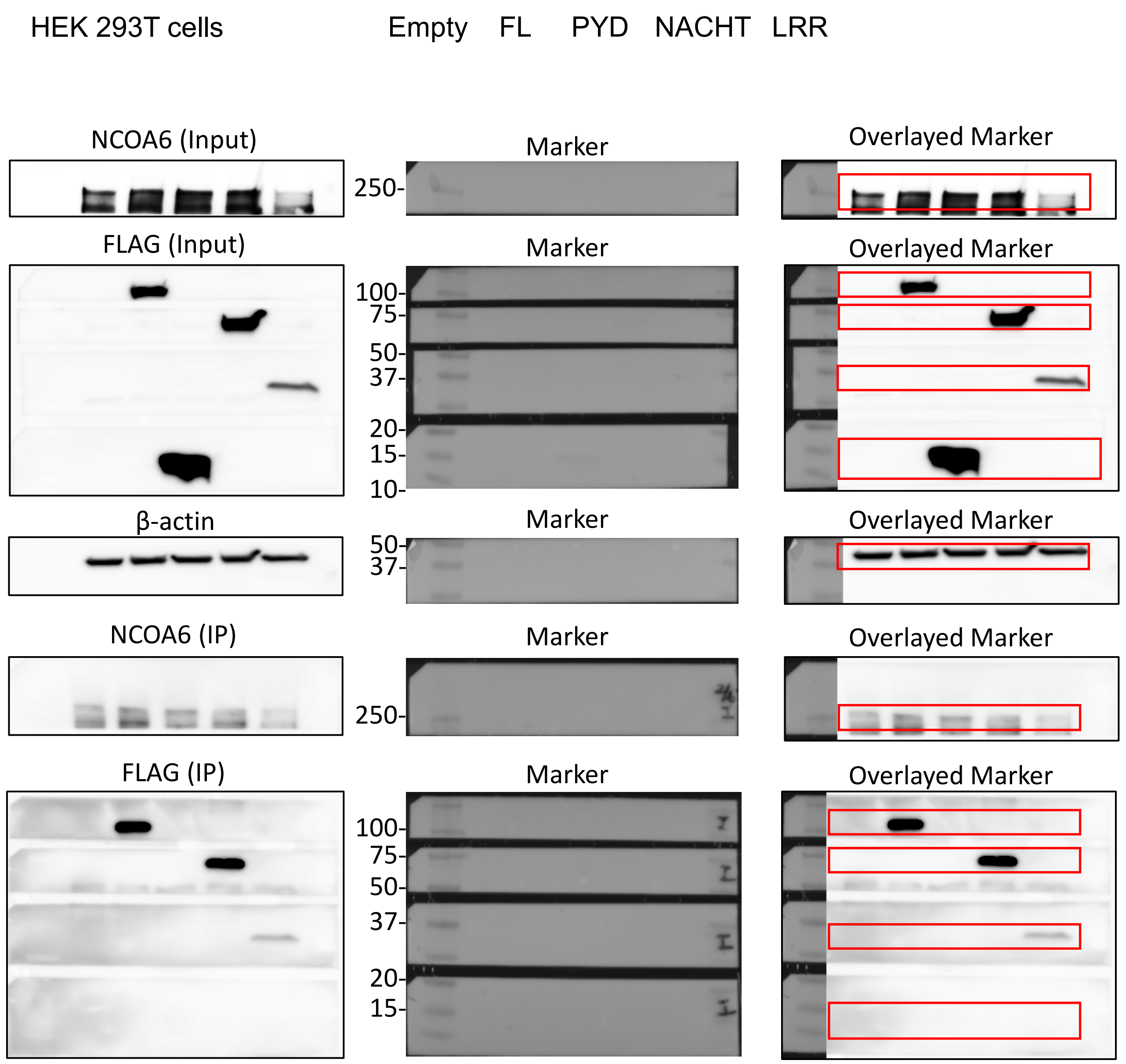

Figure 4F

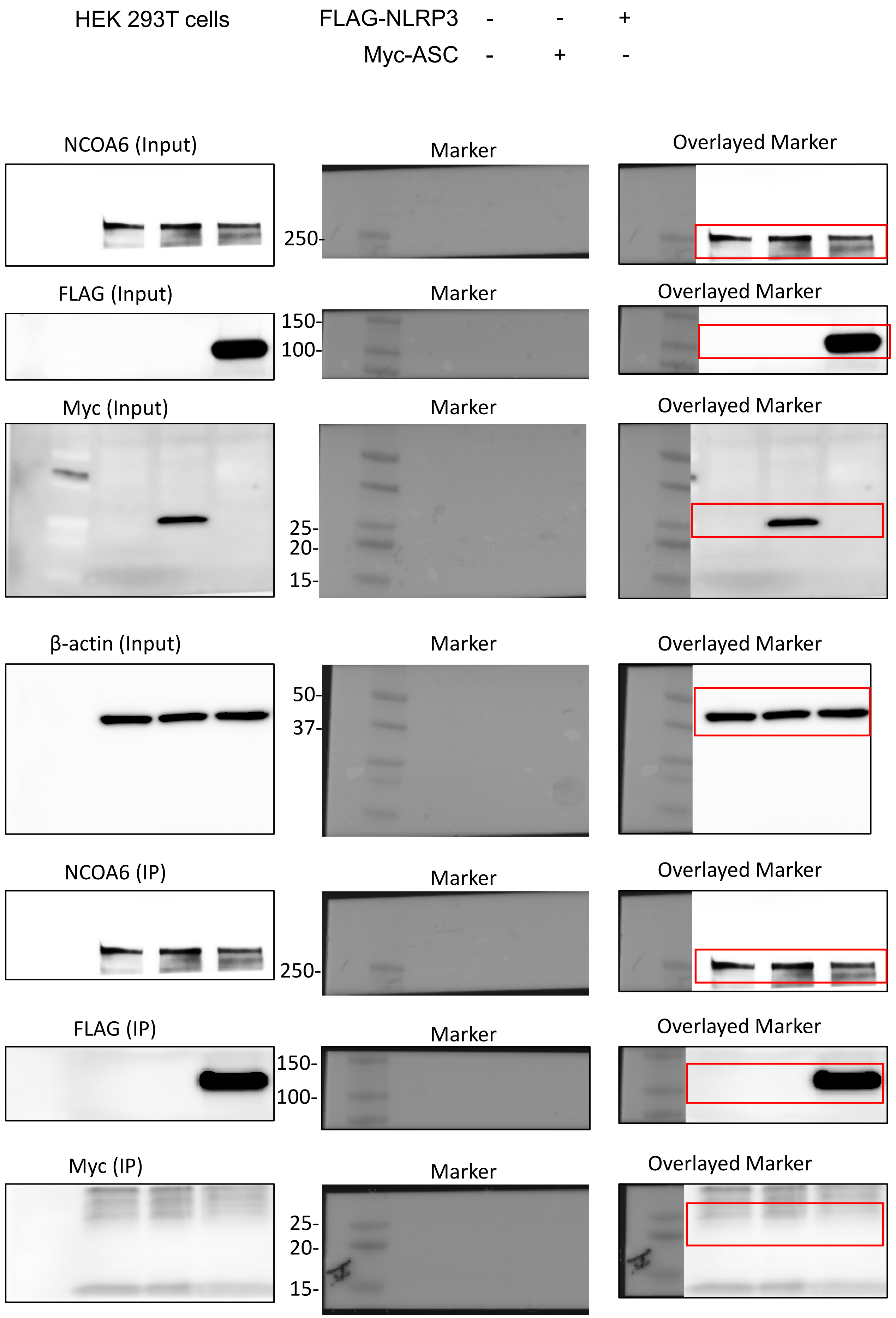

Figure 4G

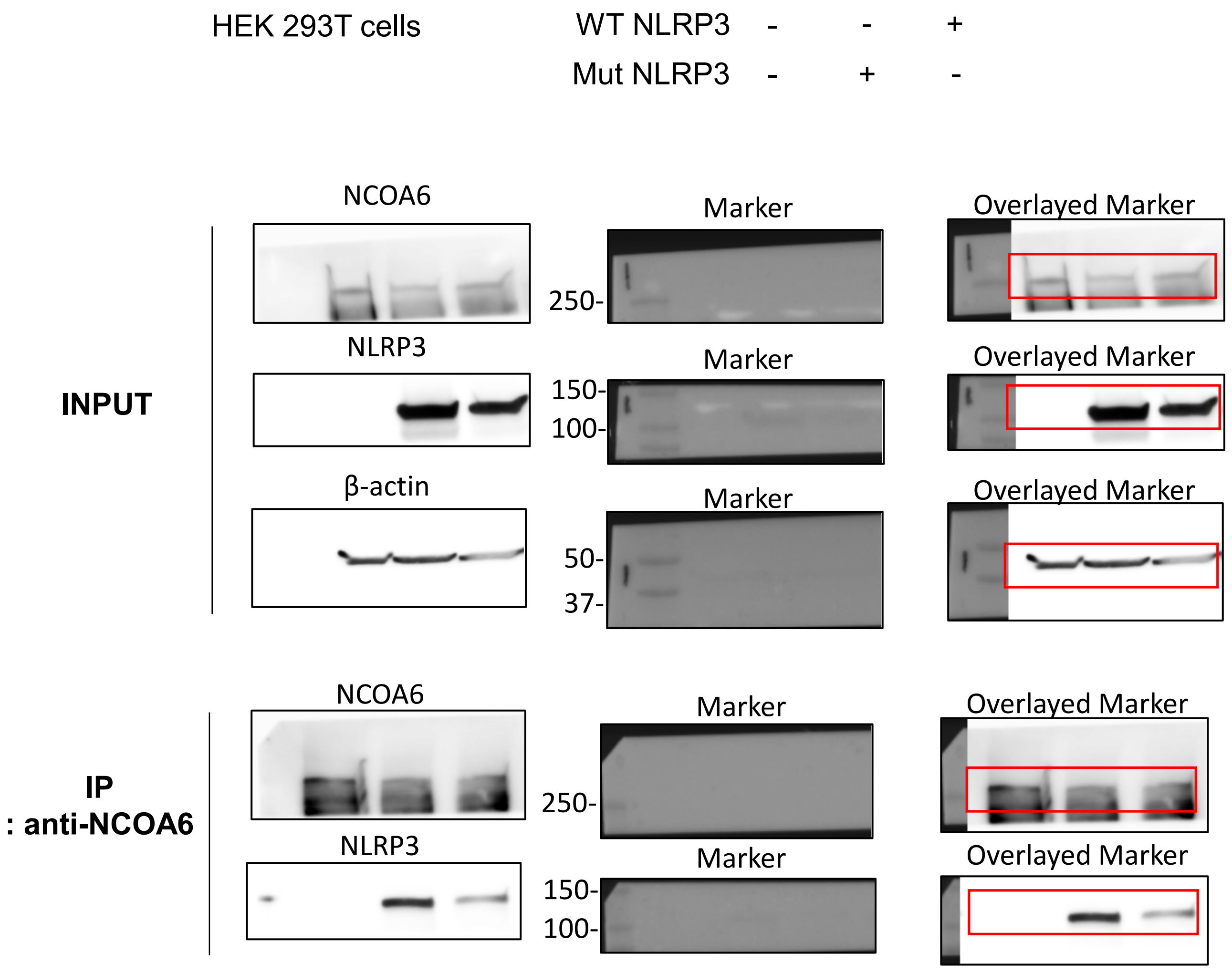

Figure 4H

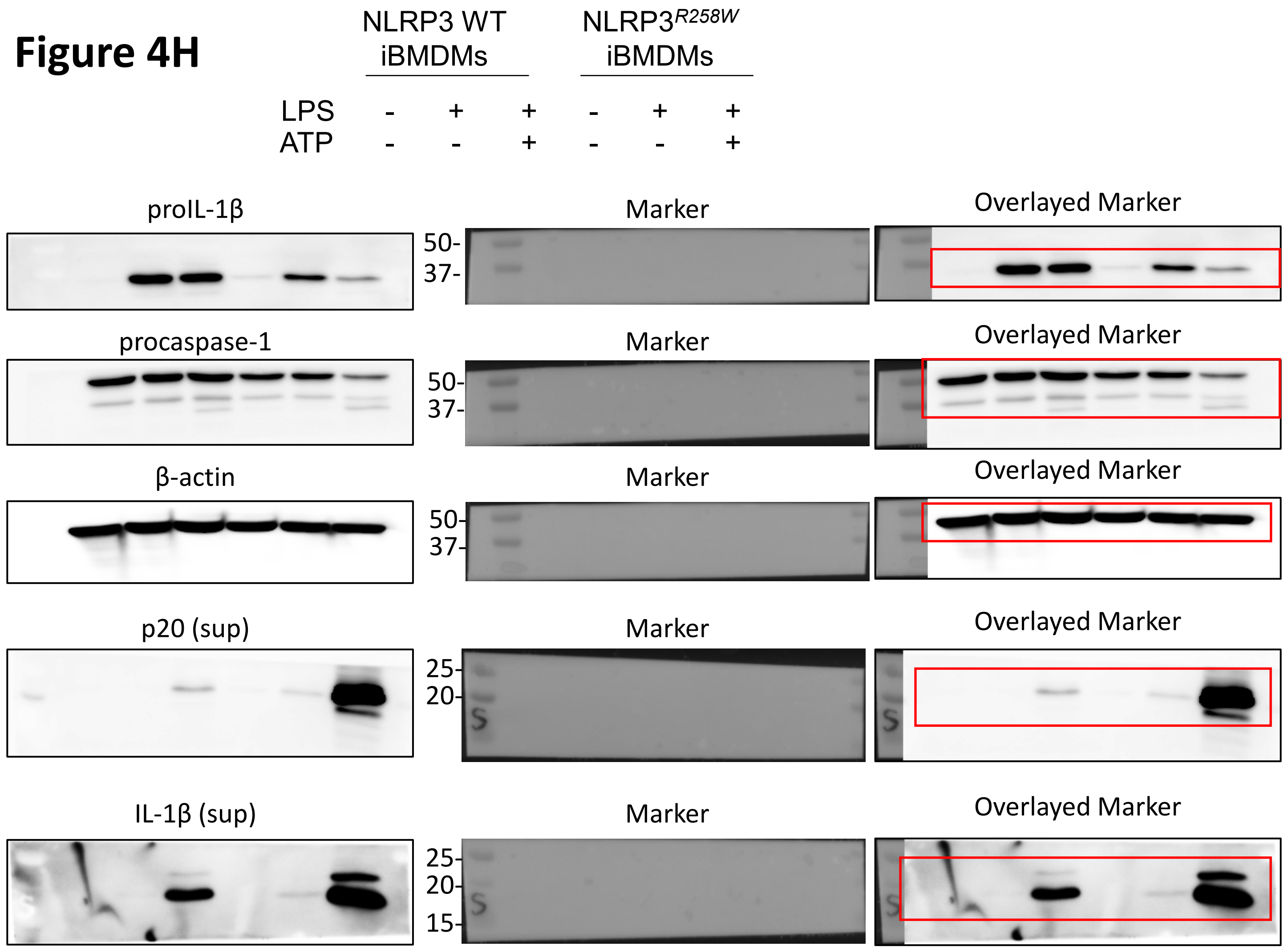

Figure 4I

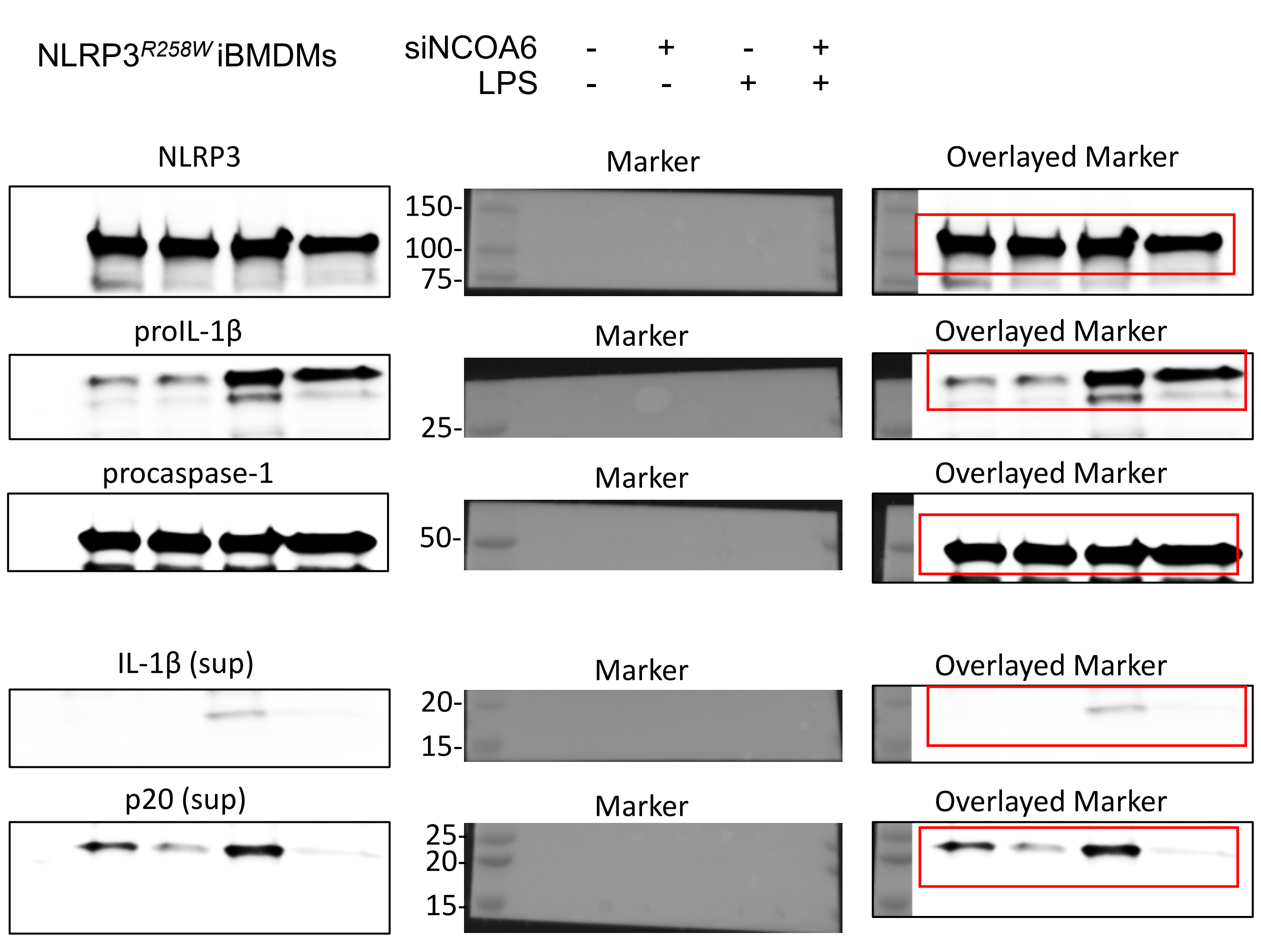

Figure 4K

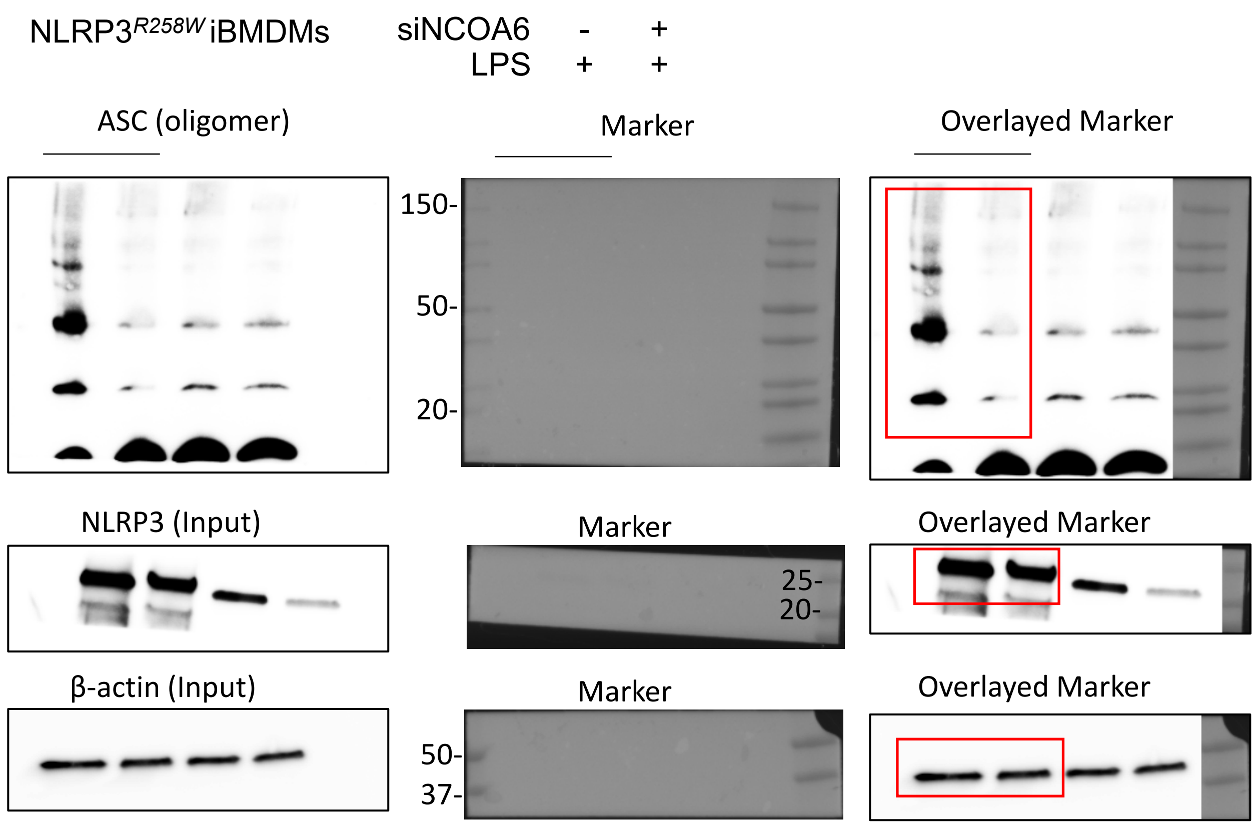

Figure 5D

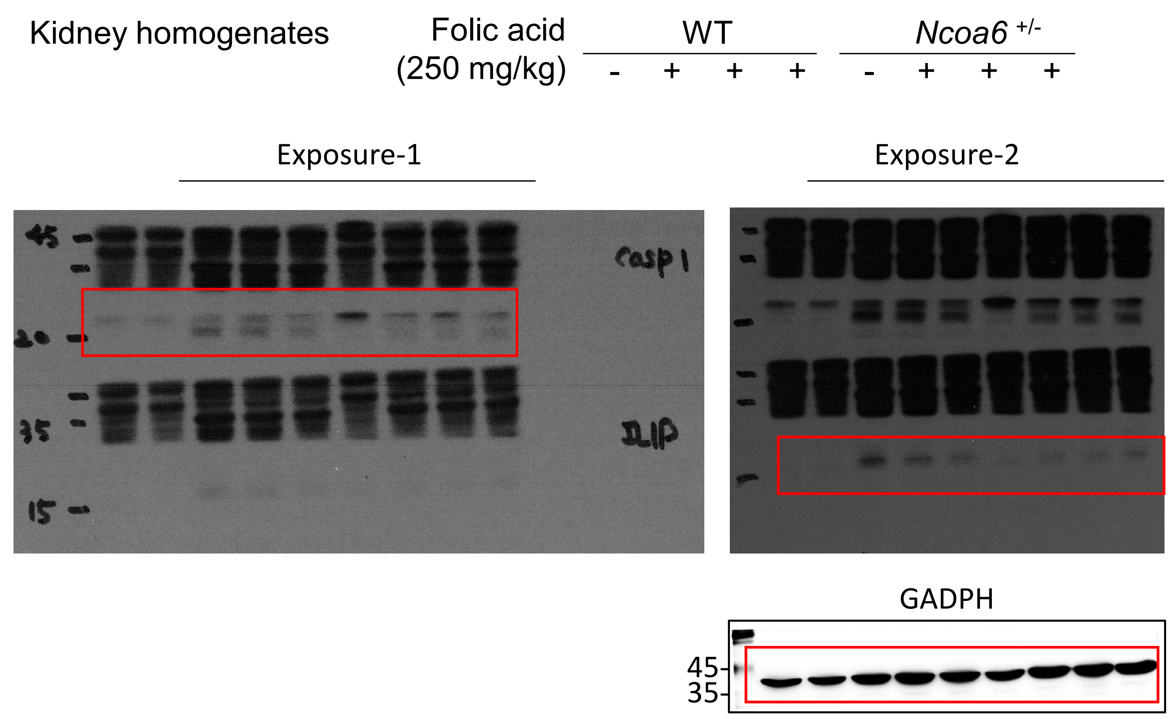

Figure 5J

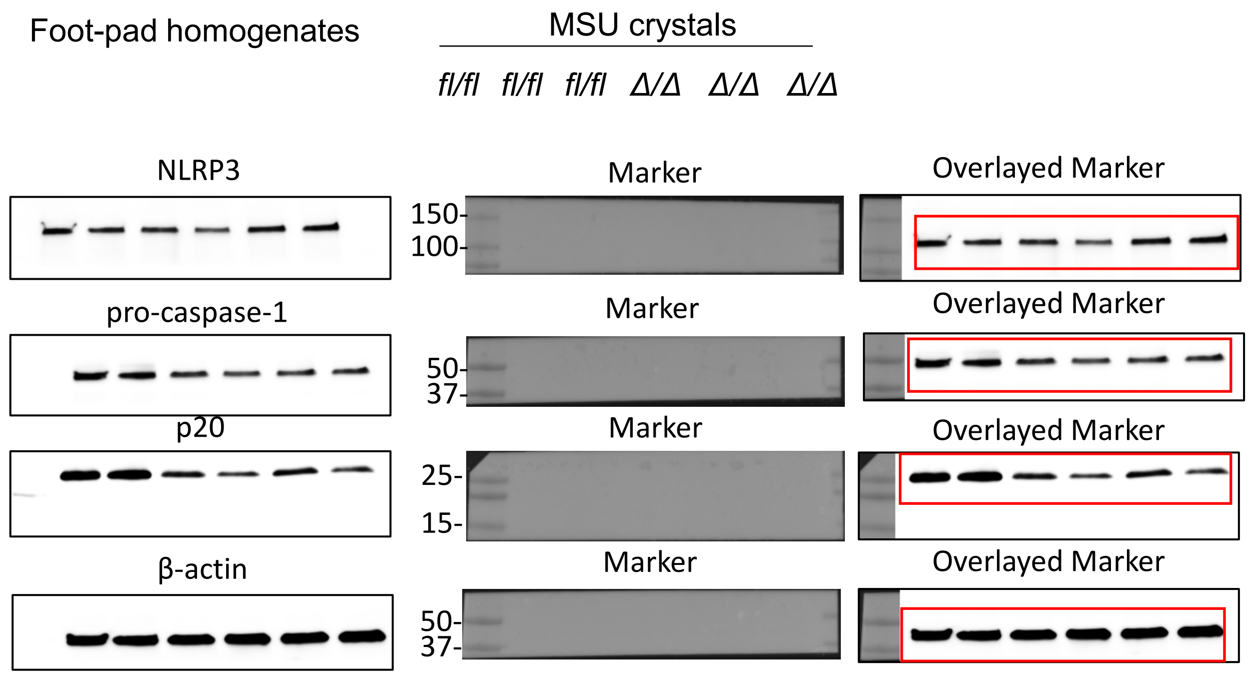

Figure 6J

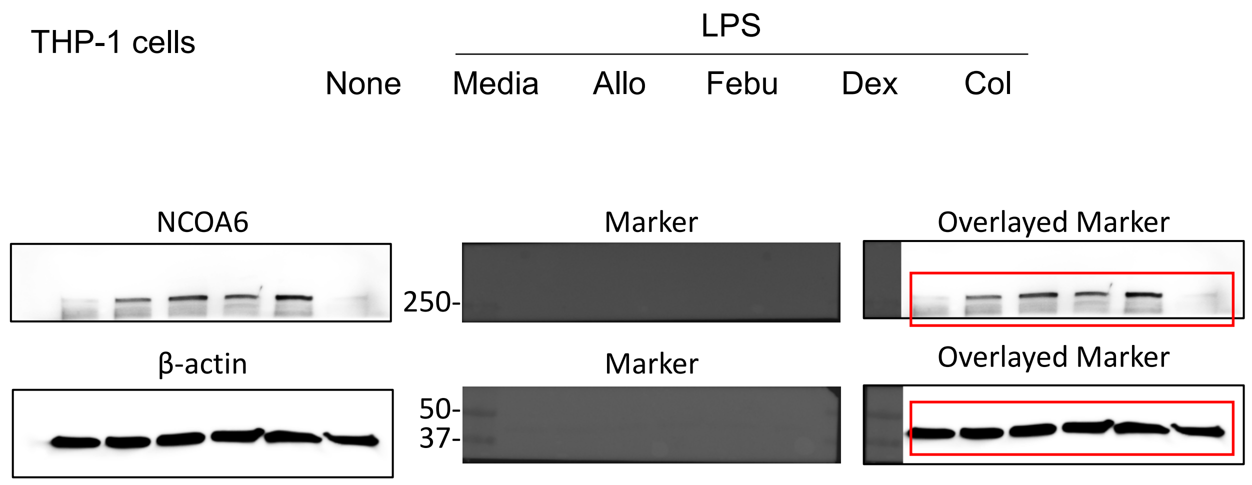

Supplementary Figure 2A

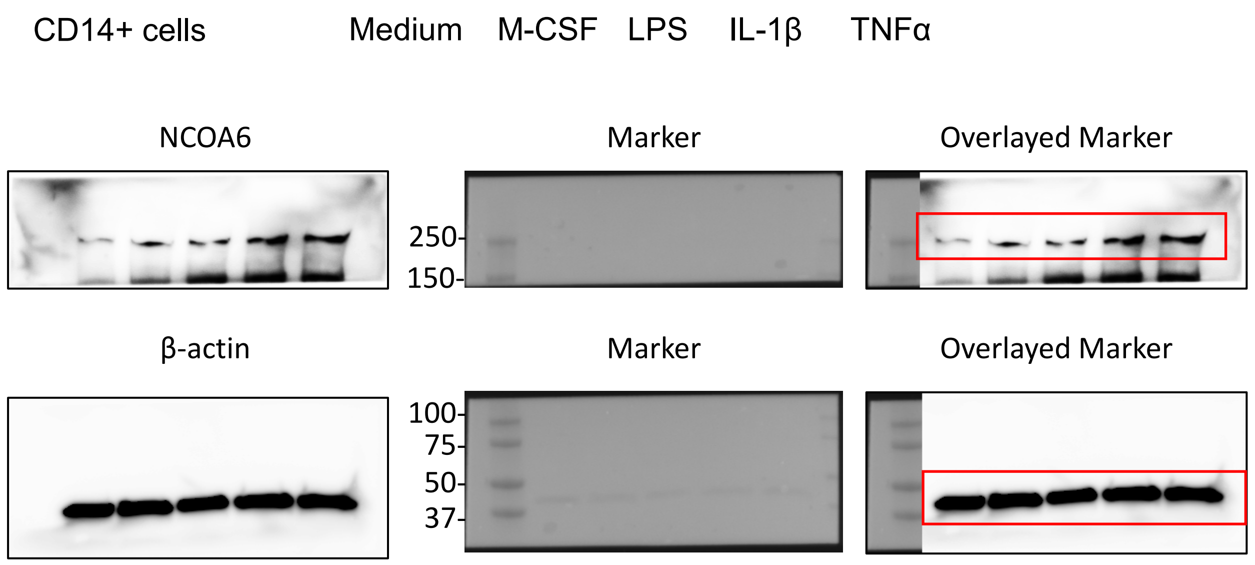

Supplementary Figure 3 (gel)

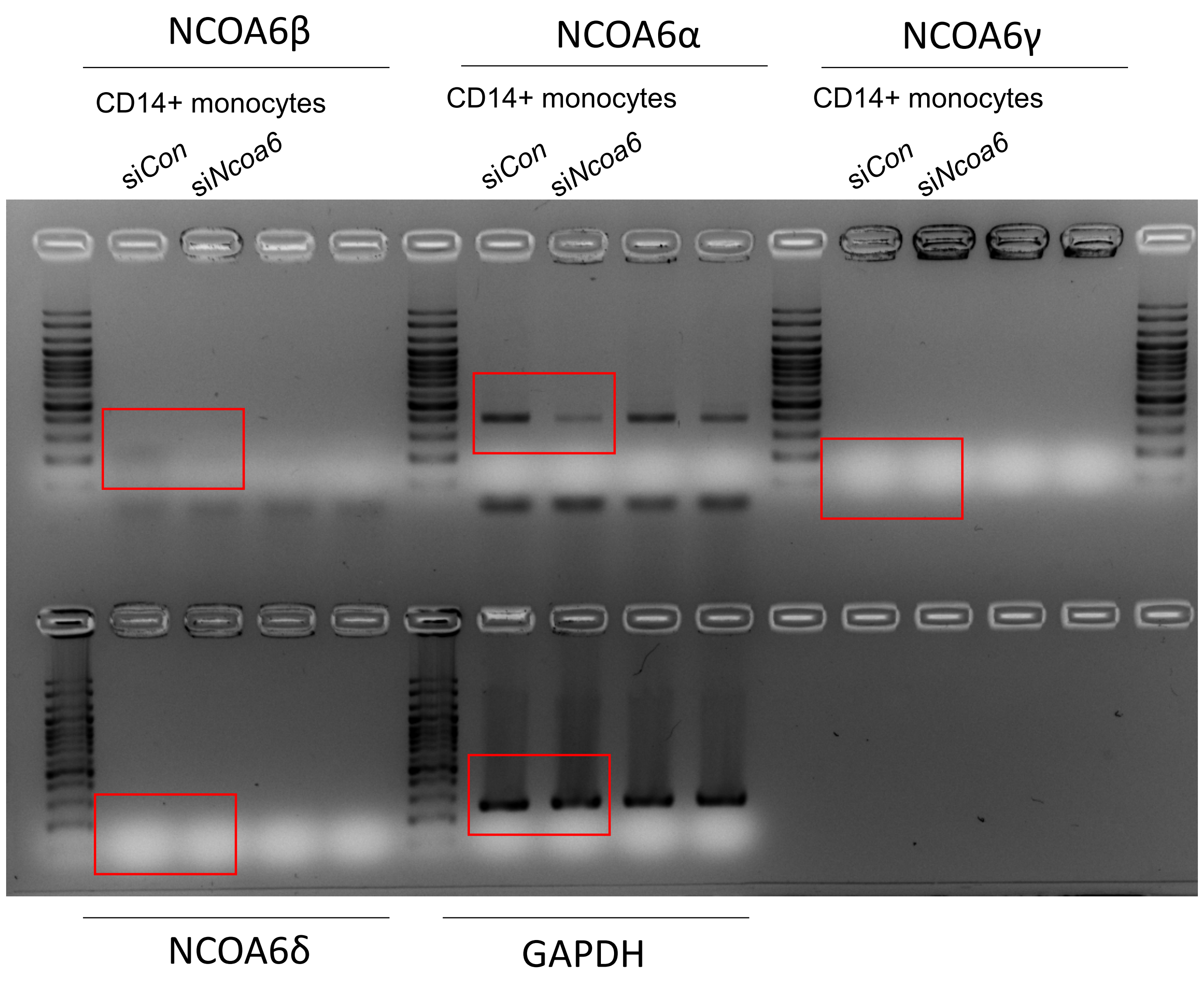

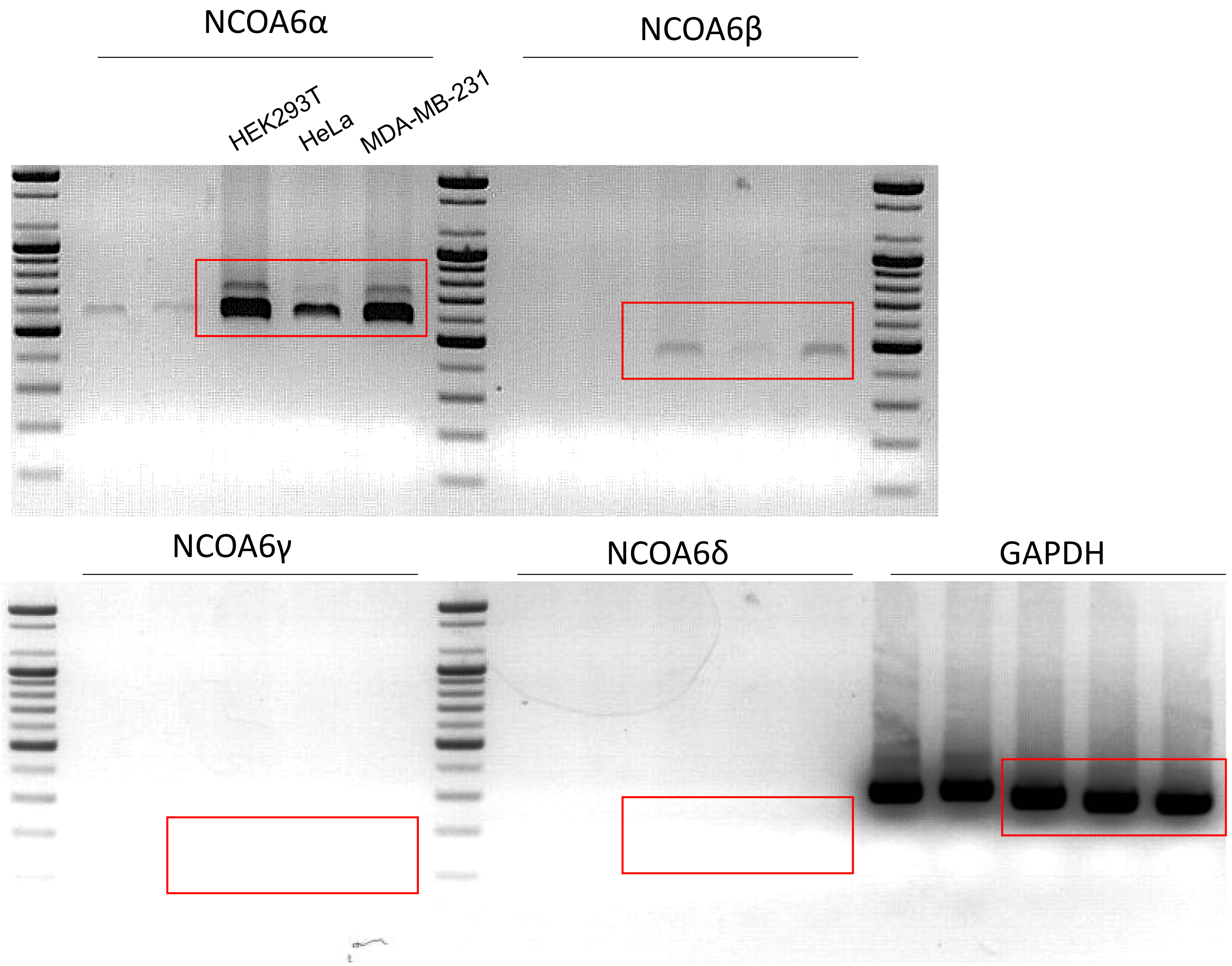

Supplementary Figure 6B (gel)

Genotyping of genomic DNA extracted from tail tissues of *Ncoa6*$^{fl/fl}$ mice and *Ncoa6*$^{fl/fl}$*LysM*^{CRE} mice.

PCR analysis using *flox* primer

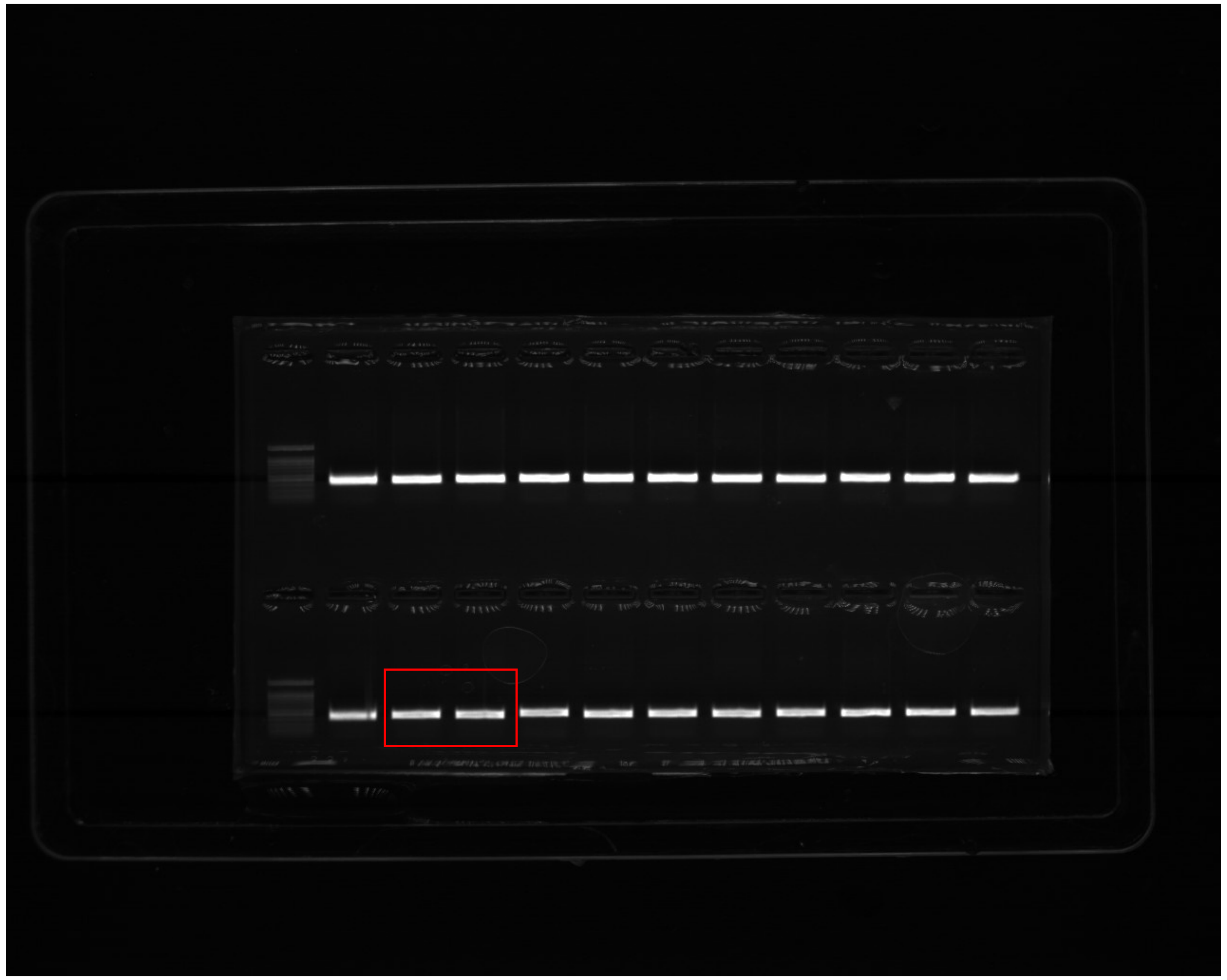

PCR analysis using *cre* primer

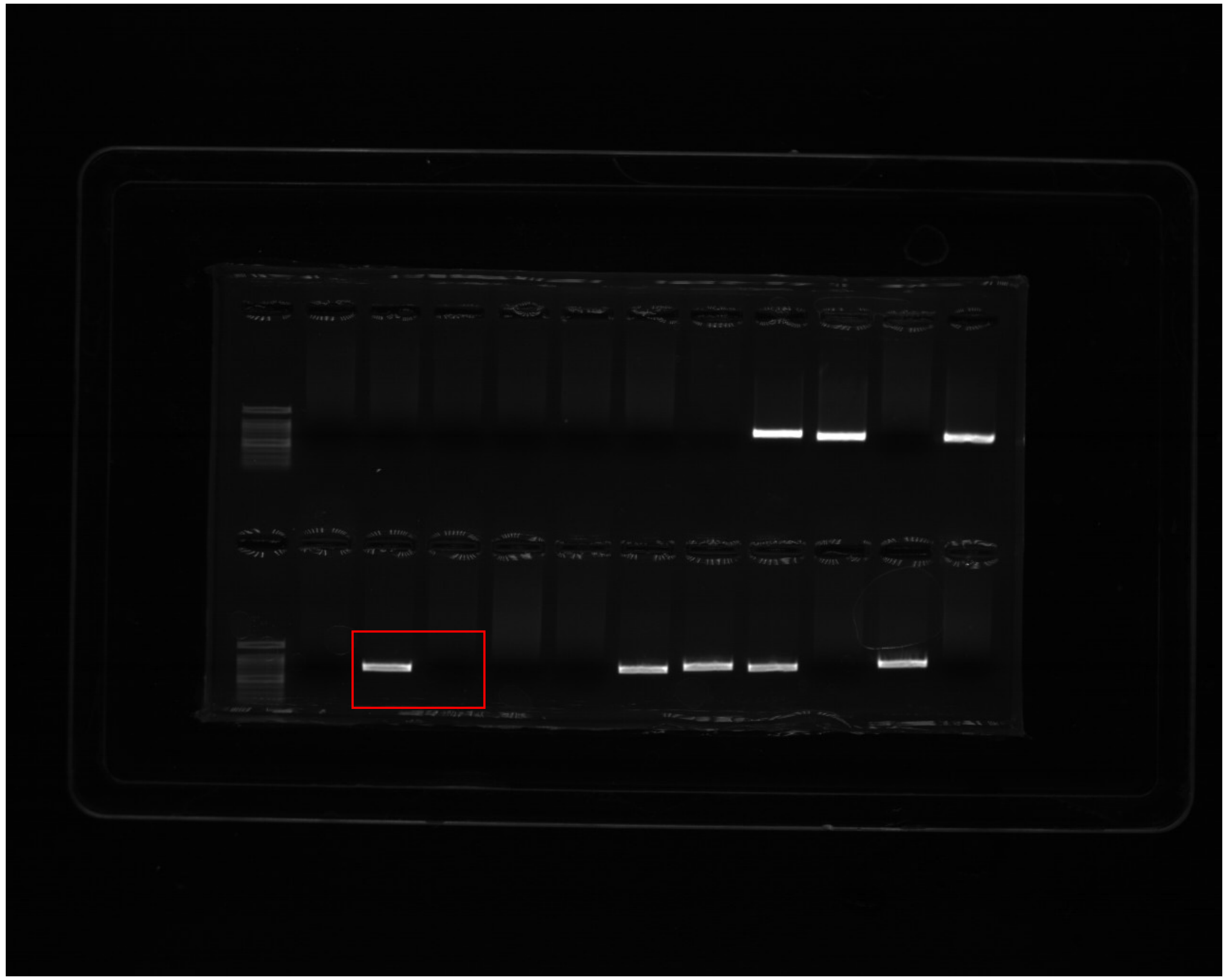

Supplementary Figure 10

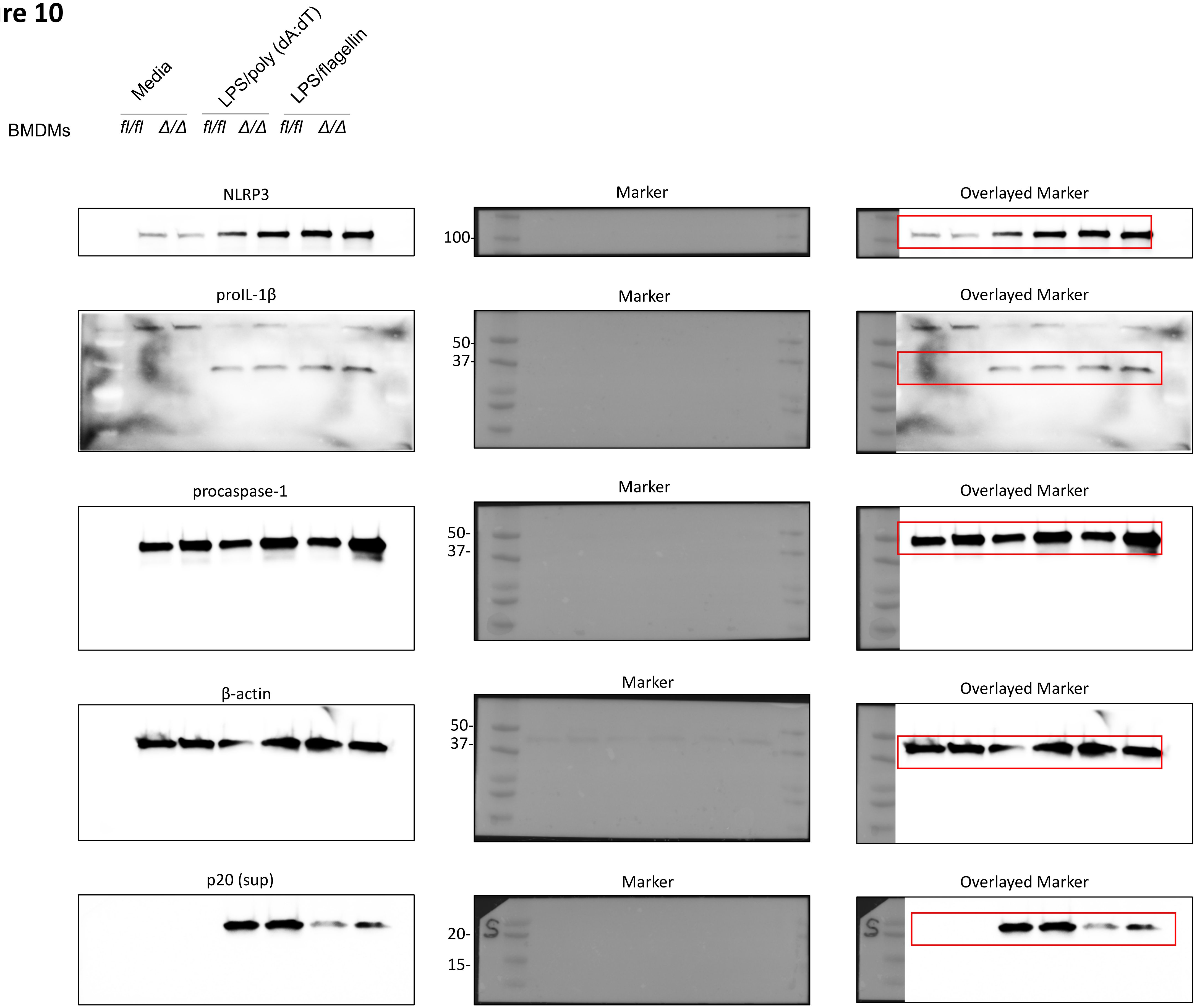

Supplementary Figure 11A & Supplementary Figure 11B

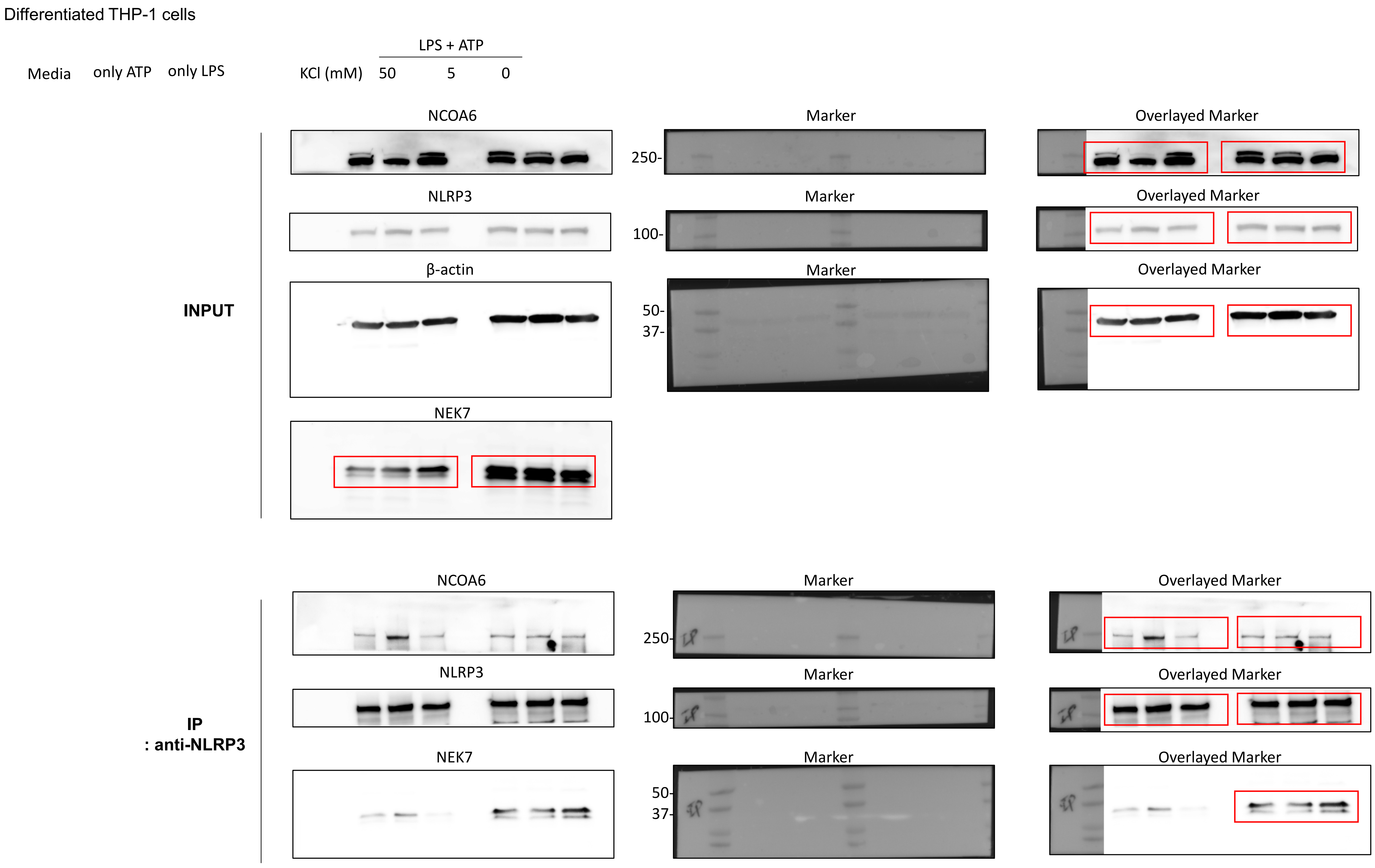

Supplementary Figure 11D

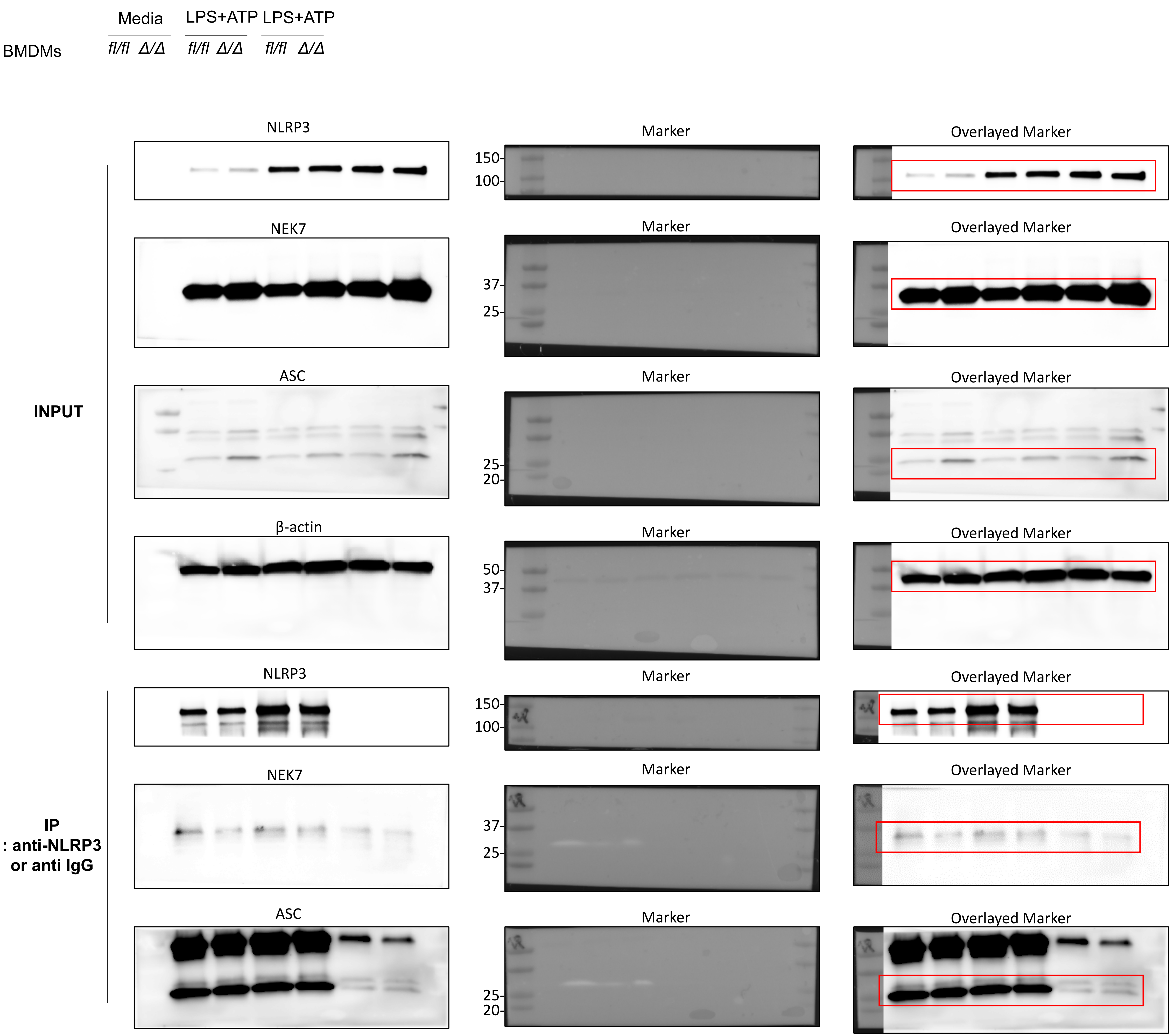

Supplementary Figure 13

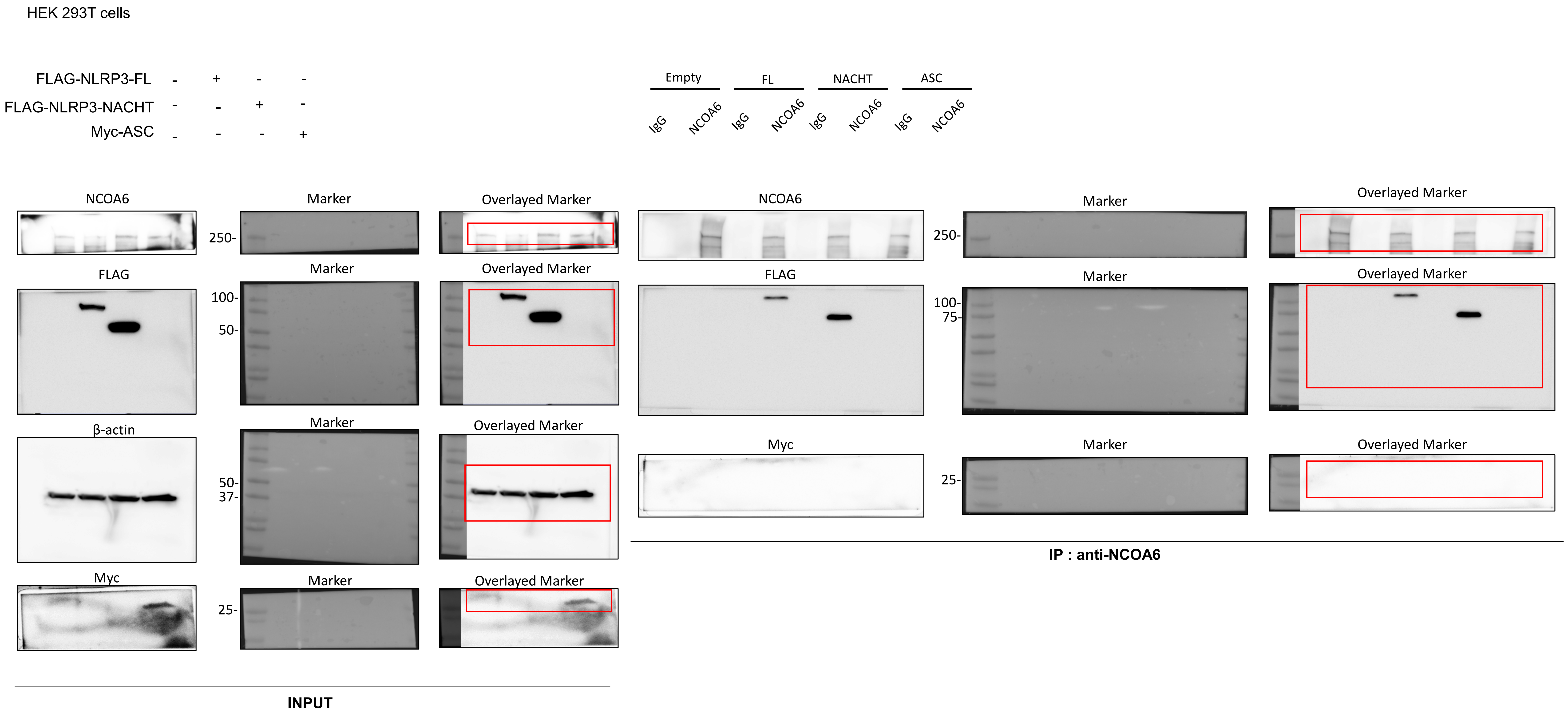

Supplement: Supplementary file 2 — Unprocessed images of gesl and western blots (revised) [file 41423_2023_1121_MOESM2_ESM.pdf]
